# Mechanochemical S*_N_* Ar on Phthalonitriles: Distinct Reactivity and Selectivity in One‐Pot Synthesis for Greener Phthalocyanines

**DOI:** 10.1002/cssc.70808

**Published:** 2026-06-13

**Authors:** Obed Rodriguez‐Perez, Daniel Langerreiter, Sandra Kaabel, Eduardo Anaya‐Plaza

**Affiliations:** ^1^ Department of Bioproducts and Biosystems Aalto University Aalto Finland; ^2^ Department of Chemistry and Materials Science Aalto University Aalto Finland; ^3^ Faculty of Medicine and Health Technology Tampere University Tampere Finland

**Keywords:** mechanochemistry, phthalocyanines, S_*N*_Ar, solid‐state synthesis

## Abstract

Traditional syntheses of functionalized phthalonitriles and phthalocyanines (Pcs) rely on solution‐phase nucleophilic aromatic substitution (S_*N*_Ar) in high‐boiling polar aprotic solvents, raising sustainability and safety concerns. Herein, we establish mechanochemical S_*N*_Ar under liquid‐assisted grinding (LAG) as a robust and versatile platform for the solvent‐minimized functionalization of phthalonitriles. Systematic optimization of base loading, ambient moisture, milling time, and LAG volume (*η*) enables highly reproducible, high‐yielding reactions at *η* values as low as 0.03 µL mg^−1^, four orders of magnitude lower than traditional synthesis. A broad scope of seven nucleophiles and nine electrophiles gives mono‐, di‐, and tetrasubstituted phthalonitriles, typically in near‐quantitative conversion and without chromatographic purification. Mechanochemical conditions also unlock distinct reactivity. Product distributions in pyridyloxy systems can be tuned between keto‐ and enol‐derived isomers by adjusting *η*, and optimized reaction of 4,5‐dichlorophthalonitrile affords quantitative and highly selective monosubstitution, enabling controlled access to asymmetric phthalonitriles. The mechanochemical synthesis of phthalonitriles is directly integrated with solid‐state cyclotetramerization, either in sequential steps or in a one‐pot S_*N*_Ar–cyclotetramerization protocol, yielding a broad range of Pc substitution patterns and chemistries. The one‐pot process provides a scalable and sustainable route to advanced Pc architectures.

## Graphical abstract

Mechanochemical nucleophilic aromatic substitution enables efficient, reproducible functionalization of phthalonitriles under minimal solvent conditions with high versatility. Integrating this process with solid‐state cyclotetramerization affords metalated and metal‐free phthalocyanines in a streamlined sustainable one‐pot workflow.

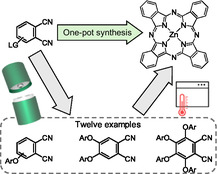

## 1 Introduction

Traditional synthetic routes to organic pigments generally rely on large amounts of toxic chlorinated and highly polar, water‐miscible solvents, making dye manufacture a significant contributor to industrial water and chemical pollution [1, 2]. Implementing solid‐state and mechanochemical synthetic strategies in pigments can substantially reduce solvent consumption, thereby mitigating environmental impact, improving water quality, and offering cost benefits for industrial processes [3]. Thus, these methodologies can contribute directly to the United Nations Sustainable Development Goals #6—Clean Water and Sanitation; and #12—Responsible Consumption and Production [4]. Among solid‐state methods, mechanochemistry occupies a prominent position. Reactions are driven by mechanical forces (grinding, shearing), which both supply energy and homogenize the reaction mixture, either under neat conditions or with a small amount of liquid (liquid‐assisted grinding, LAG) [5]. Reflecting its potential for greener chemistry, mechanochemistry has been recognized among the top ten technologies for a sustainable future by IUPAC (2019) [6] and as a powerful tool toward the European Green Deal objectives (2021) [7]. In this context, environmentally benign mechanochemical approaches have been developed for various small molecules, including molecular hosts [8, 9] and polyaromatic molecules [10, 11], and successfully applied to organic pigments such as perylene diimides [12], azo‐ [13], and BODIPY derivatives [14, 15].

With an estimated annual production of several tens of kilotonnes in the EU and close to 100 kilotonnes worldwide, phthalocyanines (Pcs) account for ≈15%–25% of the organic pigment market by volume [16], thus making Pcs a suitable target for developing greener synthetic methods. These porphyrinoids consist of four isoindole units linked by nitrogen atoms, forming an 18 *π*‐electron aromatic system [17]. This extended conjugation gives rise to intense Q‐band absorptions in the red region of the spectrum and underpins their characteristic deep blue coloration. Pcs exhibit outstanding thermal and photochemical stability, moderate‐to‐high fluorescence quantum yields, and efficient generation of singlet oxygen, among other photophysical and photochemical properties [18, 19]. Their properties can be finely tuned by incorporating a wide range of metal ions in the inner cavity and by peripheral functionalization of the macrocycle [20, 21]. Conventionally, functionalized Pcs are accessed via a two‐step sequence comprising the synthesis of substituted phthalonitrile precursors, followed by cyclotetramerization with or without the presence of a metal ion template [22, 23].

Since our report on the solid‐state synthesis of Pcs [24], there has been growing interest in greener protocols for accessing these chromophores [25]. However, the sustainable synthesis of functionalized phthalonitriles—the key precursors that govern the optical properties, supramolecular assembly, and solubility of Pcs—remains largely underexplored. Among the various strategies developed for phthalonitrile synthesis, nucleophilic aromatic substitution (S_*N*_Ar) stands out due to the wide range of nucleophiles and readily available electrophiles. In conventional solution‐based S_*N*_Ar protocols, however, high temperatures, long reaction times, and large volumes of polar aprotic solvents, such as dimethylformamide (DMF), are commonly required, raising environmental and safety concerns related to toxicity, waste generation, and energy consumption [26].

Herein, we show that mechanochemical synthesis enables highly efficient and reproducible access to functionalized phthalonitriles and their direct conversion into phthalocyanines within a single workflow. Substituted phthalonitriles are obtained from nitro‐ or halo‐activated precursors using hydroxypyridines, phenols, and thiols under ball‐milling conditions, delivering consistently high conversions (>95%, 10 examples). Careful control of key parameters, most notably the amount of the LAG agent and milling time, was critical for achieving high reproducibility, addressing a central challenge in mechanochemical synthesis. The methodology exhibits broad substrate scope and reveals clear steric and electronic effects governing reactivity in the solid state. Furthermore, the mechanochemical approach affords reaction pathways to mono‐ and disubstituted phthalocyanines that are inaccessible by conventional methods, opening access to unique Pc derivatives. The resulting functionalized phthalonitriles are subsequently transformed into both metalated and metal‐free phthalocyanines using solid‐state cyclotetramerization protocols. Importantly, integrating nucleophilic aromatic substitution and cyclotetramerization into a one‐pot mechanochemical process provides a streamlined, solvent‐minimized route to functionalized phthalocyanines, underscoring the practical and sustainability advantages of this approach.

## 2 Results and Discussion

### 2.1 Optimization of Reaction Parameters

**FIGURE 1:**
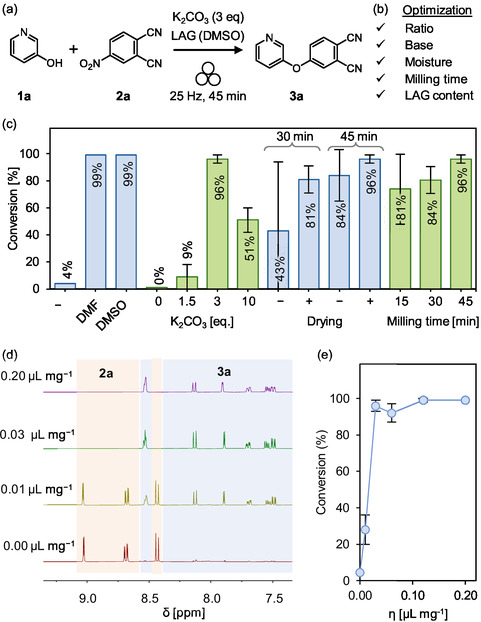
(a) General reaction scheme of the benchmark reaction by ball milling and (b) optimized parameters. (c) Screened reaction parameters and respective conversions as measured by ^1^H NMR. Average of triplicate reactions is shown with error bars representing the std. deviation. Full experimental details are shown in Tables S1–S5. (d) Partial ^1^H NMR spectra of the reaction crude after extraction, varying the amount of DMSO. Assigned peaks for **2a** and **3a** are highlighted in peach and blue, respectively. (e) Conversion of **3a** upon increase of DMSO amount from *η* = 0.0–0.20 µL mg^−1^. Other parameters were kept constant (i.e., 3 equiv of K_2_CO_3_, 45 min, predrying, 1:1 ratio of starting materials). Triplicate reactions were carried out, with error bars representing the std. deviation.

Mechanochemical S_*N*_Ar reactions, recently explored in active pharmaceutical ingredients and functional materials [27, 28, 29, 30, 31], offer particularly convenient access to substituted phthalonitriles as Pc precursors. We chose the S_*N*_Ar between 3‐hydroxypyridine (**1a**) and 4‐nitrophthalonitrile (**2a**) as a model reaction, to benchmark a mechanochemical protocol against a well‐established procedure [32, 33, 34, 35, 36]. The reaction progress was analyzed by ^1^H NMR, with conversion determined from the integrals of **3a** relative to **2a** (Figures 1d and S1). Initial experiments with **1a** (110 mg) and **2a** (200 mg) in the presence of an inorganic base (K_2_CO_3_, 10 eq, 1.6 g) and 50 µL of dry DMF or DMSO as LAG agent demonstrated complete conversion (99%, based on ^1^H NMR) to the desired product **3a** with 30 min of milling at 25 Hz in a stainless‐steel jar (Figure 1a–c). Systematic optimization under equimolar conditions revealed, however, that high and reproducible conversions could only be achieved when several key parameters were simultaneously controlled (Figure 1c). The presence of a LAG agent was essential. The reaction proceeded only marginally under solventless conditions (4% conversion), whereas the addition of a polar aprotic liquid enabled efficient S_*N*_Ar reactivity, delivering high conversions irrespective of whether dry DMSO or DMF was used. DMSO was selected for further studies due to regulatory concerns associated with the use of DMF [37]. The reaction was strictly base dependent, with no conversion observed in the absence of base (0%). Among the bases tested, K_2_CO_3_ proved particularly effective, and three eq. consistently afforded optimal performance (Figure 1c).

Importantly, controlling the moisture content of the solids emerged as a decisive factor for both conversion and reproducibility. Reactions conducted with untreated commercial reagents showed highly variable outcomes, showing a 43% ± 51% and 84% ± 19% conversion for triplicate reactions milled for 30 and 45 min, respectively. This effect is likely due to heavy clumping of the solids, affecting the milling efficiency. A simple predrying step led to a dramatic improvement, yielding reproducibly high conversions of 81% ± 10% and 96% ± 3% under the abovementioned conditions (Figure 1c). Milling time further influences the reliability of the process. Although near‐complete conversion could be achieved within 15 min, this came at the expense of reproducibility, as reflected by large standard deviations across triplicates. Extending the milling times to 45 min consistently delivered high and reproducible conversions (Figure 1c). These observations underscore the sensitivity of mechanochemical S_*N*_Ar to both water content and reaction time, particularly affecting the reproducibility. Notably, some heat can be generated by these milling conditions due to friction and collisions (up to 35°C–40°C) [38]. However, it is not straightforward to decouple the mechanical and thermal effects on the reaction without precise temperature control.

Finally, optimization of the liquid‐to‐solid ratio (*η*) [39] demonstrated that the reaction remains highly efficient under exceptionally low solvent loadings. High conversions were maintained at *η* values as low as 0.03 µL mg^−1^ (i.e., 25 µL anhydrous DMSO in benchmark conditions), with higher ratios providing only marginal improvements in reproducibility and no significant increase in conversion (Figure 1e). This result highlights the adaptability of the S_*N*_Ar reaction to minimal‐solvent, room‐temperature conditions, operating at liquid‐to‐solid ratios nearly two orders of magnitude below the typical upper limit for LAG processes (slurry conditions *η* > 1 µL mg^−1^) and four orders of magnitude from traditional reaction setups [40].

### 2.2 Expanding the Reaction Scope

**FIGURE 2:**
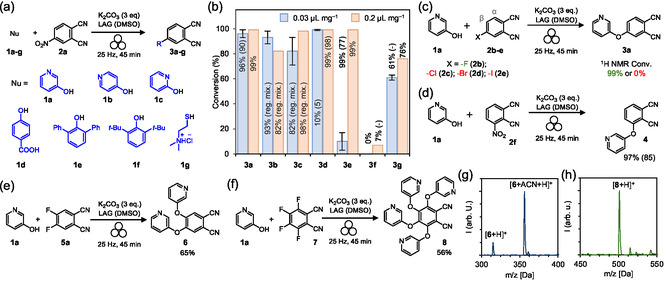
(a) General scheme of mechanochemical S_*N*_Ar reaction and scope of nucleophiles investigated. (b) Conversion rates of the reaction of **2a** with nucleophiles gathered from ^1^H NMR integral ratios at 0.03 and 0.2 µL mg^−1^ with the optimized conditions (3 equiv. K_2_CO_3_, 25 Hz, 45 min). For **3b**, **3f**, and **3g**, no predrying was done due to observed changes during predrying (color change, melting). In the case of **3b** and **3c**, the conversions given include both regioisomers (see below). The error bars represent the std. deviation. Yields are shown in parenthesis. General scheme of mechanochemical S_*N*_Ar reaction and scope of 3‐halophthalonitriles (c) and 4‐nitrophthalonitrile (d), with conversions from ^1^H NMR. General scheme of mechanochemical S_*N*_Ar reaction of **1a** and **5a** (e) and **7** (f). Electrospray ionization mass spectrometry (ESI‐MS) spectra of **6** (g) and **8** (h).

Under the optimized mechanochemical conditions—equimolar reactants ratio, three eq. of K_2_CO_3_, milling over 45 min at 25 Hz after vacuum‐drying of the reactants, in the presence of dry DMSO (*η* = 0.03 µL mg^−1^)—the reaction scope proved broad and highly informative with respect to both electronic and steric effects. Across a diverse set of nucleophiles, the S_*N*_Ar transformation consistently resulted in high conversions, with reactivity trends correlating with nucleophile structure and LAG level (0.03 and 0.2 µL mg^−1^ (Figure 2a,b and Table S6). Hydroxypyridine isomers (**1b, c**) exhibited excellent performance (>90% conversion) under minimal‐solvent conditions, showing that the reaction tolerates variation in electronic distribution within pyridine‐based nucleophiles, although regioselectivity is observed and discussed below. In contrast, nucleophiles bearing strongly electron‐withdrawing substituents showed a pronounced dependence on *η* given their relatively low reactivity. 4‐hydroxybenzoic acid (**1d**) afforded **3d** only in poor conversions of 10% ± 7% at low *η*, whereas a drastic increase to 99% conversion was observed at *η* = 0.2 µL mg^−1^. Steric effects were equally relevant. While 2,6‐diphenylphenol (**1e**) reacted efficiently, delivering consistently high conversions to **3e** (99%), the more sterically congested 2,6‐di‐*tert‐*butylphenol (**1f**) showed very limited reactivity to **3f** (7% at *η* = 0.2 μL mg^−1^). Finally, extension of the reaction scope beyond phenolic nucleophiles proved viable. A nonvolatile thiol (**1g**) results in **3g** in 75% conversion at the higher *η*, demonstrating the adaptability of the mechanochemical S_*N*_Ar platform to distinct nucleophilic cases (Figure 2b). Nucleophiles **1b**, **1f**, and **1g** showed a clear change in color upon heating, and therefore, the predrying step was not undertaken for these reactions.

Extending the scope to different electrophiles revealed a strong dependence of the mechanochemical S_*N*_Ar reactivity on both the nature and substitution pattern of the phthalonitrile substrate (Figure 2c and Table S7). Among monosubstituted phthalonitriles, the fluoro‐derivative **2b** matched the benchmark substrate **2a** in performance (99%), whereas chloro‐, bromo‐, and iodo‐analogues (**2c–e**, respectively) were completely unreactive, even under increased *η* (Figure 2c). Zirconia jars were used instead of stainless‐steel jars for fluorinated compounds to prevent corrosion, indicating that changing the jar material had no detectable impact on performance under these conditions. Lastly, changing the substitution from β‐ to α‐position proved feasible. 3‐nitrophthalonitrile (**2f**) afforded excellent conversions to the corresponding product **4**, with conversions of 97% ± 3% at *η* = 0.03 µL mg^−1^ (Figure 2d).

The methodology proved particularly powerful when applied to di‐ and tetrahalogenated phthalonitriles, giving access to multisubstituted building blocks that are essential precursors for advance phthalocyanine architectures (Figure 2e,f) [41, 42, 43]. Disubstitution in β position through S_*N*_Ar of 4,5‐difluoro‐phthalonitrile (**5a**) proceeded smoothly at *η* = 0.15 µL mg^−1^, affording **6** in 99% conversion and 65% isolated yield. In contrast, less reactive 4,5‐dichlorophthalonitrile (**5b**) displayed only limited reactivity. However, this feature and the resulting **9** were later exploited for asymmetric phthalonitrile synthesis (vide infra).

Finally, the tetrasubstituted, highly electron‐deficient substrate **7** underwent efficient reaction at *η* = 0.2 µL mg^−1^, affording **8** in a 99% conversion and 67% isolated yield. Product formation and complete substitution were confirmed by ^1^H NMR and by mass spectrometry (Figure 2g and h).

### 2.3 Nonconventional Reaction Pathways

**FIGURE 3:**
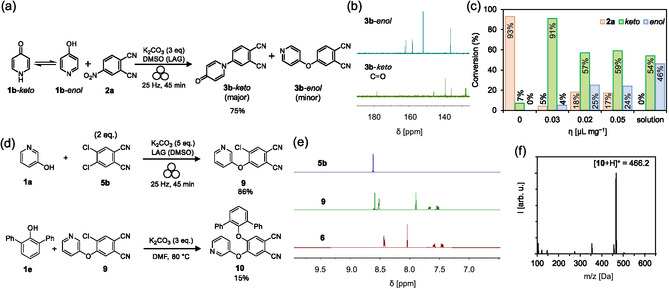
(a) General scheme of the keto/enol tautomeriaztion of **1b** and the effect in the synthesis of **3b**. (b) Partial ^13^C NMR of **3b** *‐*enol and **3b** *‐*keto showing the absence and presence of a carbonyl peak at 178 ppm. (c) Effect of LAG content in the conversion and keto/enol ratio, including solution conditions. Other parameters were kept constant (i.e., 3  eq. of K_2_CO_3_, 45 min, 1:1 ratio of starting materials). The experiment in solution was done in 10 mL DMF heating under reflux at 80°C for 16 h. Triplicate reactions were carried out for *η * = 0.03 µL mg^−1^. (d) General scheme of mechanochemical S_*N*_Ar reaction of **1a** and **5b**, yielding asymmetric product **9** (top) and subsequent solvothermal reaction with nucleophile **1e**, resulting in asymmetric phthalonitrile **10**. (e) Partial ^1^H NMR spectra showing the aromatic region used for calculating the conversions to product **9**. Starting material **5a** and disubstituted **6** are shown for reference. (f) ESI mass spectrum of **10**.

Beyond providing a greener route to phthalonitriles, one of the biggest impacts in mechanochemistry is the emergence of unique reactivities, unveiling selectivity patterns that are not observed in solution [44]. Detailed characterization of product **3b** by ^13^C NMR showed a distinct signal at ≈180 ppm, indicative of a carbonyl carbon. This unexpected result is consistent with nucleophilic attack by the keto tautomer of **1b** (**1b** *‐*keto, Figure 3a,b), rather than the **1b**‐enol form typically arising in solution. This finding established that mechanochemical conditions, particularly the presence of solvent controlled by *η*, can directly influence tautomeric reactivity. At very low *η*, reaction proceeds predominantly via the keto intermediate, yielding almost exclusively the corresponding keto isomer (**3b** *‐*keto; Figure 3c). Increasing *η* progressively shifts the product distribution toward **3b** *‐*enol. A control experiment in solution (10 mL DMF) afforded a near 1:1 mixture of isomers, highlighting the total selectivity imposed by the mechanochemical environment. A similar but attenuated trend was observed for **3c**. At low *η*, the minor isomer remained below the 5% conversion threshold, and as *η* increased, it raised to around 9% (Figure S9).

Despite its lower intrinsic reactivity in mechanochemical S_*N*_Ar, the widely used **5b** displayed a highly distinctive and synthetically valuable behavior (Figure 3d) [34, 45, 46]. Under the benchmark conditions, only limited conversion to the monosubstituted derivative was observed (**9**, 23%). Further optimization by increasing *η* to 0.78 µL mg^−1^ and employing an excess of nucleophile **1a** (2 eq.) resulted into quantitative and highly selective conversion to the monosubstituted product **9** (Figure 3e and Table S11). This outcome is particularly striking when contrasted with **2c**, which was completely unreactive under all conditions tested. This result hints that the presence of two chlorine atoms renders a sufficiently electron‐deficient ring to enable the first substitution, whereas installation of the pyridyloxy‐electron‐donating group effectively suppresses further substitution. Otherwise, steric factors can be ruled out, given the efficient disubstitution pattern obtained in **5a**. This delicate interplay enables a high selectivity toward monosubstitution, a transformation that is difficult to control in solution‐phase S_*N*_Ar, typically requiring column chromatography for purification [47, 48, 49]. Thus, we exploited this unique selectivity to design a sequential strategy that combines mechanochemical S_*N*_Ar with a subsequent thermal S_*N*_Ar step that gives access to asymmetric phthalonitriles. Using the sterically hindered 2,6‐diphenylphenol as the second nucleophile, the asymmetric derivative **10** was successfully obtained (Figure 3f), albeit in modest yield (15%) after chromatographic purification.

Altogether, these results demonstrate the twofold uniqueness of the mechanochemical approach, resulting from the uniquely low liquid contents. On the one hand, tautomer‐dependent S_*N*_Ar pathways arise, enriching the reactivity landscape. On the other hand, limited reactivity of **5b** under mechanochemical conditions can be transformed into a powerful selectivity advantage, opening a unique and controlled route toward asymmetric phthalonitrile precursors for advanced Pc derivatives.

### 2.4 Solid‐state Synthesis of PCS

**FIGURE 4:**
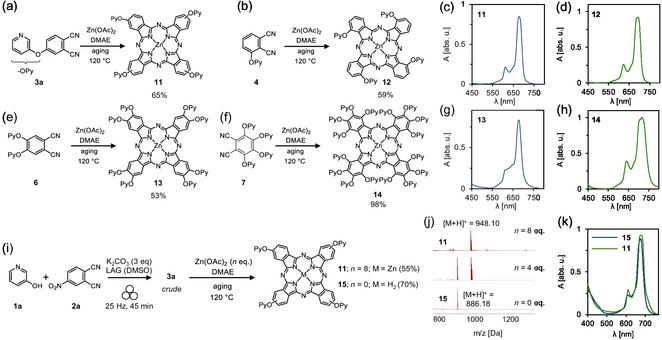
General synthesis of tetrasubstituted Pcs **11** (a) and **12** (b) by aging. UV–Vis spectrum of Pc **11** (c) and **12** (d) in DMSO, showing sharp Q‐bands. General synthesis of octasubstituted Pc **13** and hexadecasubstituted Pcs **12** (e and f, respectively) by aging. UV–Vis spectrum of Pc **13** (g) and **14** (f) in DMSO. The latter shows broader and redshifted Q‐band, likely due to slight deformations of the aromatic planarity. (i) General scheme of mechanochemical S_*N*_Ar followed by cyclotetramerization in a one‐pot synthesis of metalated and metal‐free Pcs **11** and **15**, respectively. (j) MALDI spectra stack of one‐pot reaction between **1a** and **2a** with varying amounts of Zn(OAc)_2_. (k) UV–Vis spectra of **11** and **15** produced by the one‐pot method in the 400–750 nm region.

To complete an overall greener synthetic route and validate the potential of solid‐state synthesis of phthalonitriles, heating during [50] or after milling [24] was considered. Due to the limitations of establishing accurate temperature control during milling, we chose the latter, based on our previously reported solid‐state synthesis of Pcs. Briefly, the selected phthalonitrile, an appropriate metal salt as cyclotetramerization template, and 2‐(dimethylamino)‐ethanol (DMAE) as LAG agent were first milled for 5 min to ensure intimate mixing, and the resulting solid was then aged at 120°C for 48 h. Four representative phthalonitriles were selected to represent each class: a β‐ and α‐substituted phthalonitrile (**3a** and **4**; Figure 4a,b, respectively), as well as a di‐ and tetrasubstituted derivatives (**6** and **7**) (Figure 4e,f). The sequential milling‐aging protocol yielded the characteristic deep blue color of Pcs after few minutes, although the cyclotetramerization reaction was carried out to completion over 48 h [24]. The well‐known insolubility and aggregation proneness of these Pcs limit in‐solution characterization by NMR methods, with spectra showing large peak broadening (see Figures S67, S70, S73, S76) [51]. However, matrix‐assisted laser desorption/ionization (MALDI) mass spectrometry and UV–Vis spectroscopy confirmed the formation of the desired products (Figure 4c,d,g,h).

Finally, a one‐pot synthesis integrating phthalonitrile formation and subsequent Pc formation was developed as proof of concept to simplify the reaction setup and eliminate purification steps, contributing to the sustainability of the process (Figure 4i) [52]. Under optimized benchmark conditions (*η* = 0.12 µL mg^−1^), the S_*N*_Ar step reached quantitative conversions confirmed by TLC, enabling direct progression to cyclotetramerization by adding anhydrous Zn(OAc)_2_ (4 eq. of Zn per phthalocyanine) and DMAE, without isolation of the functionalized phthalonitrile **3a**. The resulting deep blue solid was purified by washings of water and hot methanol. UV–Vis spectroscopy and MALDI confirmed the formation of Pcs (Figure 4j,k), although the latter revealed the simultaneous formation of both the ZnPc and metal‐free H_2_Pc (**11** and **15**, respectively). Increase of Zn(OAc)_2_ to 8 eq. yielded **11** quantitatively in 55% yield, while complete absence of the metal template results in quantitative formation of **15**, a metal‐free Pc not accessible by previous solid‐state methodologies in 70% yield (Figure 4j) [24]. The formation of the metal‐free Pc could be due to the excess of K_2_CO_3_ in the reaction media, which likely deprotonates DMAE, initiating the reaction [25]. Control experiments by only adding DMAE to the pure phthalonitrile and aging it in the oven, or in the presence of potassium cations from KCl rules out the templating role of K^+^ (Figures S12 and S13, respectively).

E‐factors were calculated for the solid‐state bench mark reactions, i.e., S*N*Ar, cyclotetramerization, and the one‐pot reaction (E‐factor = 48.8 for the one‐pot reaction, see Supporting Information (SI)). This result highlights the environmental benefits of the mechanochemical one‐pot methodology, resulting into at least a fivefold decrease of waste when compared to similar reactions in solution (E‐factor = 257.7) [52]. Even implementing organic solvent recycling (E‐factor = 86.9), our methodology reduces the E‐factor to roughly half. Altogether, the selected one‐pot method serves as a proof‐of‐concept demonstration for a greener synthetic approach to engineered architectures of Pcs.

## 3 Conclusion

In summary, we have established mechanochemical S_*N*_Ar as a robust and versatile platform for the functionalization of phthalonitriles under LAG conditions. Systematic exploration of key reaction parameters—including reactant ratios, base loading, ambient moisture, milling time, and the nature and amount of liquid—enabled highly reproducible reactions without compromising efficiency. Screening seven nucleophiles and nine electrophiles provided access to mono (α and β)‐, di‐, and tetrasubstituted phthalonitriles, demonstrating the applicability of mechanochemistry to the main substitution patterns relevant to current Pc chemistry. In most cases, the reactions proceeded to near‐quantitative conversion, allowing purification by simple extraction and avoiding chromatographic separations.

Beyond providing a greener route, this study illustrates a key advantage of mechanochemistry: the ability to access distinct reaction pathways and products. We demonstrate that the product distribution in pyridyloxy systems can be tuned between keto‐ and enol‐derived isomers by adjusting the LAG volume, and that optimized reaction of 4,5‐dichlorophthalonitrile affords quantitative and highly selective monosubstitution, opening a controlled route to asymmetrically substituted phthalonitriles and their derived Pcs. Finally, we have coupled the mechanochemical synthesis of functionalized phthalonitriles with solid‐state cyclotetramerization, either in sequential steps or in a one‐pot S_*N*_Ar–cyclotetramerization protocol. The latter transforms commercially available phthalonitriles and nucleophiles into metal‐free or metalated phthalocyanines, by a simple and straightforward methodology. This approach avoids the use of intermediate separation and chromatography methods, which enhances sustainability while delivering Pc derivatives that were inaccessible in our previous solid‐state approach.

We envision future work on exploring temperature‐controlled milling, which may further reduce reaction times, enhance reproducibility, and expand the range of accessible substrates and one‐pot transformations.

## Author Contributions

**Obed Rodriguez‐Perez:** investigation, methodology, visualization, writing – original draft, writing – review & editing. **Daniel Langerreiter:** methodology. **Sandra Kaabel:** writing – review & editing, formal analysis, data curation, validation, visualization. **Eduardo Anaya‐Plaza:** conceptualization, funding acquisition, validation, visualization, writing – review & editing, formal analysis, project administration, data curation, supervision, resources.

## Funding

This study was supported by the Academy of Finland (341057, 359903, and 361545).

## Conflicts of Interest

The authors declare no conflicts of interest.

## Supporting information

Supplementary Material

## Data Availability

The data that support the findings of this study are available in the SI of this article.
