# Supplementary material for: Mechanochemical S N Ar on Phthalonitriles: Distinct Reactivity and Selectivity in One‐Pot Synthesis for Greener Phthalocyanines
Source: ChemSusChem. 2026 Jun 13;19(11):e70808. doi: 10.1002/cssc.70808 (PMC13263912; doi:10.1002/cssc.70808)
Supplement: Supplementary file 1 — Supplementary Material [file CSSC-19-e70808-s001.pdf]

Supporting Information

Mechanochemical S_NAr on Phthalonitriles: Distinct Reactivity and Selectivity in One-Pot Synthesis for Greener Phthalocyanines

Obed Rodriguez-Perez,[a] Dr. Daniel Langerreiter,[a] Prof. Sandra Kaabel,[b] and Prof. Eduardo Anaya-Plaza*[a][c]

[a] MSc. Obed Rodriguez-Perez, Dr. Daniel Langerreiter, Prof. Eduardo Anaya-Plaza
Department of Bioproducts and Biosystems
Aalto University
Kemistintie 1, 00016 AALTO, Finland

[b] Prof. Sandra Kaabel
Department of Chemistry and Materials Science
Aalto University
Kemistintie 1, 00016 AALTO, Finland

[c] Prof. Eduardo Anaya-Plaza
Faculty of Medicine and Health Technology
Tampere University
Korkeakoulunkatu 3, Hervanta Campus, 33720 Tampere, Finland
E-mail: eduardo.anayaplaza@tuni.fi

1. Table of Contents

2. Materials and Equipment 2
3. Experimental Details and Results 2
3.1. Conversion rate calculation 2
3.2. Detailed data of optimization experiments for benchmark reaction 3
3.2.1. Preliminary experiments 3
3.2.2. Effect of pre-treatment on the reaction 4
3.2.3. Milling with variation of K_2CO_3 equivalents 4
3.2.4. Milling with variation of DMSO 5
3.2.5. Milling with variation of milling time 6
4. General procedure and characterization of products 7
4.1. General procedure for mechanochemical S_NAr of phthalonitriles. 7
4.2. Scope of nucleophiles 7
4.3. Scope of phthalonitrile substrates 9
4.4. Selected phthalocyanines 11
4.5. One-pot synthesis of phthaocyanines 12
5. LAG influence on keto-enol tautomerization of 4-hydroxypyridine 13
6. LAG influence on keto-enol tautomerization of 2-hydroxypyridine 15
7. Unsuccesful synthesis of 3f 16
8. Optimization of the synthesis of 9 16
9. Influence of potassium carbonate and zinc acetate in the synthesis of 11 and 15 17
10. E-factor calculations 20
11. Structural characterization spectra 22
12. References 54

2. Materials and Equipment

All chemicals were bought from commercial suppliers and used without further purification: 4-nitrophthalonitrile, 3-nitrophthalonitrile, 4-fluorophthalonitrile, 4-bromophthalonitrile, 4-chlorophthalonitrile, 4,5-dichlorophthalonitrile, 4,5-difluorophthalonitrile, tetrafluorophthalonitrile, 3-hydroxypyridine, 4-hydroxypyridine, 2-hydroxypyridine, 4-hydroxybenzoic acid, 2,6-diphenyphenol, 2,6-di*tert*butylphenol (TCI Chemicals), 2-(dimethylamino)ethanethiol hydrochloride, potassium carbonate, magnesium sulfate, zinc acetate, anhydrous dimethylsulfoxide, anhydrous dimethylformamide (Sigma Aldrich), 4-chlorophthalonitrile (FluoroChem), 2-(dimethylamino)-ethanol, ethyl acetate, dichloromethane, methanol (Fisher Scientific), dimethylsulfoxide-d_6 and d-methanol (Eurisotop).

The ball milling was carried out in a Retsch MM 400 mixer mill with frequencies of 25 or 30 Hz in stainless steel jars (14 mL) with single stainless-steel ball (4.0 g) from InSolido Technologies d.o.o.. For fluorinated compounds, the ball-milling was carried out in zirconia jars (10 mL) with single zirconia ball (3.0 g) from Retsch. All solid compounds were weighed on the Satorius Entris 224i-1S analytical scale. The liquids used for the reaction were pipetted via Eppendorf Research Plus pipettes (1-10 µl; 10-100 µl; 100-1000 µl). The aging experiments were performed in the Binder FED-53 (120 °C). Column chromatography, when utilized, was carried out using high purity grade silica gel with 60 Å pore size, 40-63 µm particle size (Sigma Aldrich)

The products were analysed by ^{1}HNMR (400 MHz), ^{13}CNMR (101 MHz) adn^{19}FNMR (when applicable) spectroscopy. The measurements were performed in Bruker NMR Spectrometer AV III 400 and Bruker NMR Spectrometer AV NEO 400. The chemical shifts are given in ppm and the solvent signal can be found at 2.50 ppm (^{1}HMNR) and 39.52 ppm (^{13}CNMR) (d6- DMSO). Mass spectrometry was conducted with Thermo Scientific ISQ EC ESI-MS for phthalonitriles. For phthalocyanines, the characterization was done with Bruker MALDI TOFF, dissolving the sample in THF with no matrix. The UV-Vis measurements from the phthalocyanines were carried out with Agilent Cary 60 UV-Vis in DMSO.

3. Experimental Details and Results

3.1. Conversion rate calculation

The peaks were integrated and normalized to peak **a**. The conversions were calculated from the average value of the peaks of the phthalonitrile starting material **(2a-e)** and the substituted product (**3a-j**) following the equation below (*Eq. 1*). The nucleophile is not taken into account because it is in part extracted out of the organic phases. However, overlapping peaks with **1a** such as **e** are not considered in the calculations.

$$Conv.\mathbf{3}\ (\%) = \frac{\bar{I}_3}{\bar{I}_3 + \bar{I}_2} \qquad \textit{Eq. S1}$$

where *I* represents the averaged integrals of the compounds numbered.

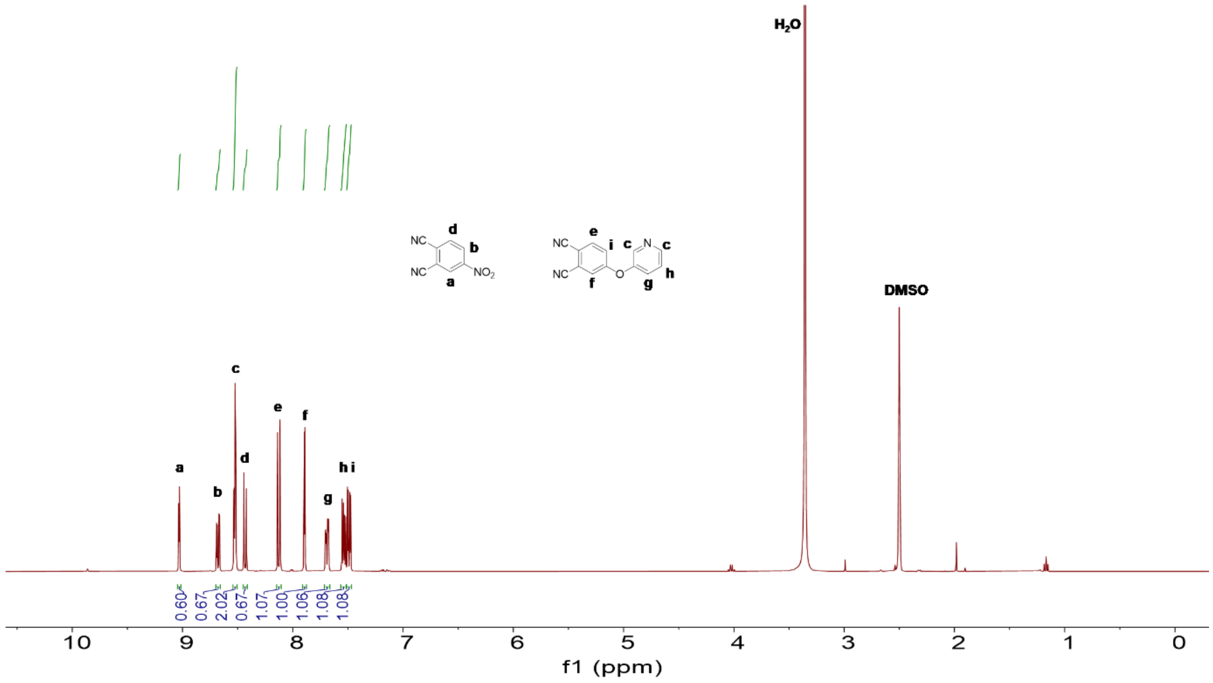

Figure S1: Model 1HNMR spectrum for peak assigning demonstrating how the conversion rate was calculated. The sample parameters are η = 0.01 µL mg^{-1} DMSO, 45 min, 10 eq K_2CO_3.

Where the crude was not purified by column chromatography, the yield was calculated by extrapolating from NMR conversions taking the input and output masses into account

$$Yield\ (\%) = \frac{m\ (crude) \times \%\ mass\ (3)}{n\ (2) \times M\ (3)} \qquad \textit{Eq. S2}$$

Where *m (crude)* is the mass of the crude after extraction, *% mass (3)* represents the mass percent of product from the crude calculated using the conversions from NMR and the molar masses

$$\%\ mass\ (3) = \frac{Conv.(3) \times M(3)}{Conv.(3) \times M(3) + \%\ (2) \times M(2) + \%\ (1) \times M(1)} \qquad \textit{Eq. S3}$$

n (2) is the amount of moles of the limiting reactant, calculated as the fraction of 2 from the input mass over the molar mass of 2, and M (3) is the molar mass of 3

3.2. Detailed data of optimization experiments for benchmark reaction

4-nitrophthalonitrile (**2a**) and 3-hydroxypiridine (**1a**) were pre-treated by placing in the vacuum oven at 40 °C for 1 hour. The starting materials and anhydrous K_2CO_3 were added to a stainless steel milling jar containing a single stainless steel ball, to which the LAG agent was added via pipette. The mixture was milled for 15-45 minutes at 25 Hz. The full batch was dissolved in 40 mL of water and 40 mL EtOAc. The aqueous phase was extracted with 3 x 40 mL EtOAc. The organic layers were combined and washed with 100 mL brine, dried over anhydrous $MgSO_4$ and the solvent evaporated by rotary evaporator, yielding **3a** as a crude beige solid that was analysed without further purification.

3.2.1. Preliminary experiments

Table S1: Reaction conditions of preliminary experiments with varying LAG agent

Entry	Mass **1a** (mg)	Mass **2a** (mg)	V (µL)	LAG (µL mg^{-1})	Milling time	K_2CO_3 equiv.	Conversion (%)
1[a]	101	55	0	0	30	10	4
2[a]	223	149	50	0.03	30	10	99
3[a][b]	205	142	50	0.03	30	10	99

[a] No pre-treatment was done. 1a in 1.3 equiv. [b] DMF was used as LAG instead of DMSO.

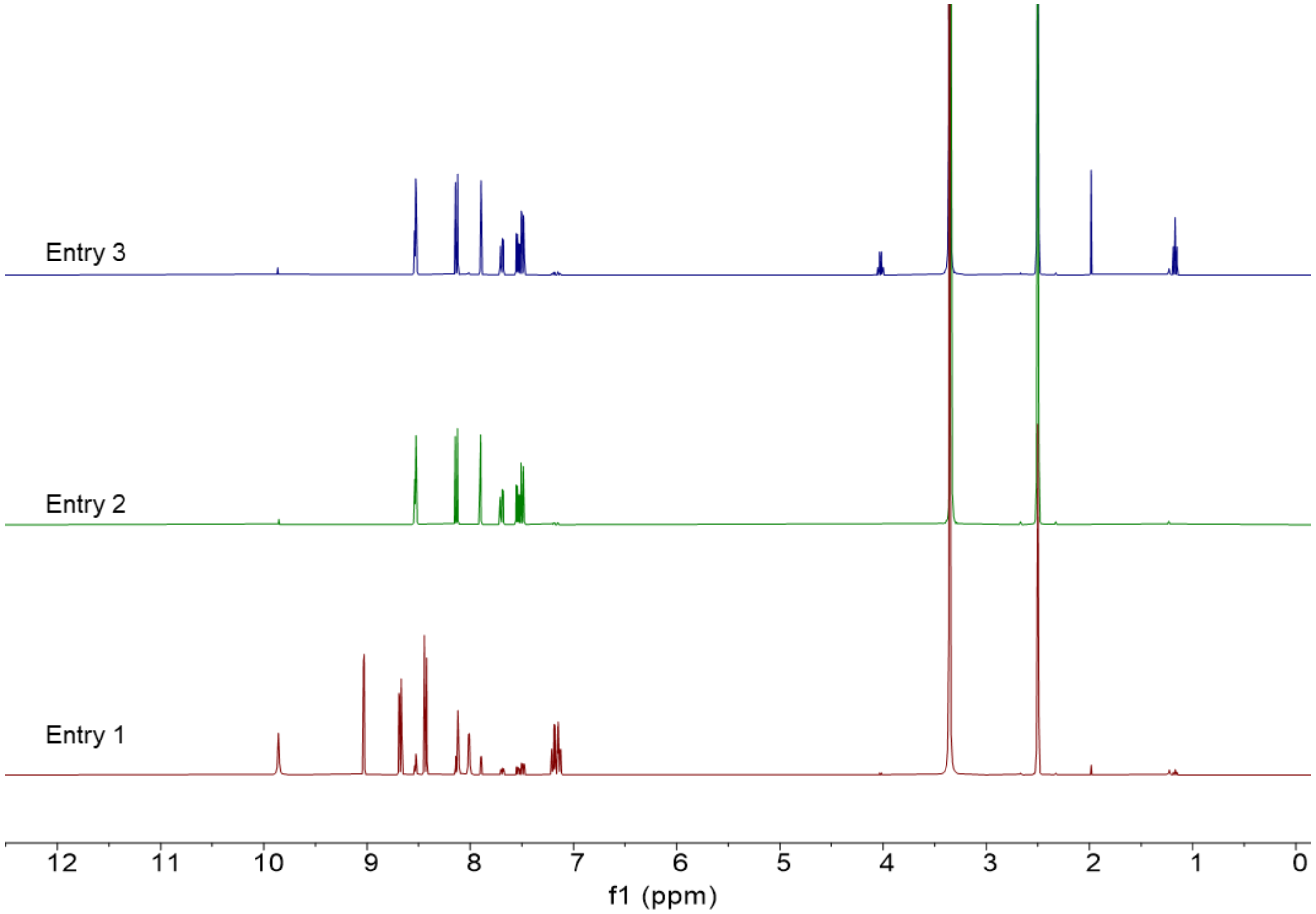

Figure S2: ^{1}H spectra for the synthesis of **3a**, showing the reaction outcome in dependence of the type of LAG agent. Experimental details are shown in **Table S1.**

3.2.2. Effect of pre-treatment on the reaction

Table S2: Reaction conditions comparison of triplicate experiments, with and without pre-treatment. All experiments were done with 200 mg of **2a**, 110 mg of **1a,** 480 mg of K_2CO_3 and 25 µL DMSO**.**

Entry	LAG (µL mg^{-1})	Milling time	K_2CO_3 equiv.	Conversion (%)
4[a]	0.03	30	3	43 ± 51
5	0.03	30	3	81 ± 10
6[a]	0.03	45	3	84 ± 19
7	0.03	45	3	96 ± 3

[a] No pre-treatment was done.

3.2.3. Milling with variation of K_2CO_3 equivalents

Table S3: Reaction conditions of experiments with varying equivalents of K_2CO_3. All experiments were done with 200 mg of **2a**, 110 mg of **1a** and 25 µL DMSO, except entry 4, which had 143 mg of **1a.** K_2CO_3 was used in the following quantities: 0 equiv. (0 mg), 1.5 equiv. (239 mg), 3 equiv. (480 mg), and 10 equiv. (1604 mg). Triplicates were run for 1.5-10 equiv.

Entry	LAG (µL mg^{-1})	Milling time	K_2CO_3 equiv.	Conversion (%)
8[a]	0	30	0	0
9	0.04	45	1.5	9 ± 9
7	0.03	45	3	96 ± 3
10	0.01	45	10	51 ± 9

[a] No pre-treatment was done.

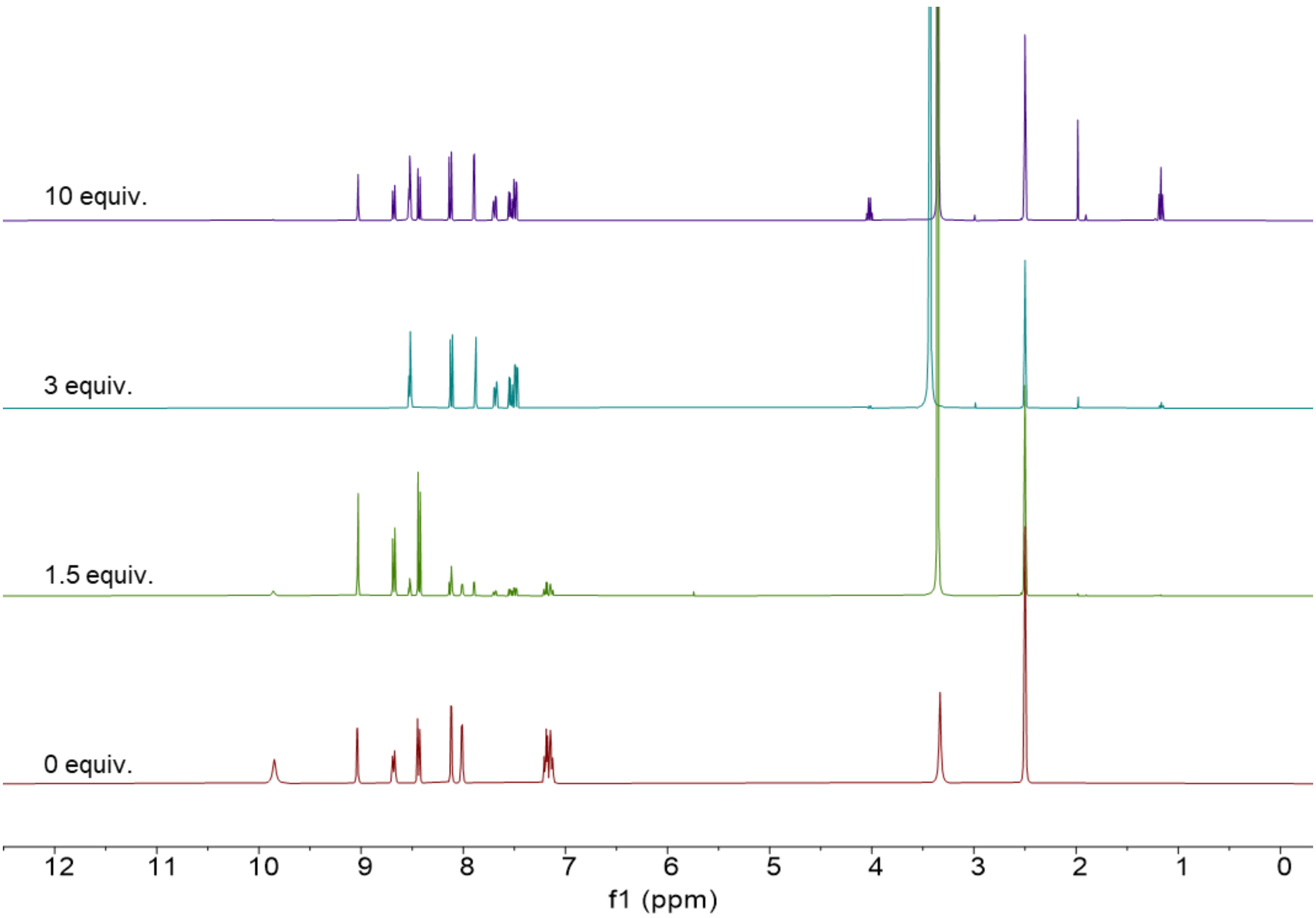

Figure S1: ^{1}H spectra for the synthesis of **3a**, showing the reaction outcome in dependence of the equivalents of K_2CO_3. Experimental details are shown in **Table S3.**

3.2.4. Milling with variation of DMSO

Table S4: Reaction conditions of experiments with varying the amount of DMSO. All experiments were done with 200 mg of **2a**, 110 mg of **1a,** and 480 mg of K_2CO_3 except entry 1 (see Table S1). DMSO was used in the following volumes: 0, 10, 25, 50, 100 and 200 µL. Triplicates were performed for 0.01 – 0.12 $\mu L\ mg^{-1}$

Entry	LAG ($\mu L\ mg^{-1}$)	Milling time	K_2CO_3 equiv.	Conversion (%)
11[a]	0	30	10	4
12	0.01	45	3	28 ± 8
7	0.03	45	3	96 ± 3
14	0.06	45	3	92 ± 5
15	0.12	45	3	99 ± 1
16	0.20	45	3	99

[a] No pre-treatment was done. 1a in 1.3 equiv.

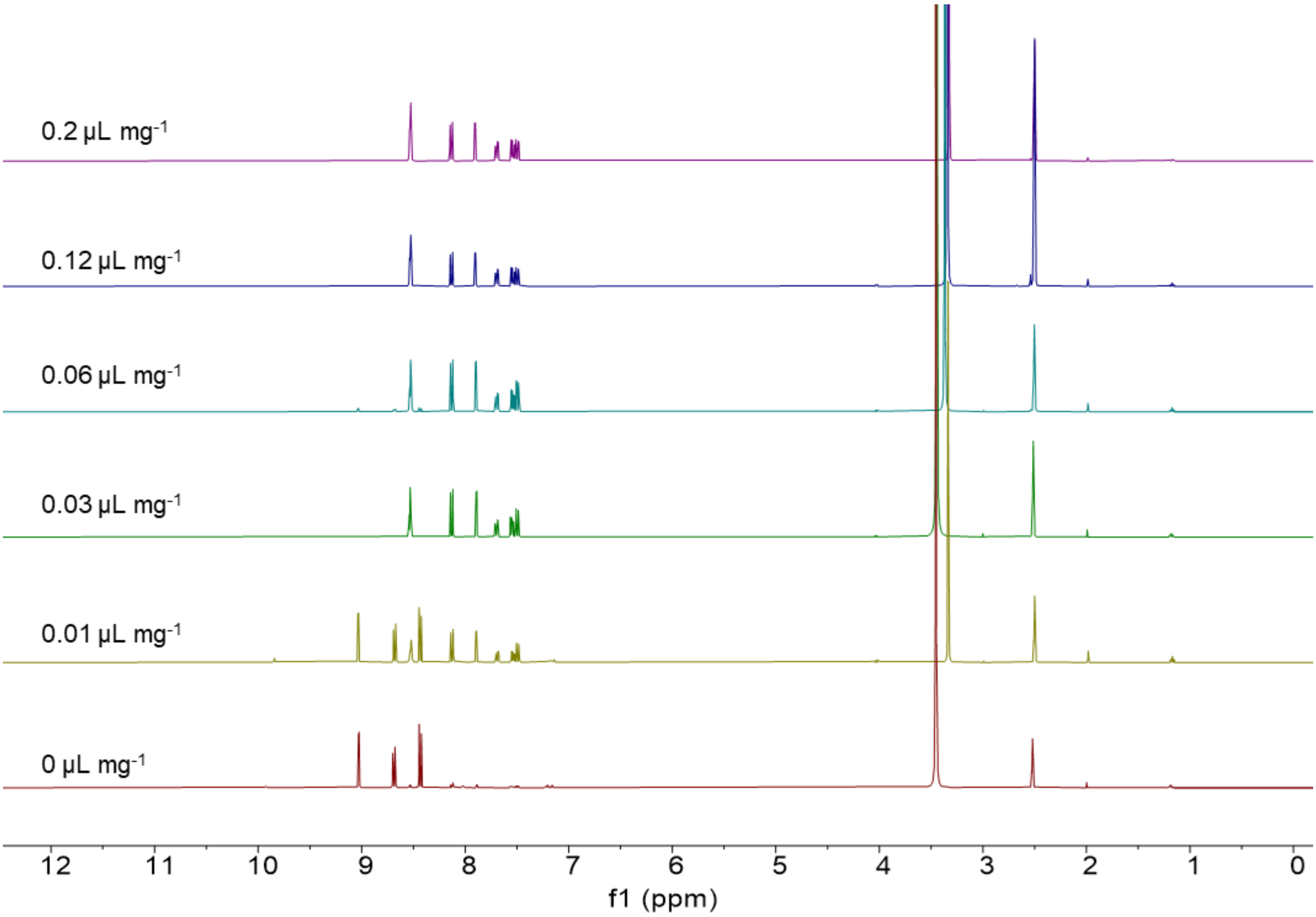

Figure S4: ^{1}H spectra for the synthesis of **3a**, showing the reaction outcome in dependence of the amount of DMSO. Experimental details are shown in **Table S4.**

3.2.5. Milling with variation of milling time

Table S5: Reaction conditions of experiments with varying milling time. All experiments were done with 200 mg of **2a**, 110 mg of **1a,** 480 mg of K_2CO_3 and 25 µL DMSO. Triplicates were performed for all experiments

Entry	LAG (µL mg^{-1})	Milling time	K_2CO_3 equiv.	Conversion (%)
17	0.03	15	3	74 ± 26
5	0.03	30	3	81 ± 10
7	0.03	45	3	96 ± 3

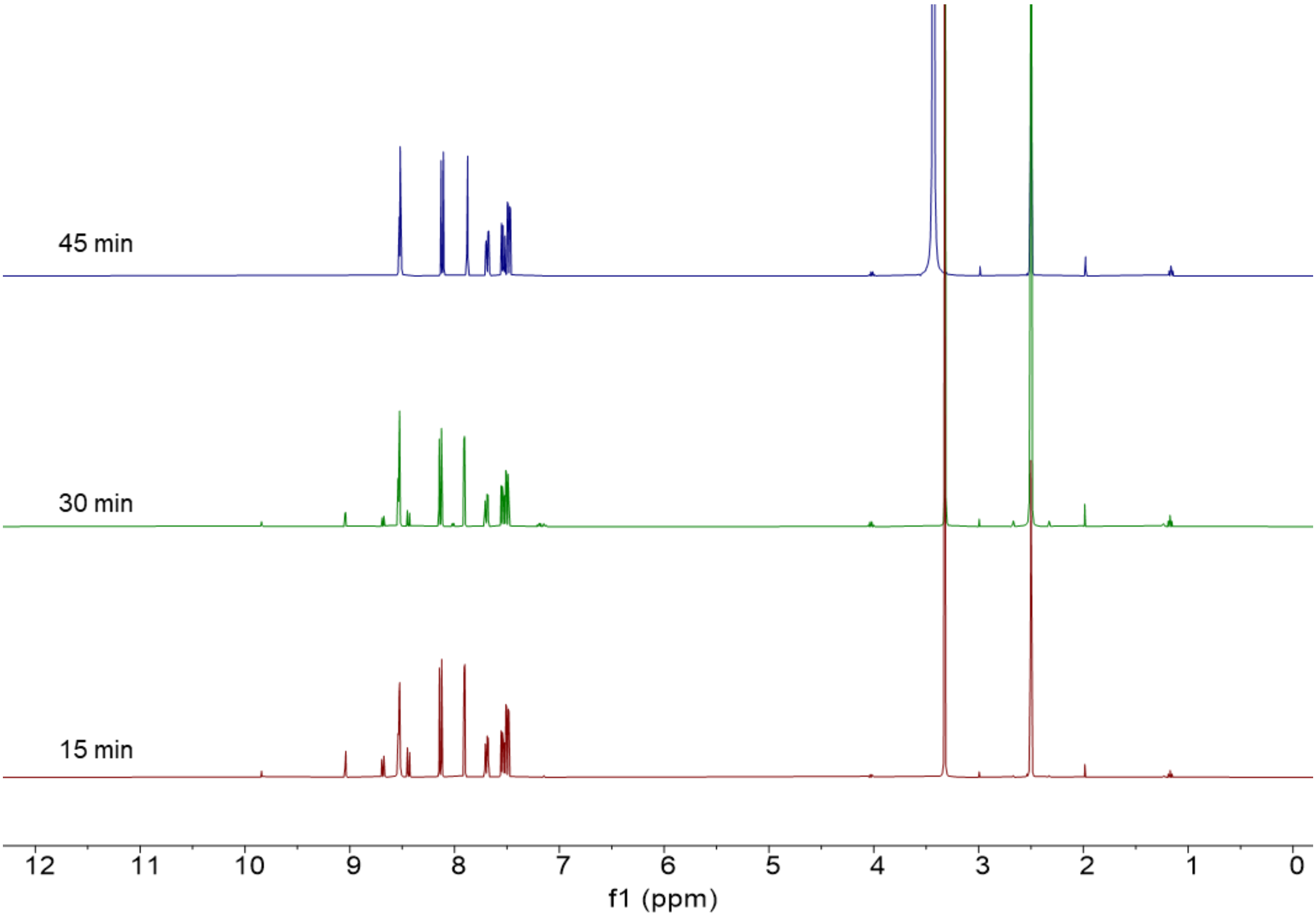

Figure S5: ^{1}H spectra for the synthesis of **3a**, showing the reaction outcome in dependence of the milling time. Experimental details are shown in **Table S5.**

4. General procedure and characterization of products

4.1. General procedure for mechanochemical S_NAr of phthalonitriles.

The phthalonitrile (1 eq) and the nucleophile (1 eq) were typically pretreated by placing in a small vial and kept in the vacuum oven at 40 °C for 1 hour, unless otherwise stated. The solid mixture was added to a stainless steel milling jar containing a single stainless steel ball with anhydrous K_2CO_3 (3 eq.) and dry DMSO (0.03 - 0.2 μL mg^{-1}). The mixture was milled for 45 minutes at 25 Hz. The full batch was dissolved in 40 mL of water and 40 mL of ethyl acetate/dichloromethane (DCM), after which the aqueous phase was washed 3 x 40 ml. The combined organic layers were washed with 100 ml brine, dried over anhydrous $MgSO_4$ and evaporated by rotary evaporator, yielding a crude solid that was analysed without further purification.

4.2. Scope of nucleophiles

Table S6: Nucleophile scope: Reaction of 2a with selected nucleophiles at η = 0.03 μL mg-1 and 0.2 μL mg-1 LAG conditions. All reactions started with 200 mg of 2a and 1 equiv of nucleophile using DMSO as LAG agent and 3 equivs.- of K_2CO_3 as a base, pretreating the starting materials by drying in the vacuum oven and milling for 45 minutes, unless otherwise stated.

Nucleophile	Product	Conversion (%) η = 0.03 μL mg^{-1}	Conversion (%) η = 0.2 μL mg^{-1}	Yield (%)[a] η = 0.03 μL mg^{-1}
1a	3a	96 ± 3	99	90
1b[b],[c]	3b	93 ± 5	82	69
1c[c]	3c	82 ± 11	98	65
1d	3d	10 ± 7	99	98[d]

1e	3e	99 ± 1	99	77
1f[b]	3f	0	7	0
1g[b]	3g	61 ± 2	76	38

[a] The yield was calculated for one experiment based on the ^{1}HNMR conversions as explained above [b] No pretreatment was done due to color change (**1b**), low melting point (**1f**), and thiol volatility (**1g**) [c] Conversions include both keto and enol isomers. [d] The yield reported corresponds to the experiment at η = 0.2 µL mg^{-1}. The experiment at η = 0.03 µL mg^{-1} had a 5% yield.

4-(pyridin-3-yloxy)phthalonitrile (**3a**)

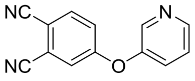

3a

Synthesised from 4-nitrophthalonitrile (200 mg, 1.16 mmol) and 3-hydroxypyridine (110 mg, 1.16 mmol) in the presence of K_2CO_3 (479 mg, 3 equiv.) and 25 µL DMSO (0.03 µL mg^{-1}) according to the general procedure, affording **3a** as a beige solid (230 mg, 90% yield) (99% conversion from ^{1}HNMR) ^{1}H NMR (400 MHz, DMSO): δ 8.53 (m, 2H), 8.12 (d, 1H), 7.88 (d,1H), 7.69 (ddd,1H), 7.53 (ddd,1H), 7.48 (dd,1H). ^{13}C NMR (101 MHz, DMSO) δ 160.45, 150.79, 146.77, 142.27, 136.39, 127.97, 125.19, 122.98, 122.48, 116.85, 115.83, 115.32, 108.93. ESI-MS: m/z calculated for $C_{13}H_8N_3O^+$ [M+H$^+$]: 222.1; found 222.1.

4-(4-oxopyridin-1(4H)-yl)phthalonitrile (**3b-keto**)

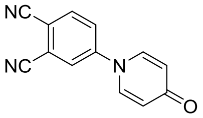

3b-keto

Synthesised from 4-nitrophthalonitrile (200 mg, 1.16 mmol) and 4-hydroxypyridine (110 mg, 1.16 mmol) in the presence of K_2CO_3 (479 mg, 3 equiv.) and 25 µL DMSO (0.03 µL mg^{-1}) according to the general procedure with no pre-treatment and extracted with 10% MeOH in DCM affording **3b-keto** as a beige solid (182 mg, 75% yield) (97% conversion) ^{1}H NMR (400 MHz, DMSO) δ 8.49 (d,1H), 8.33 (d, 1H), 8.14 (m, 3H), 6.30 (m, 2H). ^{13}C NMR (101 MHz, DMSO) δ 177.66, 145.55, 139.00, 135.59, 127.60, 127.13, 118.35, 116.22, 115.53, 115.31, 112.76. ESI-MS: m/z calculated for $C_{13}H_8N_3O^+$ [M+H$^+$]: 222.1; found 222.1.

4-(pyridin-4-yloxy)phthalonitrile (**3b-enol**)

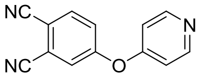

3b-enol

Side product from the synthesis of **3b** in the previous reaction**.** Synthesised from 4-nitrophthalonitrile (200 mg, 1.16 mmol) and 4-hydroxypyridine (110 mg, 1.16 mmol) in the presence of K_2CO_3 (479 mg, 3 equiv.) and 10 mL DMF. The solution was left stirring overnight at 80 °C, after which the salt was dissolved in 30 mL water and the mixture was extracted with 3 x 30 ml 10% MeOH in DCM. The crude was a mixture of keto and enol products in nearly 1:1 ratio. The enol was collected by flash column chromatography with 4% MeOH in DCM as eluent. ^{1}H NMR (400 MHz, DMSO) δ 8.58 (d, 2H), 8.19 (d, 1H), 8.03 (d,1H), 7.66 (dd, 1H), 7.15 (m, 2H). ^{13}C NMR (101 MHz, DMSO) δ 162.28, 161.71, 158.08, 152.02, 136.50, 125.13, 124.88, 117.07, 115.68, 115.21, 113.77, 110.54. ESI-MS: m/z calculated for $C_{13}H_8N_3O^+$ [M+H$^+$]: 222.1; found 222.1.

4-(2-oxopyridin-1(2H)-yl)phthalonitrile (**3c)**

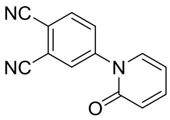

3c

Synthesized from 4-nitrophthalonitrile (200 mg, 1.16 mmol) and 2-hydroxypyridine (110 mg, 1.16 mmol) in the presence of dry K_2CO_3 (479 mg, 3.48 mmol, 3 eq) and 25 µL DMSO (0.03 µL mg^{-1}) according to the general procedure and extracted with 5% MeOH in DCM affording the crude as a beige solid (202 mg, 65% yield) (82% conversion). ^{1}H NMR (400 MHz, DMSO) δ 8.38 (d, J = 2.1 Hz, 3H), 8.30 (d,1H), 8.07 (dd, 1H), 7.74 (dd, 1H), 7.56 (td, 1H), 6.54 (d,1H), 6.40 (td,1H)**.** ^{13}C NMR (101 MHz, DMSO) δ 161.13, 144.89, 141.92, 138.45, 135.30, 133.13, 133.00, 121.19, 116.03, 115.88, 115.77, 114.46, 106.92. ESI-MS: m/z calculated for $C_{13}H_8N_3O^+$ [M+H$^+$]: 222.1; found 222.1.

4-(3,4-dicyanophenoxy)benzoic acid (**3d**)

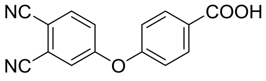

3d

Synthesised from 4-nitrophthalonitrile (200 mg, 1.16 mmol) and 4-hydroxybenzoic acid (160 mg, 1.16 mmol) in the presence of dry K_2CO_3 (479 mg, 3.48 mmol, 3 eq) and 170 µL DMSO (0.2 µL mg^{-1}) according to the general procedure. After milling, the crude was dissolved in water and concentrated HCl was added until the pH was 1, after which precipitation was observed. The precipitate was filtered by vacuum filtration, affording 300 mg of the pure product as a white solid (98% yield). ^{1}H NMR (400 MHz, DMSO) δ 8.15 (d, 1H), 8.03 (d, 2H), 7.93 (d, 1H), 7.54

(dd, 1H), 7.27 (d, 2H). ^{13}C NMR (101 MHz, DMSO) δ 166.59, 159.89, 157.93, 153.73, 136.47, 132.04, 123.87, 123.43, 119.64, 116.92, 115.87, 109.30. ESI-MS: m/z calculated for $C_{15}H_9N_2O_3^+$ [M+H$^+$]: 265.1; found 265.1

4-([1,1':3',1''-terphenyl]-2'-yloxy)phthalonitrile (**3e**)

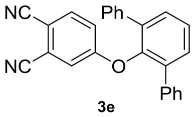

3e

Synthesised from 4-nitrophthalonitrile (150 mg, 0.87 mmol) and 2,6-diphenylphenol (213 mg, 0.87 mmol) in the presence of dry K_2CO_3 (359 mg, 2.6 mmol, 3 eq) and 25 µL DMSO (0.03 µL mg^{-1}) according to the general procedure, affording the crude product as a beige solid (250 mg, 77% yield) (99% conversion). ^1H NMR (400 MHz, DMSO) δ 7.75 (d, 1H), 7.56 (t, 3H), 7.43 (m, 4H), 7.37 – 7.31 (m, 5H), 7.27 (m, 2H), 6.98 (dd, 1H). ^{13}C NMR (101 MHz, DMSO) δ 160.44, 146.54, 136.48, 135.68, 135.16, 131.10, 128.97, 128.39, 127.75, 127.37, 120.99, 120.43, 115.93, 115.64, 115.13, 107.01. ESI-MS: m/z calculated for $C_{26}H_{17}N_2O^+$: 373.1 [M+H$^+$]: not found; decomposes.

4-(2,6-di-tert-butylphenoxy)phthalonitrile (**3f**)

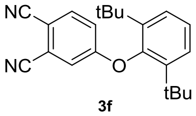

3f

Synthesised from 4-nitrophthalonitrile (150 mg, 0.87 mmol) and 2,6-di*tert*butylphenol (178 mg, 0.87 mmol) in the presence of dry K_2CO_3 (359 mg, 2.6 mmol, 3 eq) and 25 µL DMSO (0.03 µL mg^{-1}) according to the general procedure without pre-treatment. The product did not form (0% conversion. Increasing to 150 µL (0.2 µL mg^{-1}) yielded the product in trace amounts (7% conversion). See crude in Figure S10

4-((2-(dimethylamino)ethyl)thio)phthalonitrile (**3g**)

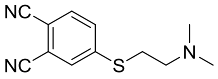

3g

Synthesized from 4-nitrophthalonitrile (200 mg, 1.16 mmol) and 2-(dimethylamino)ethane thiol hydrochloride (122 mg, 1.16 mmol) in the presence of dry K_2CO_3 (479 mg, 3.48 mmol, 3 eq) and 25 µL DMSO (0.03 µL mg^{-1}) according to the general procedure with no pretreatment, affording the crude as a dark brown solid (168 mg, 38% yield) (61% conversion). ^1H NMR (400 MHz, MeOD) δ 7.89 (s, 1H), 7.84 (d, 1H), 7.73 (d, 1H), 3.28 (t, 2H), 2.70 (t, 2H), 2.36 (s, 6H). ^{13}C NMR (101 MHz, MeOD) δ 146.86, 133.30, 130.26, 130.19, 115.83, 115.39, 114.99, 110.51, 57.06, 43.87, 28.62. MS: m/z calculated for $C_{12}H_{14}N_3S^+$ [M+H$^+$]: 232.1; found 232.1

4.3. Scope of phthalonitrile substrates

Table S7: Phthalonitrile substrate scope: Reaction of **1a** with selected mono- and multi-substituted phthalonitriles at varying LAG conditions. All starting materials were pretreated by drying in the vacuum oven and milled for 45 minutes, unless otherwise stated.

Phthalonitrile	Product	LAG (µL mg^{-1})	Conversion (%)	Yield (%)[a]
2a	3a	0.03	96 ± 3	90
2b[b]	3a	0.2	99	73
2c	3a	0.2	0	0
2d	3a	0.2	0	0
2e	3a	0.04	0	0
2f	4	0.03	97 ± 3	85
5a[b]	6	0.15	99	65
5b	9	0.8	99 ± 1	86
7[b]	8	0.2	99	56

[a] The yield was calculated for one experiment based on the ^{1}HNMR conversions as explained in the Supporting Information [b] No pretreatment was done because of decomposition of starting materials.

4-(pyridin-3-yloxy)phthalonitrile (**3a**)

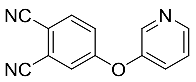
3a

4-fluorophthalonitrile (100 mg, 0.684 mmol), 3-hydroxypyridine (65 mg, 0.684 mmol), dry K_2CO_3 (284 mg, 3 equiv.), and 100 µL DMSO (0.2 µL mg^{-1}) were added to a zirconium jar with a zirconium ball and milled for 45 minutes at 25 Hz. The full batch was dissolved in 40 mL of water and 40 mL of ethyl acetate, after which the aqueous phase was washed 3 x 40 ml EtOAc. The combined organic layers were washed with 100 ml brine, dried over anhydrous $MgSO_4$ and evaporated by rotary evaporator, yielding a crude solid that was analysed without further purification, affording **3a** as a brown solid (110 mg, 73% yield) (99% conversion from ^{1}HNMR) ^{1}H NMR (400 MHz, DMSO): δ 8.53 (m, 2H), 8.12 (d, 1H), 7.88 (d,1H), 7.69 (ddd,1H), 7.53 (ddd,1H), 7.48 (dd,1H). ^{13}C NMR (101 MHz, DMSO): δ 160.91, 151.25, 147.23, 142.74, 136.85, 128.43, 125.65, 123.44, 122.94, 117.31, 116.29, 115.79, 109.39. ESI-MS: m/z calculated for $C_{13}H_8N_3O^+$ [M+H$^+$]: 222.1; found 222.1

3-(pyridin-3-yloxy)phthalonitrile (**4**)

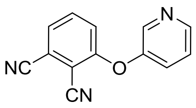
4

Synthesised from 4-nitrophthalonitrile (200 mg, 1.16 mmol) and 3-hydroxypyridine (110 mg, 1.16 mmol) in the presence of K_2CO_3 (479 mg, 3 equiv.) and 25 µL DMSO (0.03 µL mg^{-1}) according to the general procedure, affording **4** as a beige solid (217 mg, 85% yield) (99% conversion from ^{1}HNMR). ^{1}H NMR (400 MHz, DMSO) δ 8.56 (dd, 2H), 7.91 (dt, 2H), 7.75 (ddd, 1H), 7.56 (dd, 1H), 7.39 (d, 1H). ^{13}C NMR (101 MHz, DMSO) δ 159.18, 151.11, 146.88, 141.94, 136.20, 128.84, 127.62, 125.18, 122.38, 116.05, 115.59, 113.26, 105.70. ESI-MS: m/z calculated for $C_{13}H_8N_3O^+$ [M+H$^+$]: 222.1; found 222.1

4,5-bis(pyridin-3-yloxy)phthalonitrile (**7**)

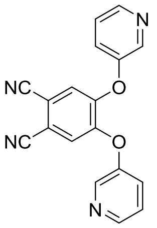
7

4,5-difluorophthalonitrile (100 mg, 0.609 mmol), 3-hydroxypyridine (128 mg, 1.34 mmol), dry K_2CO_3 (421 mg, 5 equiv.), and 130 µL DMSO (0.2 µL mg^{-1}) were added to a zirconium jar with a zirconium ball and milled for 45 minutes at 25 Hz. The full batch was dissolved in 40 mL of water and 40 mL of ethyl acetate, after which the aqueous phase was washed 3 x 40 ml EtOAc. The combined organic layers were washed with 100 ml brine, dried over anhydrous $MgSO_4$ and evaporated by rotary evaporator, yielding a beige solid as a crude that was analysed without further purification, affording **7**. (125 mg, 65% yield) (99% conversion). ^{1}H NMR (400 MHz, DMSO) δ 8.43 (m, 4H), 8.05 (s, 2H), 7.60 (ddd, 2H), 7.46 (ddd, 2H). ^{13}C NMR (101 MHz, DMSO) δ 151.77, 150.32, 145.92, 140.51, 125.91, 125.82, 124.89, 115.20, 111.71. ESI-MS: m/z calculated for $C_{18}H_{11}N_4O_2^+$ [M+H$^+$]: 315.1; found 315.1. m/z calculated for [M+ACN+H]$^+$: 356.1; found 356.1

3,4,5,6-tetrakis(pyridin-3-yloxy)phthalonitrile (**8**)

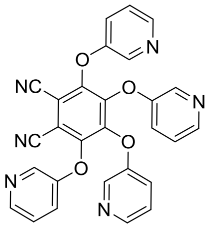
8

Tetrafluorophthalonitrile (100 mg, 0.50 mmol), 3-hydroxypyridine (209 mg, 2.2 mmol), dry K_2CO_3 (421 mg, 10 equiv.), and 130 µL anhydrous DMSO (0.2 µL mg^{-1}) were added to a zirconium jar with a zirconium ball and milled for 45 minutes at 25 Hz. The full batch was dissolved in 40 mL of water and 40 mL of ethyl acetate, after which the aqueous phase was extracted 3 x 40 mL EtOAc. The combined organic layers were washed with 100 ml brine, dried over anhydrous $MgSO_4$ and evaporated by rotary evaporator, affording **8** as a beige solid (169 mg, 67% yield) (99% conversion). ^{1}H NMR (400 MHz, DMSO) δ 8.40 (d, 2H), 8.36 (dd, 2H), 8.23 (t, 2H), 8.02 (s, 2H), 7.59 (dd, 2H), 7.40 (dd, 2H), 7.24 (m, 4H). ^{13}C NMR (101 MHz, DMSO) δ 153.00, 151.91, 148.27, 145.56, 145.32, 145.28, 138.04, 137.54, 124.72, 124.37, 123.38, 122.85, 112.17, 108.75. ESI-MS: m/z calculated for $C_{28}H_{17}N_6O_4^+$ [M+H$^+$]: 501.1; found 501.1

4-chloro-5-(pyridin-3-yloxy)phthalonitrile (**9**)

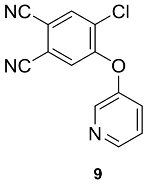
9

Synthesised from 4,5-dichlorophthalonitrile (150 mg, 0.76 mmol) and 3-hydroxypyridine (145 mg, 1.5 mmol, 2 equiv.) in the presence of K_2CO_3 (526 mg, 5 equiv.) and 640 µL DMSO (0.8 µL mg^{-1}) according to the general procedure, extracting with DCM, affording **9** as a white solid (97 mg, 86% yield) (99% conversion from ^{1}HNMR) ^{1}H NMR (400 MHz, DMSO) δ 8.59 (s, 1H), 8.56 – 8.50 (m, 2H), 7.90 (s, 1H), 7.67 (ddd,1H), 7.53 (dd,1H). ^{13}C NMR (101 MHz, DMSO) δ 155.94, 151.12, 146.69, 141.39, 136.30, 129.54, 127.02, 125.21, 123.70, 115.50, 114.92, 114.89, 110.73. ESI-MS: m/z calculated for $C_{13}H_7ClN_3O^+$ [M+H$^+$]: 256.0; found 256.0.

4-([1,1':3',1''-terphenyl]-2'-yloxy)-5-(pyridin-3-yloxy)phthalonitrile (**10**)

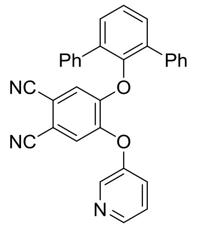

10

Phthalonitrile **9** (75 mg, 0.29 mmol), 2,6-diphenylphenol (87 mg, 0.35 mmol), dry K_2CO_3 (195 mg, 3 equiv.) and 5 mL anhydrous DMF were added to a 25 mL round-bottomed flask and stirred at 80 °C for 24 h. The crude was dissolved in water and extracted with 3 x 40 mL EtOAc. The combined organic layers were washed with 100 mL brine, dried over anhydrous $MgSO_4$ and evaporated by rotary evaporator. The crude was purified by column chromatography (Hex:EtOAc 1:1), yielding a beige solid (20 mg, 15% yield) ^1H NMR (400 MHz, DMSO) δ 8.44 (d,1H), 8.29 (d,1H), 7.70 (s,1H), 7.55 (m, 3H), 7.42 (m, 1H), 7.35 (m, 10H), 7.26 (m, 1H), 7.23 (s,1H). ^{13}C NMR (101 MHz, DMSO) δ 152.19, 151.99, 147.42, 146.37, 145.54, 140.37, 136.36, 134.92, 131.14, 128.79, 128.38, 127.76, 127.34, 125.64, 125.43, 124.81, 120.62, 115.03, 111.19, 108.78. ESI-MS: m/z calculated for $C_{28}H_{20}N_3O_2^+$ [M+H$^+$]: 466.2; found 466.2

4.4. Selected phthalocyanines

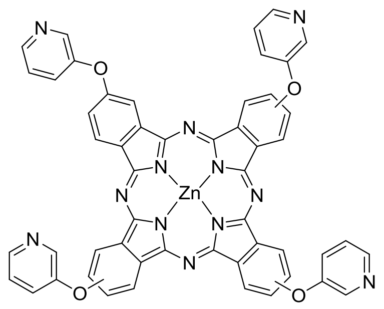

11

[2,(3),9,(10),16,(17),23,(24)-tetrakis(pyridyl-3-oxy)phthalocyaninato]zinc(II) (**11**)

Phthalonitrile **3a** (200 mg, 0.90 mmol), $Zn(OAc)_2$ (83 mg, 0.45 mmol), and 2-dimethylaminoethanol (DMAE) (50 µL, 0.18 µL mg^{-1}) were placed in a stainless-steel jar with a single stainless-steel ball. The mixture was milled for 5 minutes at 30 Hz. The resulting paste-like homogenized mixture was placed in the oven at 120 °C for 48 h. The mixture changed into deep blue within 10 minutes. The crude was washed with water and hot methanol, after which it was analysed without further purification affording **11** as a deep blue solid (138 mg, 65% yield). MALDI-TOF: m/z calculated for $C_{52}H_{28}N_{12}O_4Zn$: 948.16; found 948.09.

[1,8(11),15(18),22(25) -tetrakis(pyridyl-3-oxy)phthalocyaninato]zinc(II) (**12**)

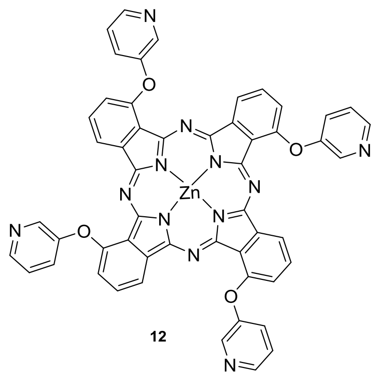

12

Phthalonitrile **4** (110 mg, 0.50 mmol), $Zn(OAc)_2$ (53 mg, 0.29 mmol), and DMAE (25 µL, 0.15 µL mg^{-1}) were placed in a stainless steel jar with a single stainless steel ball. The mixture was milled for 5 minutes at 30 Hz. The resulting paste-like homogenized mixture was placed in the oven at 120 °C for 48 h. The mixture changed into deep blue within 10 minutes. The crude was washed with water and hot methanol, after which it was analysed without further purification affording **12** as a deep blue solid (70 mg, 59% yield). MALDI-TOF: m/z calculated for $C_{52}H_{28}N_{12}O_4Zn$: 948.16; found 948.23.

[2,3,9,10,16,17,23,24-octakis(pyridyl-3-oxy) phthalocyaninato]zinc(II) (**13**)

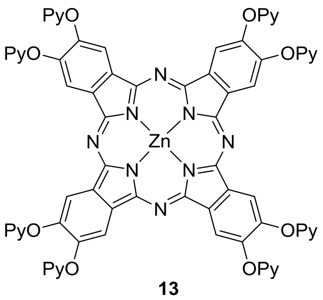

13

Phthalonitrile **6** (120 mg, 0.38 mmol), $Zn(OAc)_2$ (25 mg, 0.14 mmol), and DMAE (30 µL, 0.2 µL mg^{-1}) were placed in a stainless steel jar with a single stainless steel ball. The mixture was milled for 5 minutes at 30 Hz. The resulting paste-like homogenized mixture was placed in the oven at 120 °C for 48 h. The mixture changed into deep blue within 10 minutes. The crude was washed with water and hot methanol, after which it was analysed without further purification affording **13** as a dark green solid (67 mg, 53% yield). MALDI-TOF: m/z calculated for $C_{72}H_{40}N_{16}O_8Zn$: 1320.25; found 1320.77

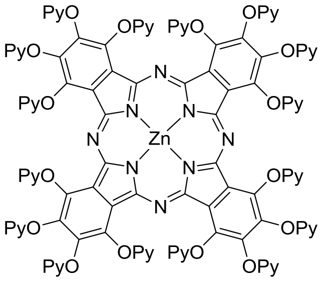

14

[1,2,3,4,8,9,10,11,15,16,17,18,22,23,24,25-hexadecakis(pyridyl-3-oxy) phthalocyaninato]zinc(II) (**14**)

Phthalonitrile **7** (75 mg, 0.15 mmol), $Zn(OAc)_2$ (10 mg, 0.05 mmol), and DMAE (17 µL, 0.2 µL mg^{-1}) were placed in a stainless steel jar with a single stainless steel ball. The mixture was milled for 5 minutes at 30 Hz. The resulting paste-like homogenized mixture was placed in the oven at 120 °C for 48 h. The mixture changed into deep blue within 10 minutes. The crude was washed with water and hot methanol, after which it was analysed without further purification affording **14** as a dark green solid (77 mg, 98% yield). MALDI-TOF: m/z calculated for $C_{112}H_{64}N_{24}O_{16}Zn$: 2064.42; found 2064.72.

4.5. One-pot synthesis of phthaocyanines

2,(3),9,(10),16,(17),23,(24)-tetra(pyridyl-3-oxy)phthalocyanine (**15**)

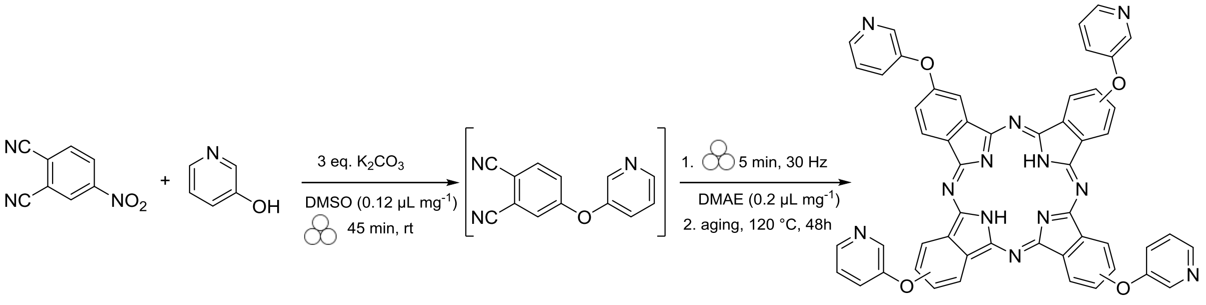

15

4-nitrophthalonitrile (200 mg, 1.16 mmol) and 3-hydroxypyiridine (110 mg, 1.16 mmol) were pretreated by placing in a small vial and kept in the vacuum oven at 40 °C for 1 hour. The solid mixture was added to a stainless steel milling jar containing a single stainless steel ball with anhydrous K_2CO_3 (480 mg, 3 equiv.) and 100 µL dry DMSO (0.12 µL mg^{-1}). The mixture was milled for 45 minutes at 25 Hz. The conversion was verified by TLC, affording complete conversion to phthalonitrile **3a**. To the same milling jar was added 160 µL DMAE (0.2 µL mg^{-1}) and the mixture was milled for 5 minutes at 30 Hz. The resulting paste-like homogenized mixture was placed in the oven at 120 °C for 48 h. The crude was washed with water and hot methanol, after which it was analysed without further purification affording **15** metal-free phthalocyanine as a deep blue-green solid (180 mg, 70% yield). MALDI-TOF: m/z calculated for $C_{52}H_{30}N_{12}O_4$: 886.25; found 886.18.

[2,(3),9,(10),16,(17),23,(24)-tetrakis(pyridyl-3-oxy)phthalocyaninato]zinc(II) (**11**)

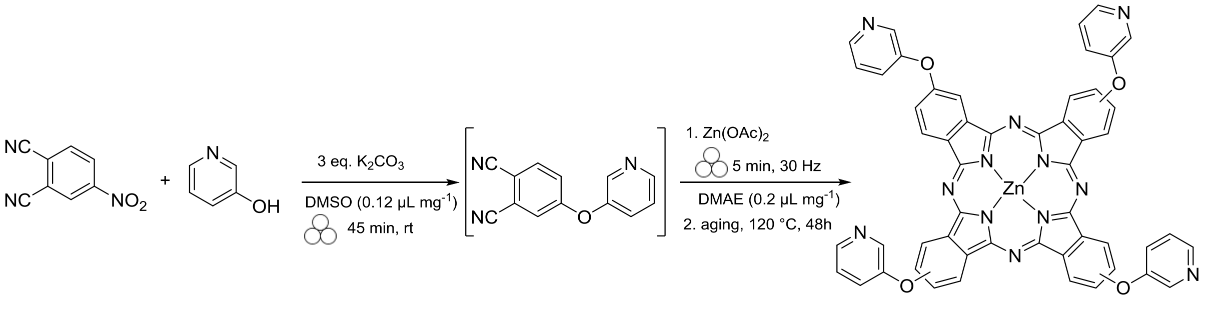

11

4-nitrophthalonitrile (200 mg, 1.16 mmol) and 3-hydroxypyiridine (110 mg, 1.16 mmol) were pretreated by placing in a small vial and kept in the vacuum oven at 40 °C for 1 hour. The solid mixture was added to a stainless steel milling jar containing a single stainless steel ball with anhydrous K_2CO_3 (480 mg, 3 equiv.) and 100 µL dry DMSO (0.12 µL mg^{-1}). The mixture was milled for 45 minutes at 25 Hz. The conversion was verified by TLC, affording complete conversion to phthalonitrile **3a**. To the same milling jar was added $Zn(OAc)_2$ (423 mg, 8 equiv. with respect to Pc) and 250 µL DMAE (0.2 µL mg^{-1}). The mixture was milled for 5 minutes at 30 Hz. The resulting paste-like homogenized mixture was placed in the oven at 120 °C for 48 h. The crude was washed with water and hot methanol, after which it was analysed without further purification affording **11** zinc phthalocyanine as a blue-green solid (151 mg, 55% yield). ^1H NMR (400 MHz, DMSO) δ 8.47 (dd, 8H), 7.60 (d, 8H), 7.49 (dd, 4H), 7.25 (d, 4H), 7.07 (s, 4H). MALDI-TOF: m/z calculated for $C_{52}H_{30}N_{12}O_4$: 948.16; found 948.10.

5. LAG influence on keto-enol tautomerization of 4-hydroxypyridine

Table S8: Reaction conditions of synthesis of **3b** with varying amount of solvent with **1b** and **2a** in 1:1 ratio and 3 equiv. of K_2CO_3.

V (µL)	LAG (µL/mg)	2a (%)	Conv.to 3b-keto (%)	Conv. to 3b-enol (%)
0	0	93	7	0
25	0.03	5	91	4
150	0.2	18	57	25
400	0.5	17	59	24
10 mL[a]	solution	0	54	46

[a] Reaction performed with 10 mL of DMF and heating at 80 °C for 16 hours

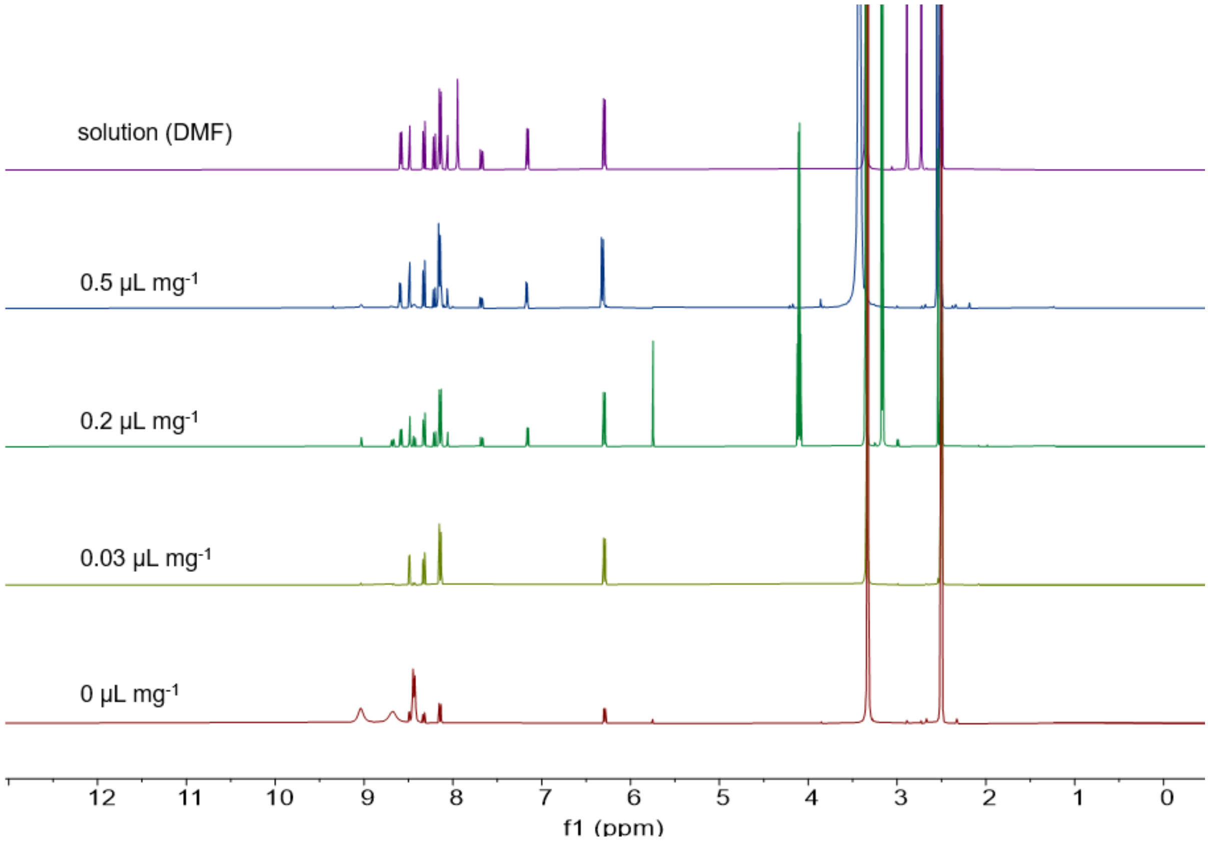

Figure S6: ^1H spectra for the synthesis of **3b**, showing the reaction outcome in dependence of η. Experimental details are shown in **Table S8.**

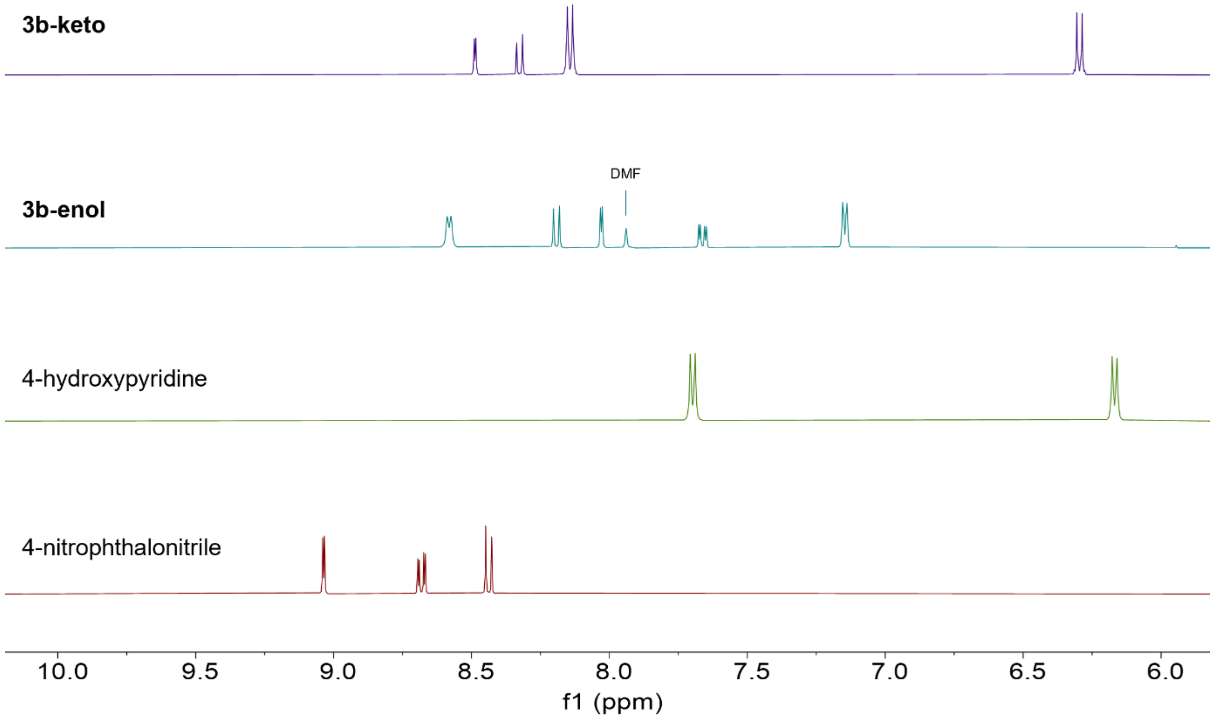

Figure S7: ^{1}H spectra comparison of the 4-nitrophthalonitrile (**2a**), 4-hydroxypyridine (**1b**), **3b-enol**, and **3b-keto** in the aromatic region**.**

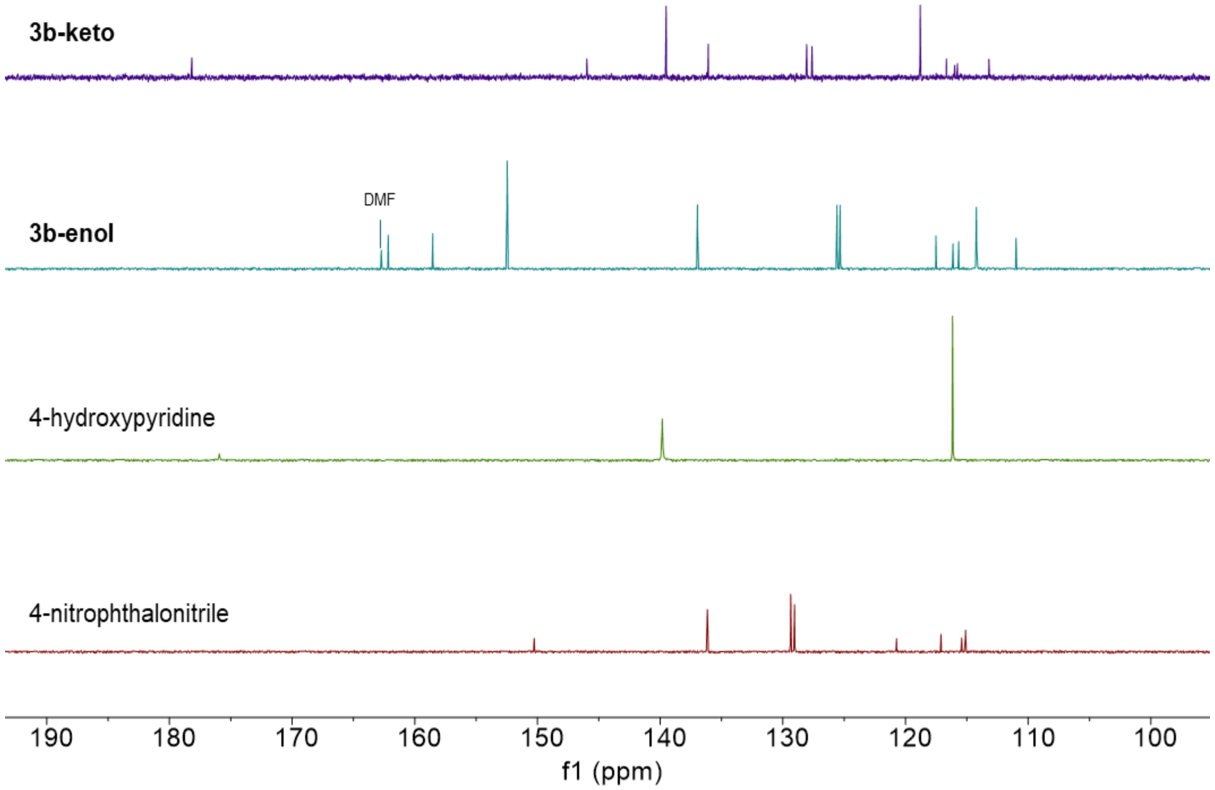

Figure S8: ^{13}C spectra comparison of the 4-nitrophthalonitrile (**2a**), 4-hydroxypyridine (**1b**), **3b-enol**, and **3b-keto** in the aromatic region**.**

6. LAG influence on keto-enol tautomerization of 2-hydroxypyridine

Table S9: Reaction conditions of synthesis of **3c** with varying amount of solvent with **1c** and **2a** in 1:1 ratio and 3 equiv. of K_2CO_3.

V (µL)	LAG (µL/mg)	2a (%)	Conv.to 3c-keto (%)	Conv. to 3c-enol (%)
25	0.03	18	79	3
150	0.2	1	90	9

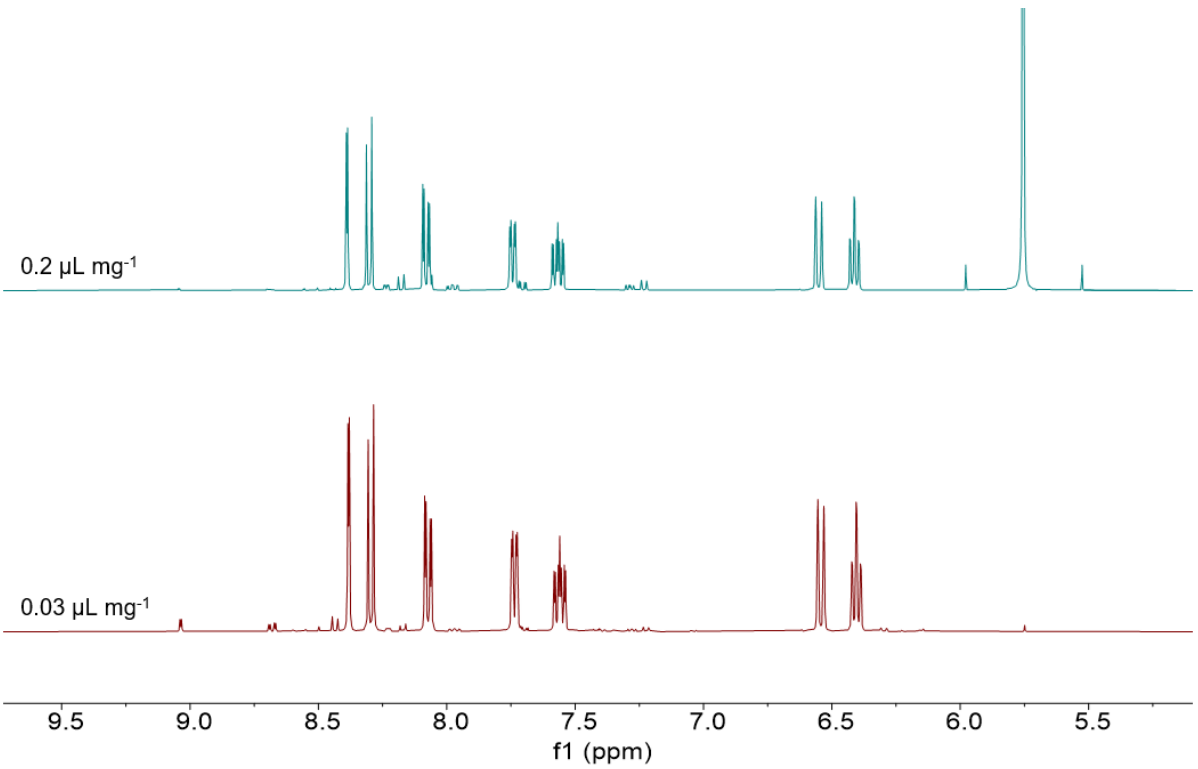

Figure S9: ^{1}H spectra for the synthesis of **3c**, showing the reaction outcome in dependence of η in the aromatic region. Experimental details are shown in **Table S9.**

7. Unsuccesful synthesis of 3f

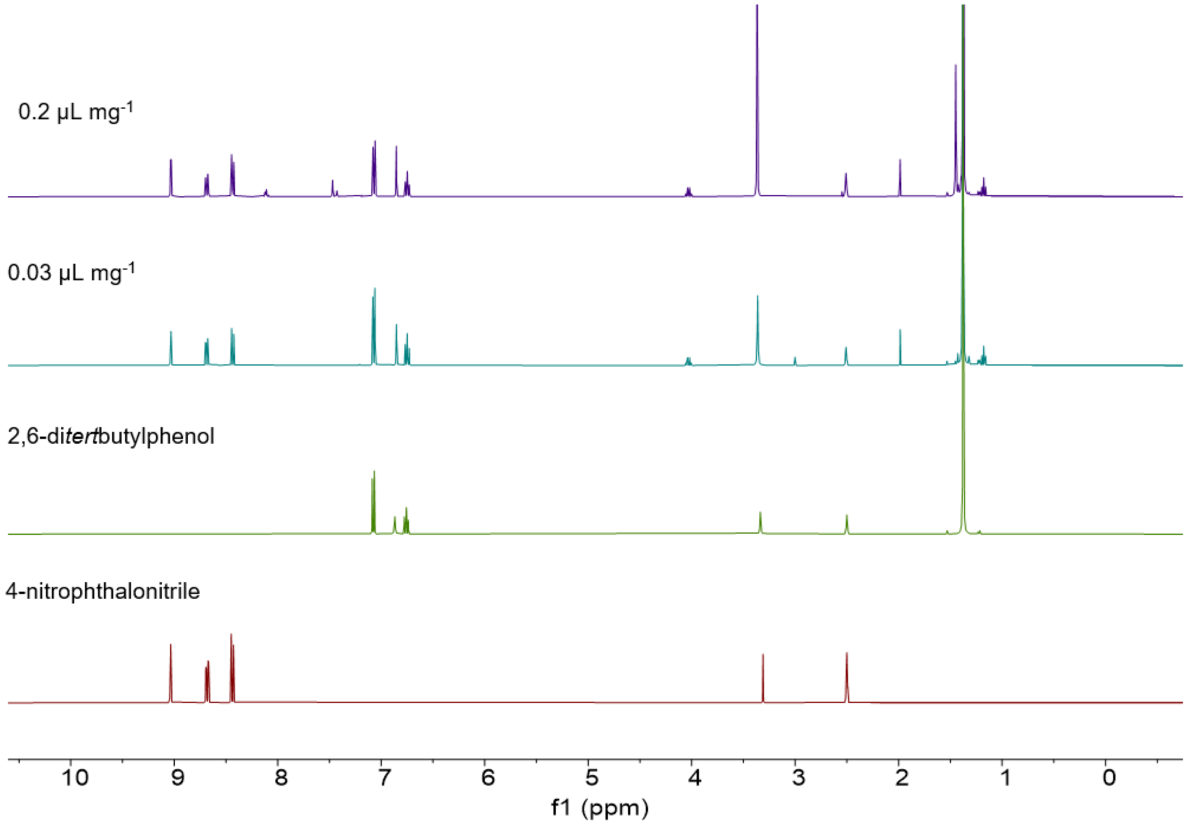

Figure S10: ^{1}H spectra for the synthesis of **3f**, showing the reaction outcome in dependence of η. No conversion occurs at 0.03 µL mg^{-1}, while only traces are visible at 0.2 µL mg^{-1}.

8. Optimization of the synthesis of 9

Table S10: Reaction conditions of synthesis of **9** varying the amount of solvent with **1a** in 2 equiv. with respect to **5b** and 5 equiv. of K_2CO_3.

V (µL)	LAG (µL/mg)	Conversion to **9** (%)
25	0.04	6
150	0.08	24
500	0.8	99

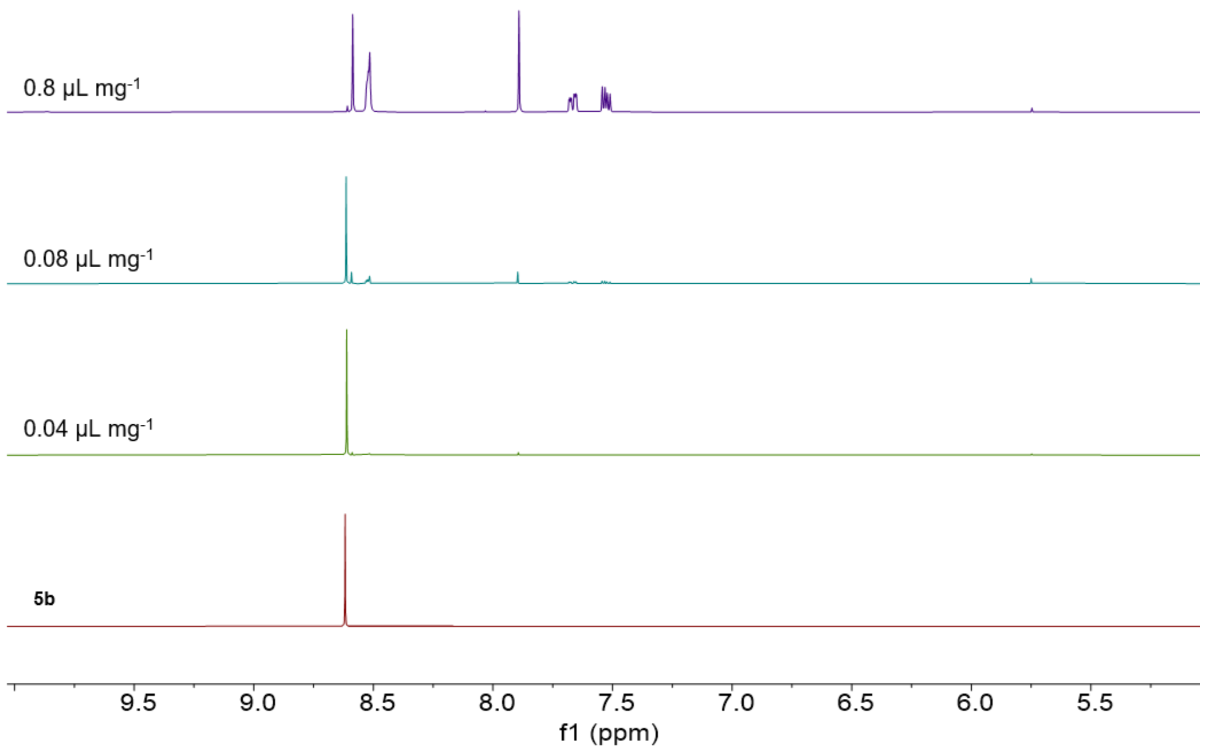

Figure S11: ^{1}H spectra for the synthesis of **9**, showing the reaction outcome in dependence of η in the aromatic region. Experimental details are shown in **Table S10.**

9. Influence of potassium carbonate and zinc acetate in the synthesis of 11 and 15

Pure phthalonitrile **3a** (200 mg, 0.90 mmol) and 2-dimethylaminoethanol (DMAE) (40 µL, 0.2 µL mg^{-1}) were placed in a stainless steel jar with a single stainless steel ball. The mixture was milled for 5 minutes at 30 Hz. The resulting paste-like homogenized mixture was placed in the oven at 120 °C for 48 h. The color change was minimal. The crude was washed with water and hot methanol, after which it was analysed without further purification, yielding no conversion as shown in the ^{1}H spectrum.

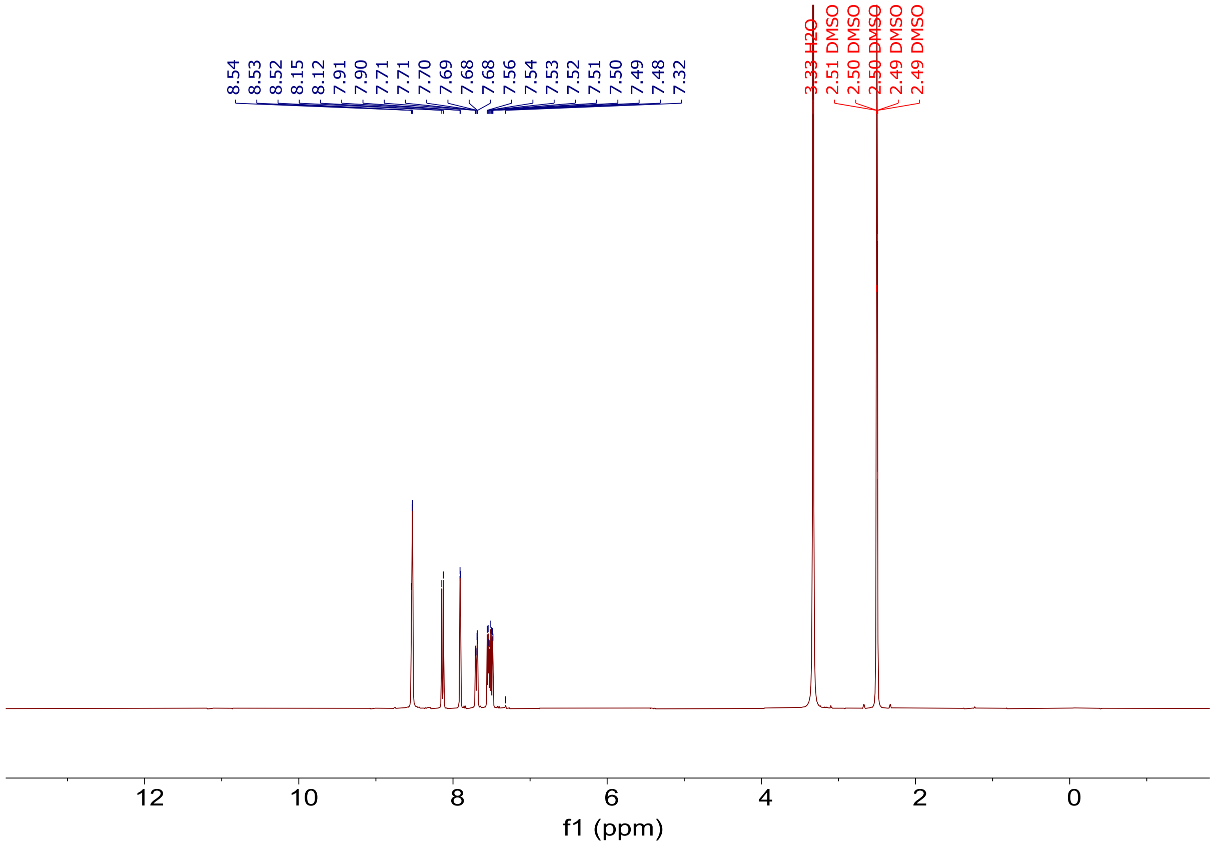

Figure S12: 1H spectrum for the synthesis of **11** without zinc acetate. Only the starting material is present, showing the need of zinc as a templating agent, or a base for the formation of **15**.

Pure phthalonitrile **3a** (125 mg, 0.72 mmol), potassium chloride (45 mg, 0,60) and 2-dimethylaminoethanol (DMAE) (35 µL, 0.2 µL mg^{-1}) were placed in a stainless steel jar with a single stainless steel ball. The mixture was milled for 5 minutes at 30 Hz. The resulting paste-like homogenized mixture was placed in the oven at 120 °C for 48 h. The color change was minimal. The crude was washed with water and hot methanol, after which it was analysed without further purification, yielding no conversion as shown in the ^{1}H spectrum.

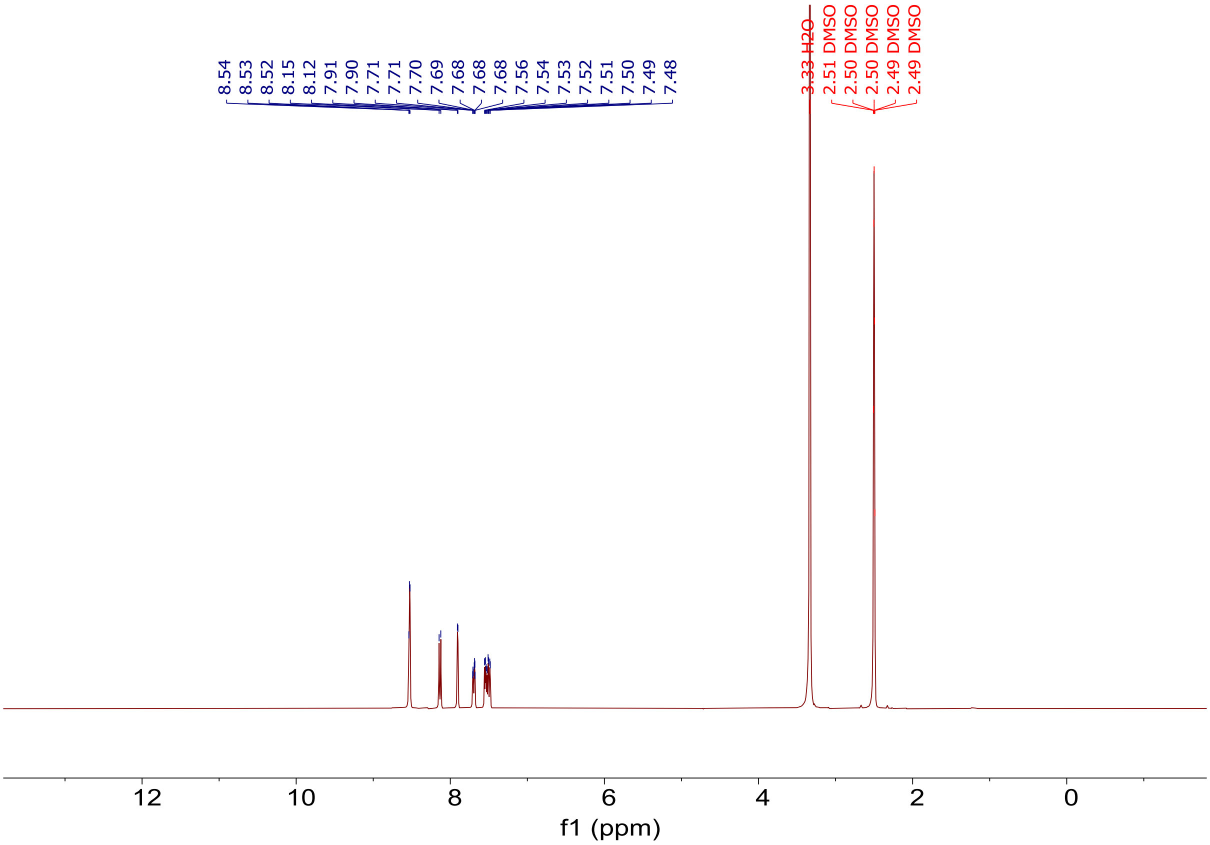

Figure 13: 1H spectrum for the synthesis of **15** with potassium chloride as a templating agent. Only the starting material is present, showing the need of a base to ensure the tetramerization into the metal free.

4-nitrophthalonitrile (200 mg, 1.16 mmol) and 3-hydroxypyiridine (110 mg, 1.16 mmol) were pretreated by placing in a small vial and kept in the vacuum oven at 40 °C for 1 hour. The solid mixture was added to a stainless steel milling jar containing a single stainless steel ball with anhydrous K_2CO_3 (480 mg, 3 equiv.) and 100 µL dry DMSO (0.12 µL mg^{-1}). The mixture was milled for 45 minutes at 25 Hz. The conversion was verified by TLC, affording complete conversion to phthalonitrile **3a**. To the same milling jar was added added $Zn(OAc)_2$ (75 mg, 0.5 equiv.) and 160 µL DMAE (0.2 µL mg^{-1}) and the mixture was milled for 5 minutes at 30 Hz. The resulting paste-like homogenized mixture was placed in the oven at 120 °C for 48 h. The crude was washed with water and hot methanol, after which it was analysed without further purification affording a mixture of **11** and **15** phthalocyanines as a deep blue-green solid.

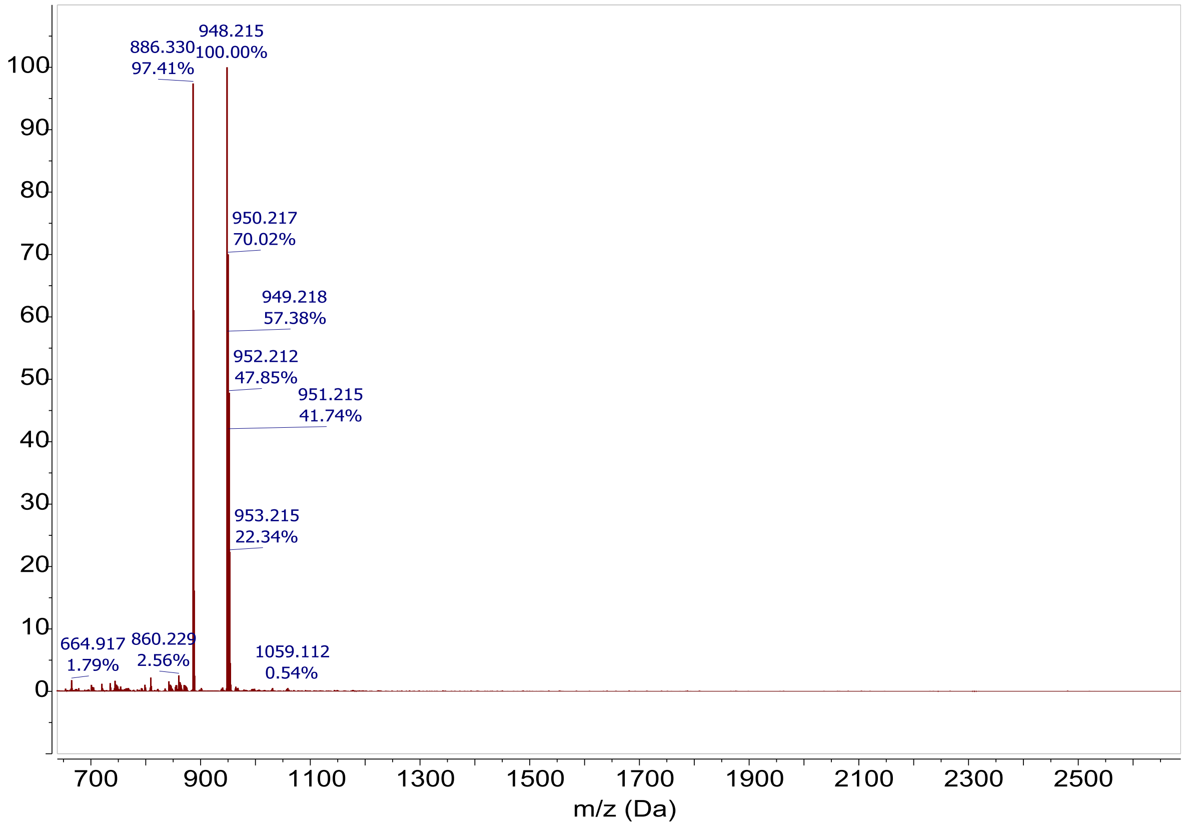

Figure S14: MALDI spectrum for the one pot synthesis of **11** with 4 equivalents of zinc acetate as a templating agent relative to the phthalocyanine. The peaks of **11** (948.215), and **15** (886.330) are visible.

10. E-factor calculations

The E-factor (environmental factor) is a tool used to assess the environmental impact of a chemical process and is defined as the ratio of the total mass of waste generated to the mass of the isolated product. The general equation for its calculation is:

$$E - factor = \frac{Mass\ of\ total\ waste\ (g)}{Mass\ of\ product\ (g)}$$

Water has been excluded from the waste count, consistent with standard practices that do not consider water a harmful by-product.[1] We have included calculations excluding the work up as well.

Calculation for mechanochemical S_NAr of 3a (excl. water)

Table S11: Used chemicals and their masses for the E-Factor calculation of the benchmark S_NAr reaction.

Chemical	Volume (mL)	Density (g/mL)	Mass (g)
4-nitrophthalonitrile			0.200
3-hydroxypyridine			0.110
K_2CO_3			0.480
DMSO	0.025	1.1	0.0275
EtOAC	120	0.902	108
$MgSO_4$			0.500
Product **3a**			0.230

E-factor = 474.3

Calculation for mechanochemical S_NAr of 3a (excl. work-up)

Table S12: Used chemicals and their masses for the E-Factor calculation of the benchmark S_NAr reaction excluding workup.

Chemical	Volume (mL)	Density (g/mL)	Mass (g)
4-nitrophthalonitrile			0.200
3-hydroxypyridine			0.110
K_2CO_3			0.480
DMSO	0.025	1.1	0.0275
Product **3a**			0.230

E-factor = 2.55

Calculation for tetramerization step of 3a into zinc phthalocyanine 11 by aging (excl. water)

Table S13: Used chemicals and their masses for the E-Factor calculation of the tetramerization step of **3a** by aging.

Chemical	Volume (mL)	Density (g/mL)	Mass (g)
Phthalonitrile **3a**			0.200
Zinc acetate			0.083
DMAE	0.050	0.890	0.045
Methanol	10	0.792	7.92
Phthalocyanine **11**			0.138

E-factor = 58.8

Calculation for tetramerization step of 3a into zinc phthalocyanine 11 by aging (excl. workup)

Table S14: Used chemicals and their masses for the E-Factor calculation of the tetramerization step of **3a** by aging excluding workup.

Chemical	Volume (mL)	Density (g/mL)	Mass (g)
Phthalonitrile **3a**			0.200
Zinc acetate			0.083
DMAE	0.050	0.890	0.045
Phthalocyanine **11**			0.138

E-factor = 1.38

Calculation for one pot synthesis of zinc phthalocyanine 11 (excl. water)

Table S15: Used chemicals and their masses for the E-Factor calculation of the one pot synthesis of **11** by mechanochemistry and aging.

Chemical	Volume (mL)	Density (g/mL)	Mass (g)
4-nitrophthalonitrile			0.200
3-hydroxypyridine			0.110
K_2CO_3			0.480
DMSO	0.100	1.1	0.110
Zinc acetate			0.423
DMAE	0.250	0.890	0.222
Methanol	10	0.792	7.92
Phthalocyanine **11**			0.151

E-factor = 61.7

Calculation for one pot synthesis of zinc phthalocyanine 11 (excl. workup)

Table S16: Used chemicals and their masses for the E-Factor calculation of the one pot synthesis of **11** by mechanochemistry and aging excluding workup.

Chemical	Volume (mL)	Density (g/mL)	Mass (g)
4-nitrophthalonitrile			0.200
3-hydroxypyridine			0.110
K_2CO_3			0.480
DMSO	0.100	1.1	0.110
Zinc acetate			0.423
DMAE	0.250	0.890	0.222
Phthalocyanine **11**			0.151

E-factor = 9.23

Calculation for one pot synthesis of metal-free phthalocyanine 15 (excl. water)

Table S17: Used chemicals and their masses for the E-Factor calculation of the one pot synthesis of **15** by mechanochemistry and aging.

Chemical	Volume (mL)	Density (g/mL)	Mass (g)
4-nitrophthalonitrile			0.200
3-hydroxypyridine			0.110
K_2CO_3			0.480
DMSO	0.100	1.1	0.110
DMAE	0.160	0.890	0.142
Methanol	10	0.792	7.92
Phthalocyanine **15**			0.151

E-factor = 48.8

Calculation for one pot synthesis of metal-free phthalocyanine 15 (excl. workup)

Table S18: Used chemicals and their masses for the E-Factor calculation of the one pot synthesis of **15** by mechanochemistry and aging excluding workup.

Chemical	Volume (mL)	Density (g/mL)	Mass (g)
4-nitrophthalonitrile			0.200
3-hydroxypyridine			0.110
K_2CO_3			0.480
DMSO	0.100	1.1	0.110
DMAE	0.160	0.890	0.142
Phthalocyanine **15**			0.151

E-factor = 5.90

11. Structural characterization spectra

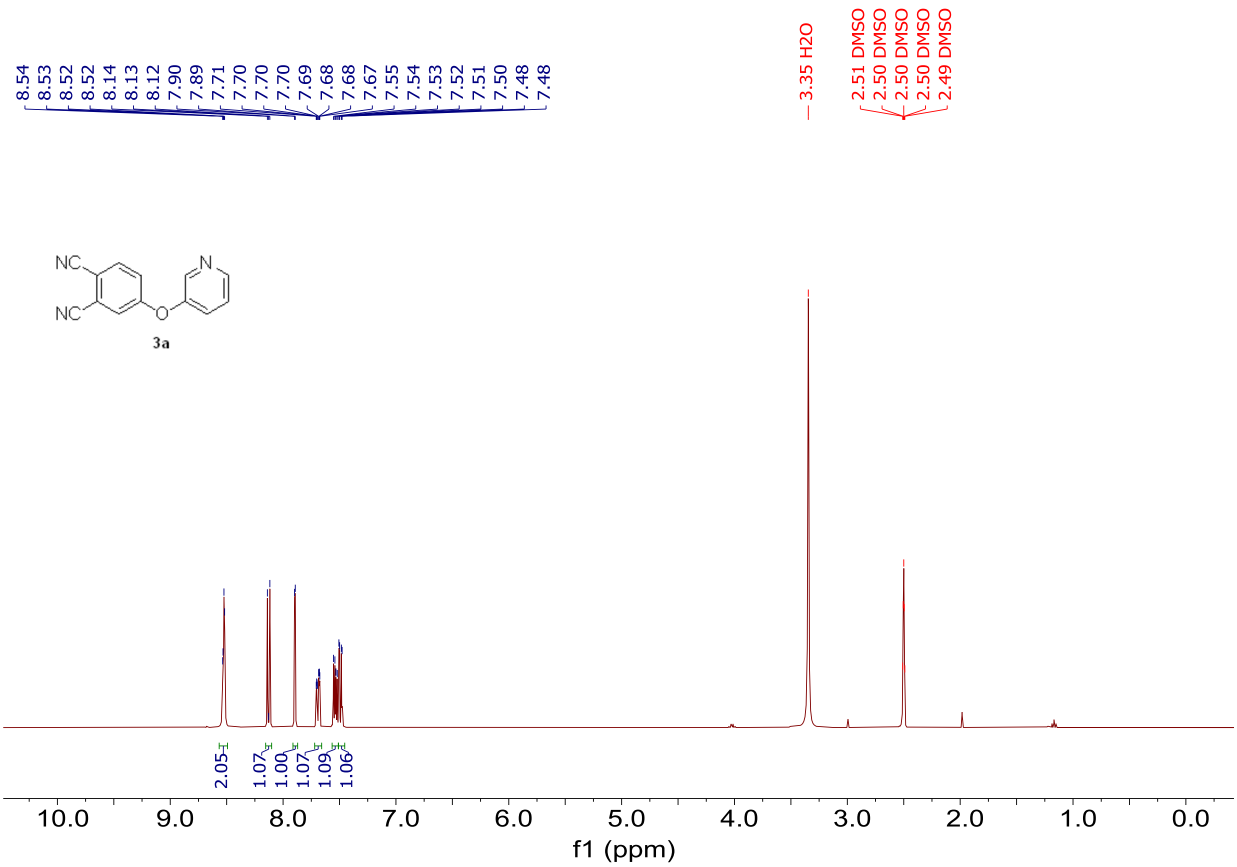

Figure S15: ^{1}H spectrum of **3a**.

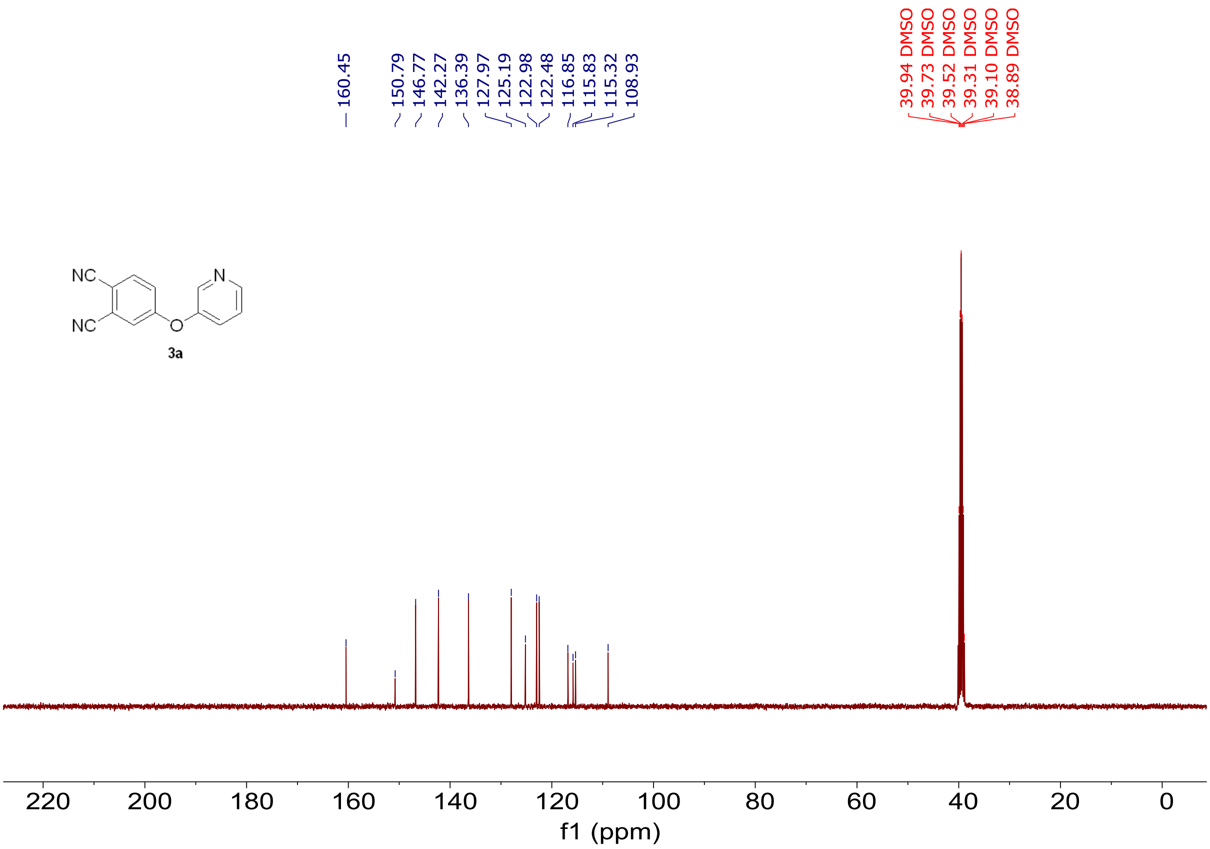

Figure S16: ^{13}C spectrum of **3a**.

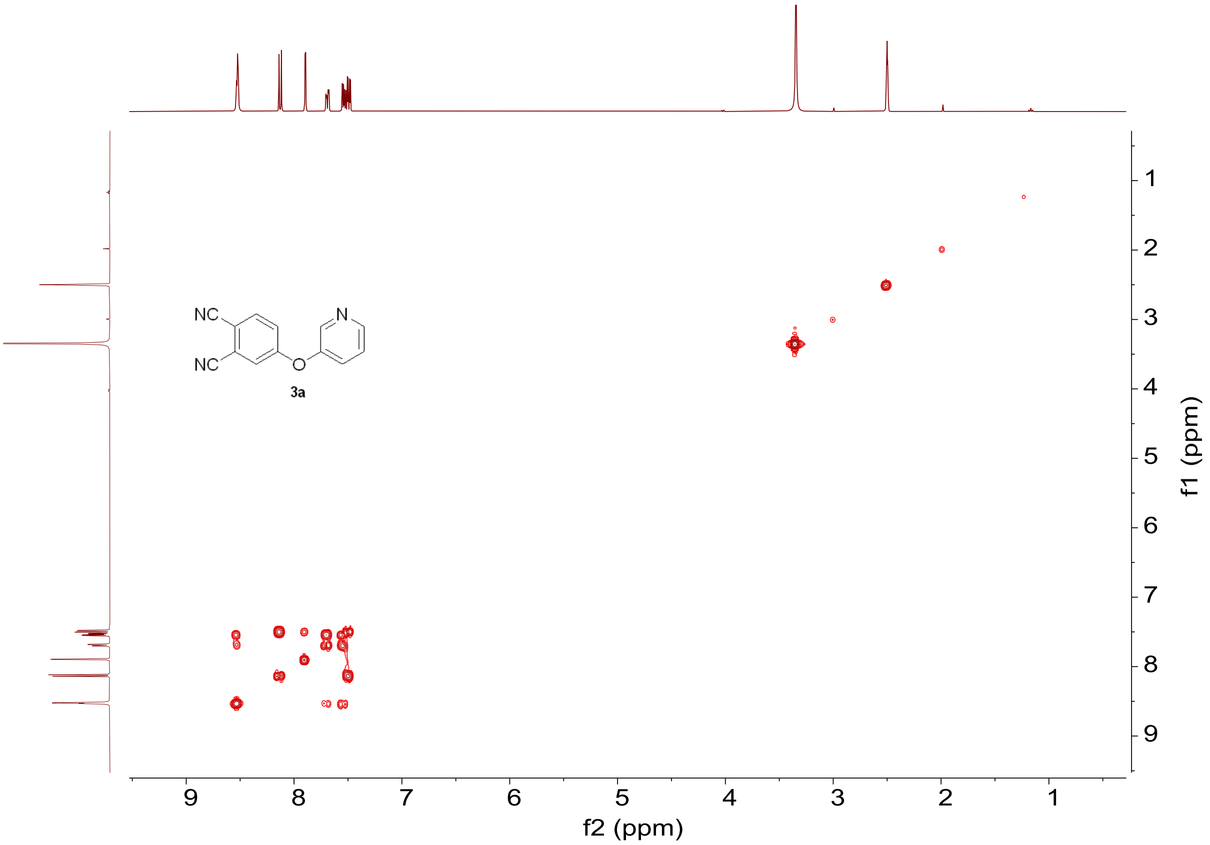

Figure S17: COSY spectrum of **3a**.

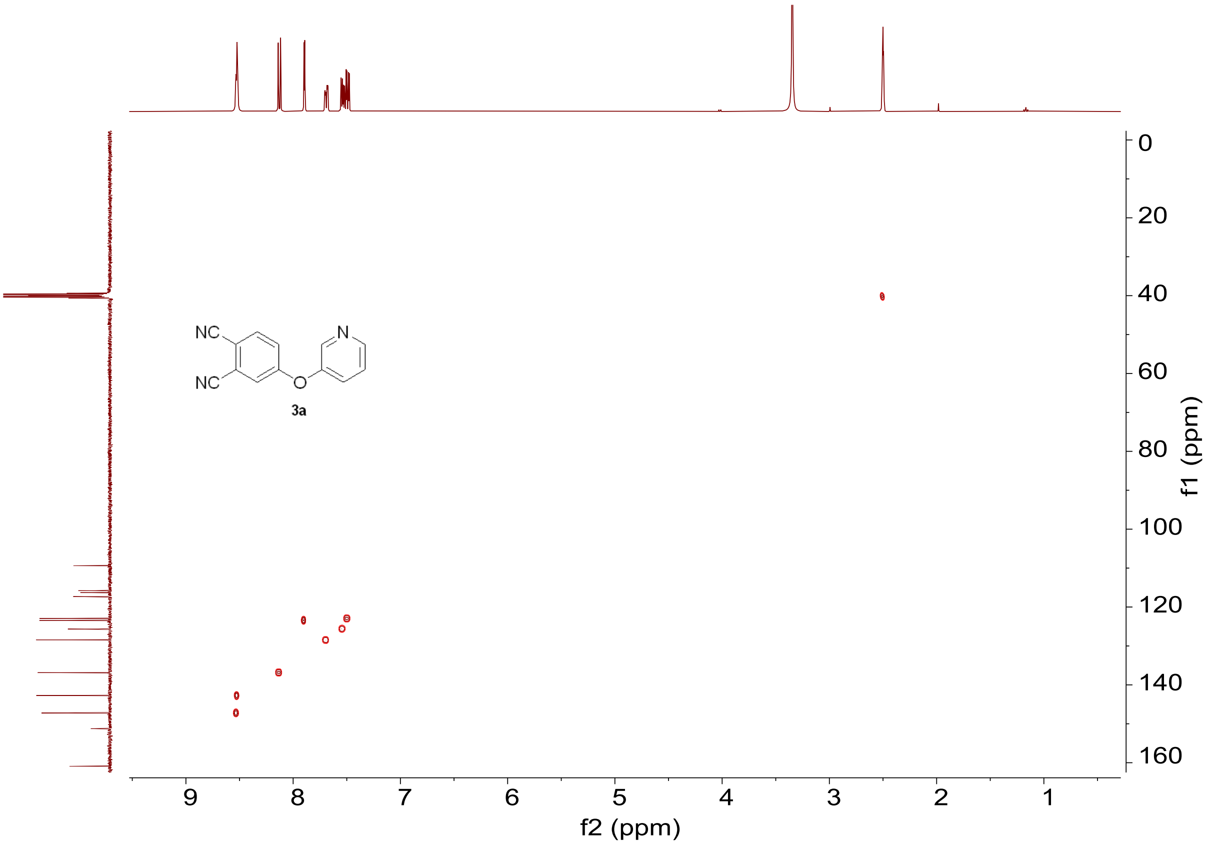

Figure S18: HSQC spectrum of **3a**.

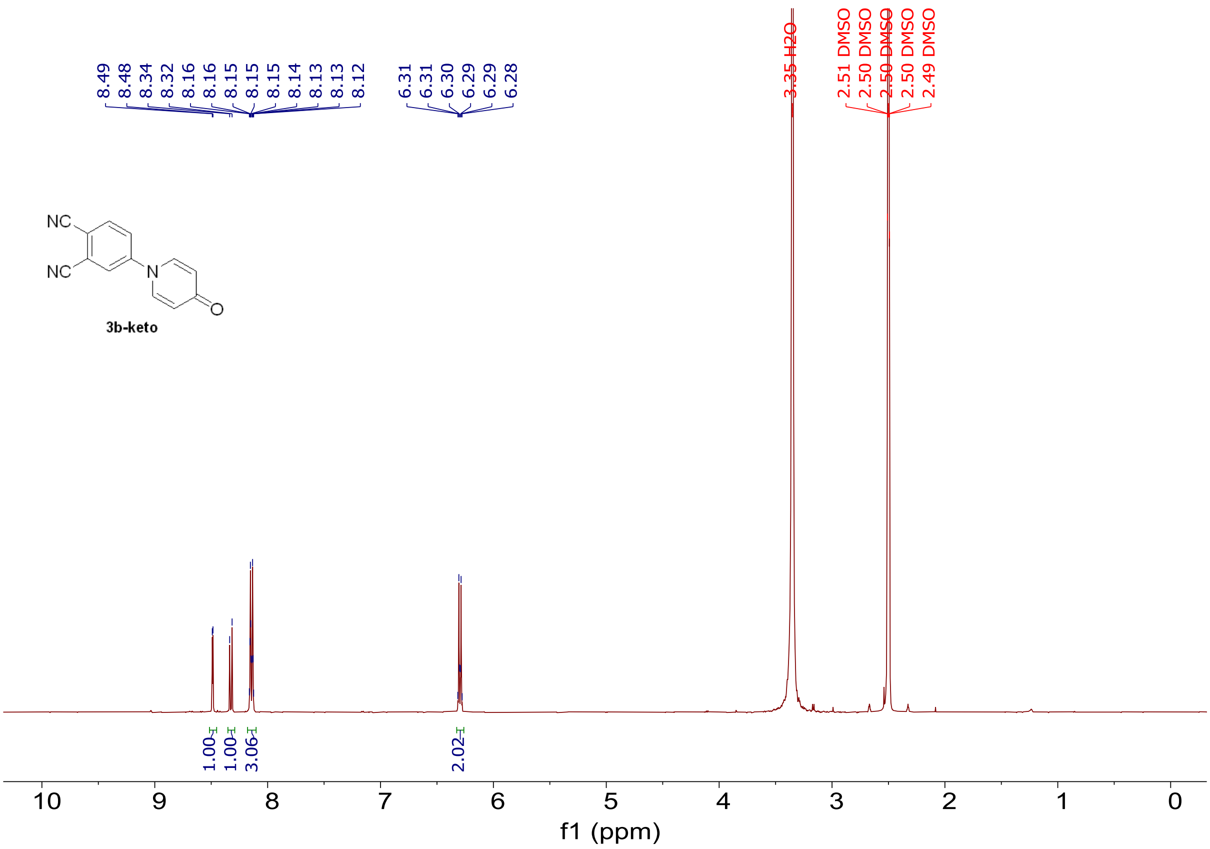

Figure S19: ^{1}H spectrum of **3b-*keto***.

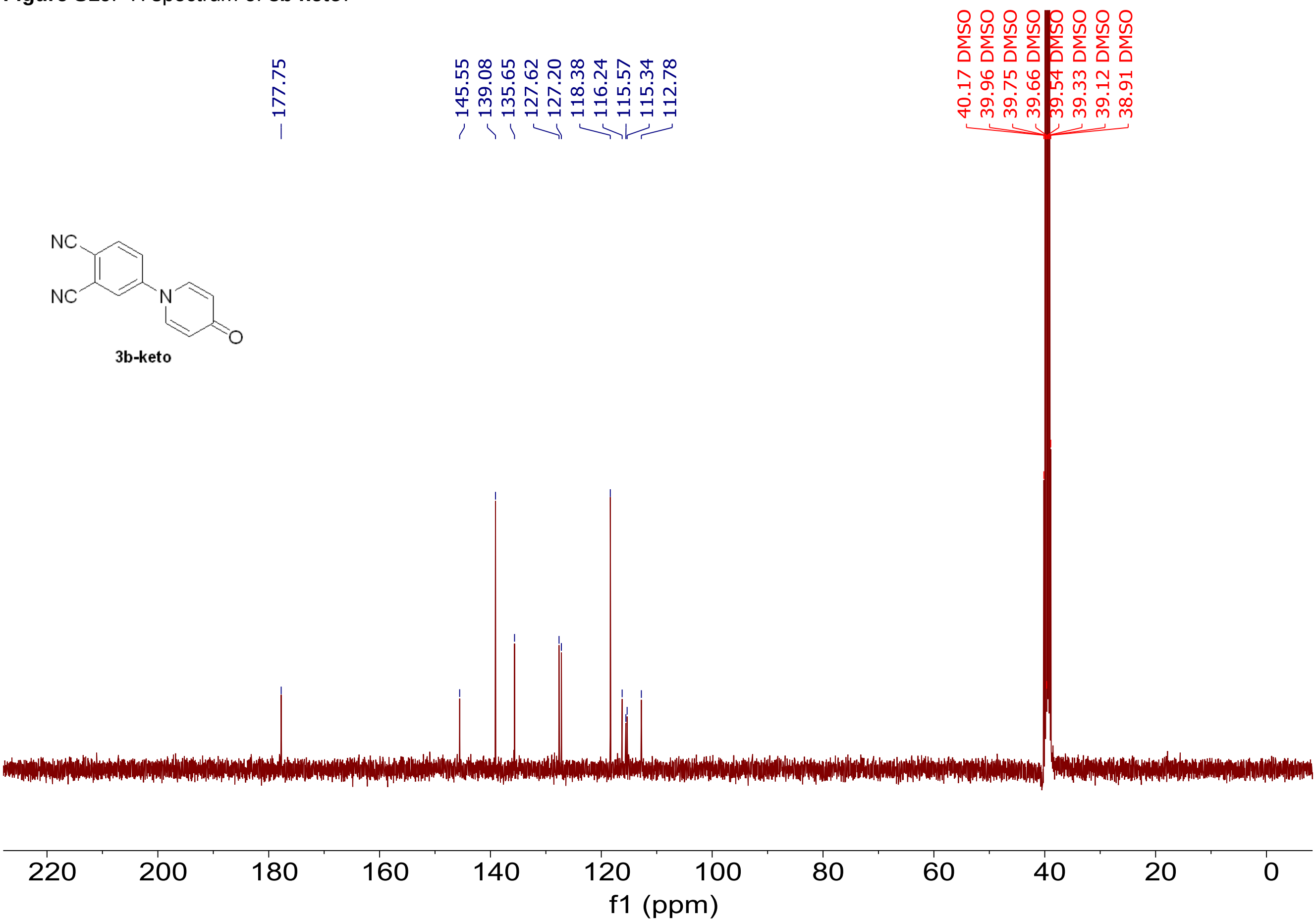

Figure S20: ^{13}C spectrum of **3b-*keto***.

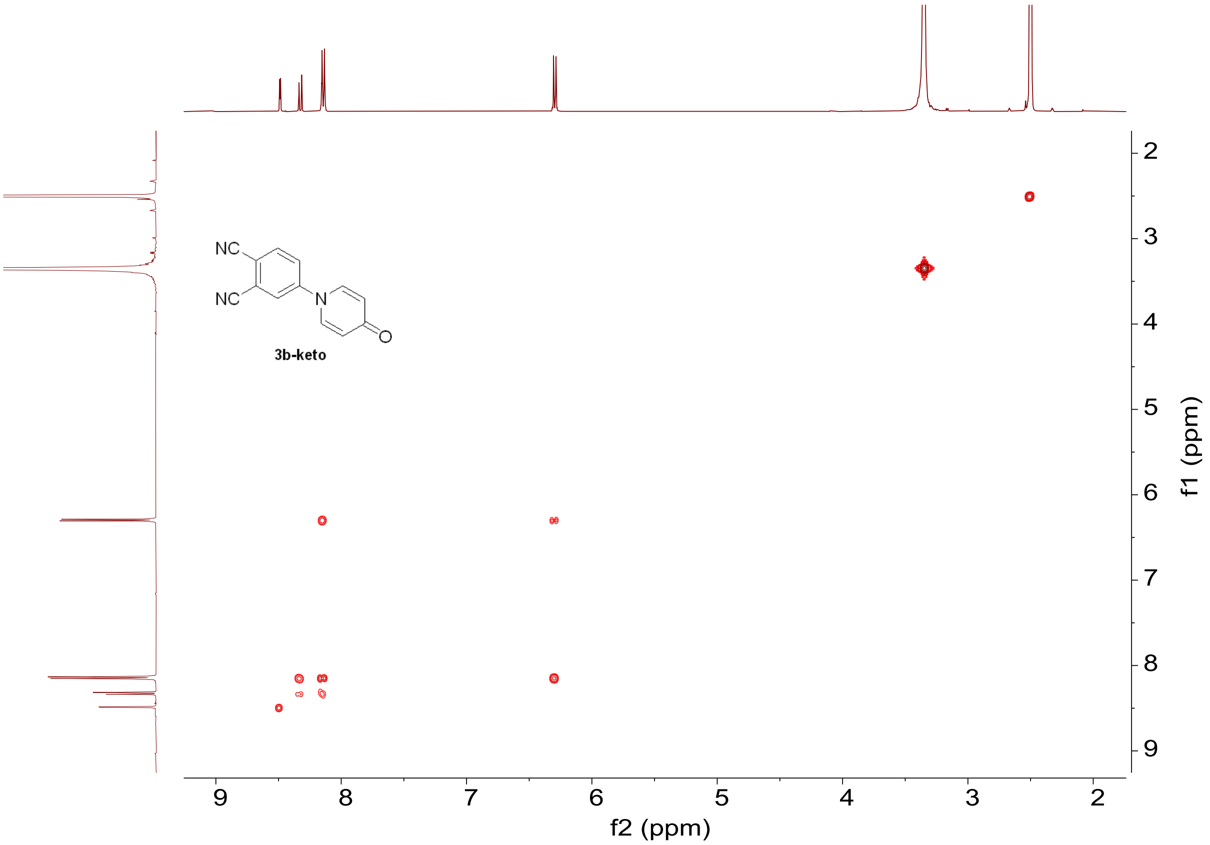

Figure S21: COSY spectrum of **3b-*keto***.

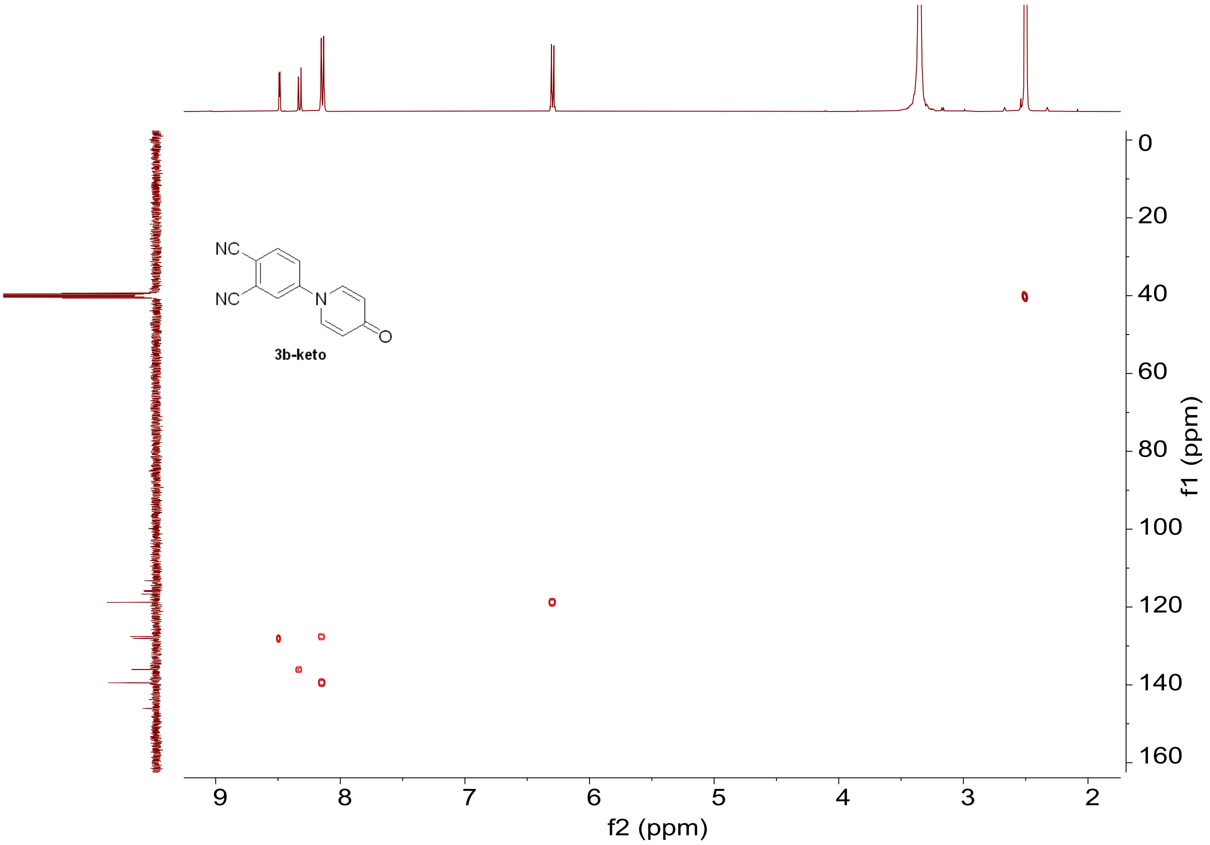

Figure S22: HSQC spectrum of **3b-*keto***.

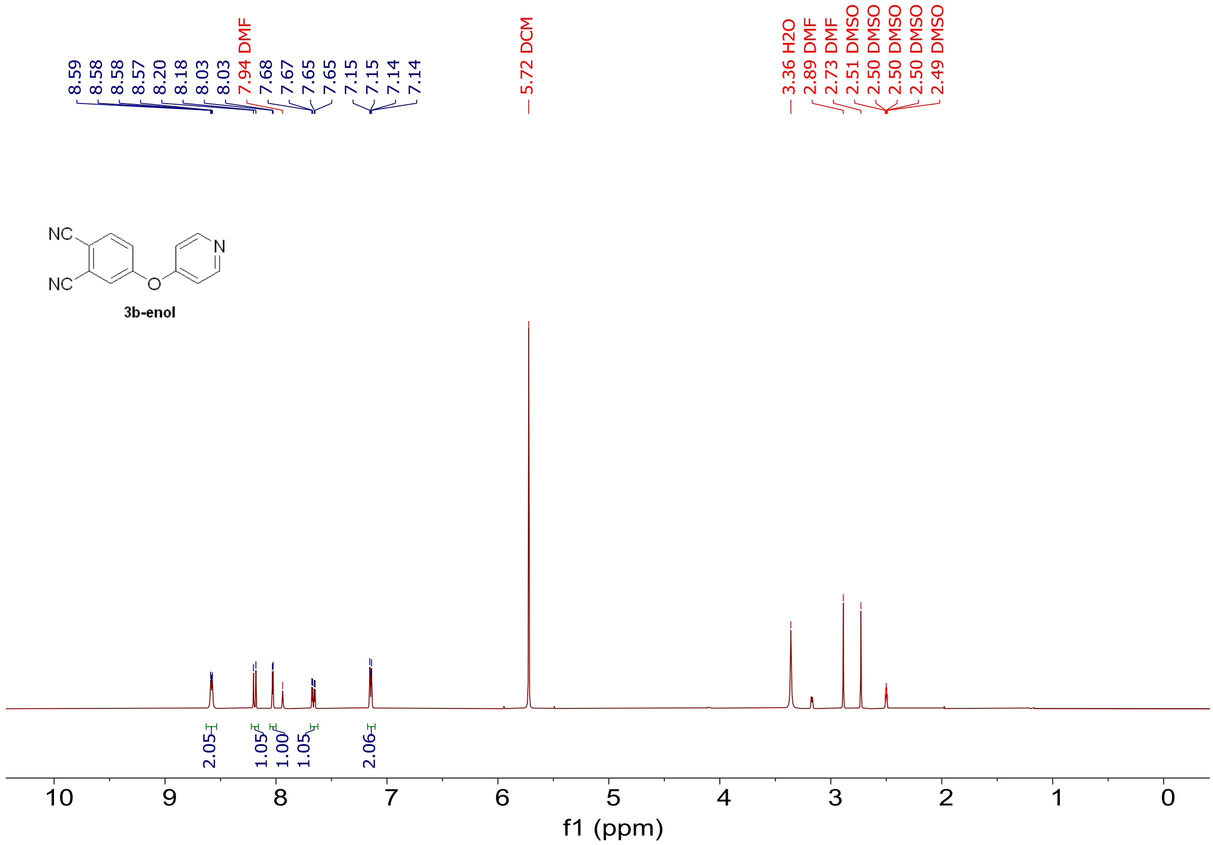

Figure S23: ^{1}H spectrum of **3b-*enol***.

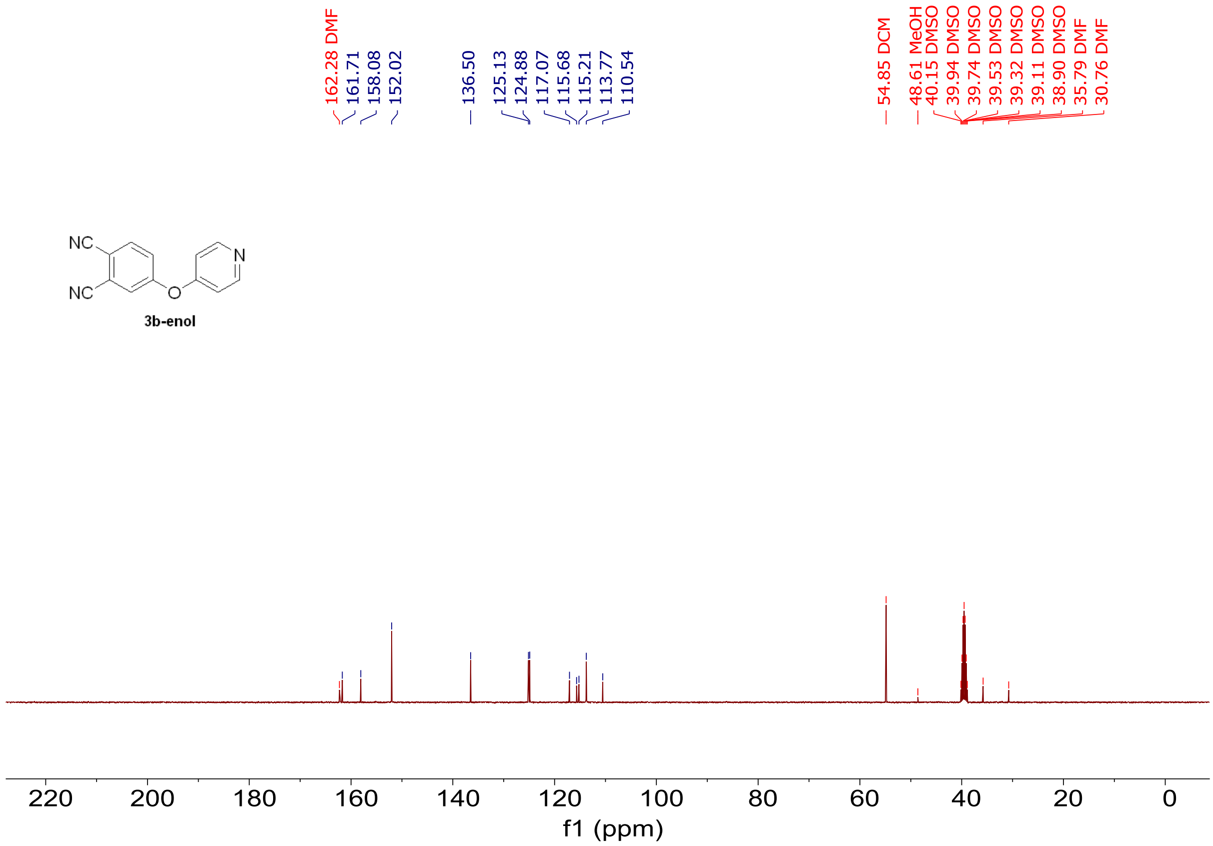

Figure S24: ^{13}C spectrum of **3b-*enol***.

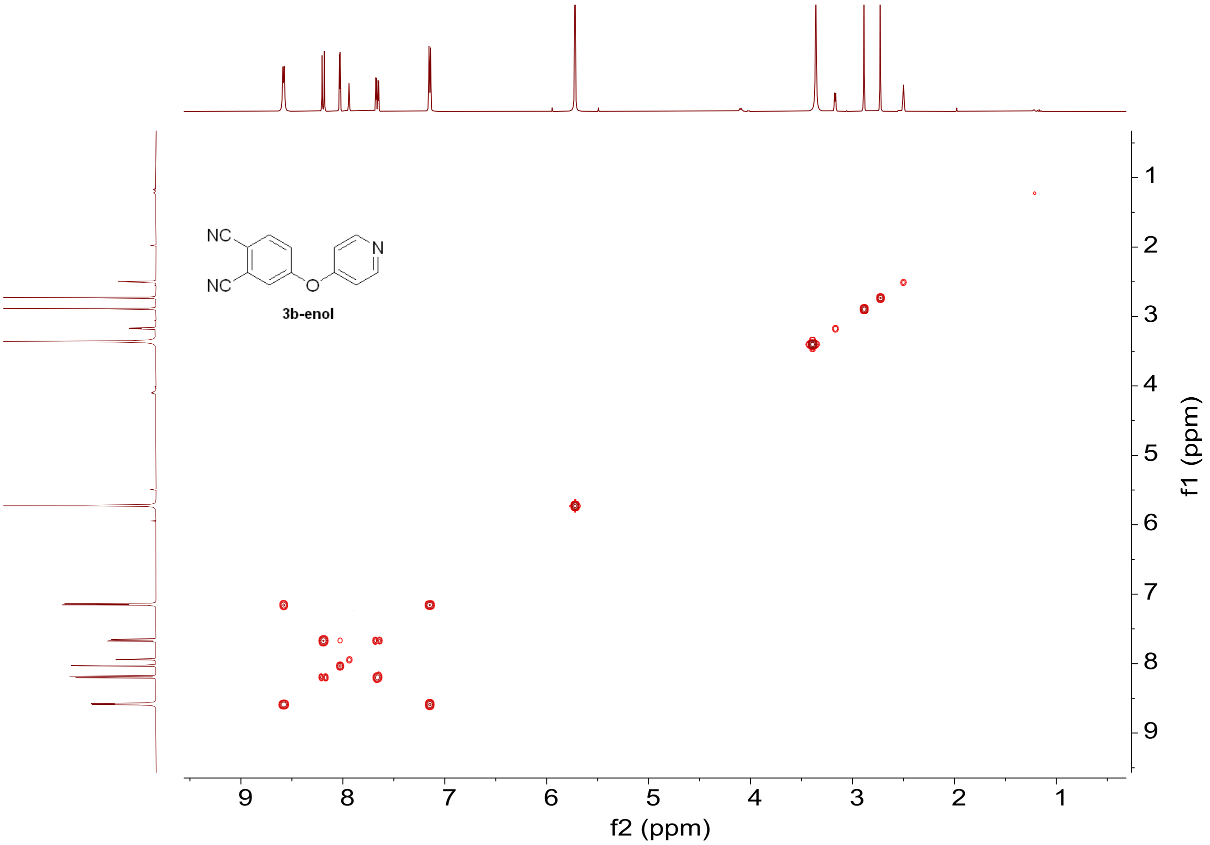

Figure S25: COSY spectrum of **3b-*enol***.

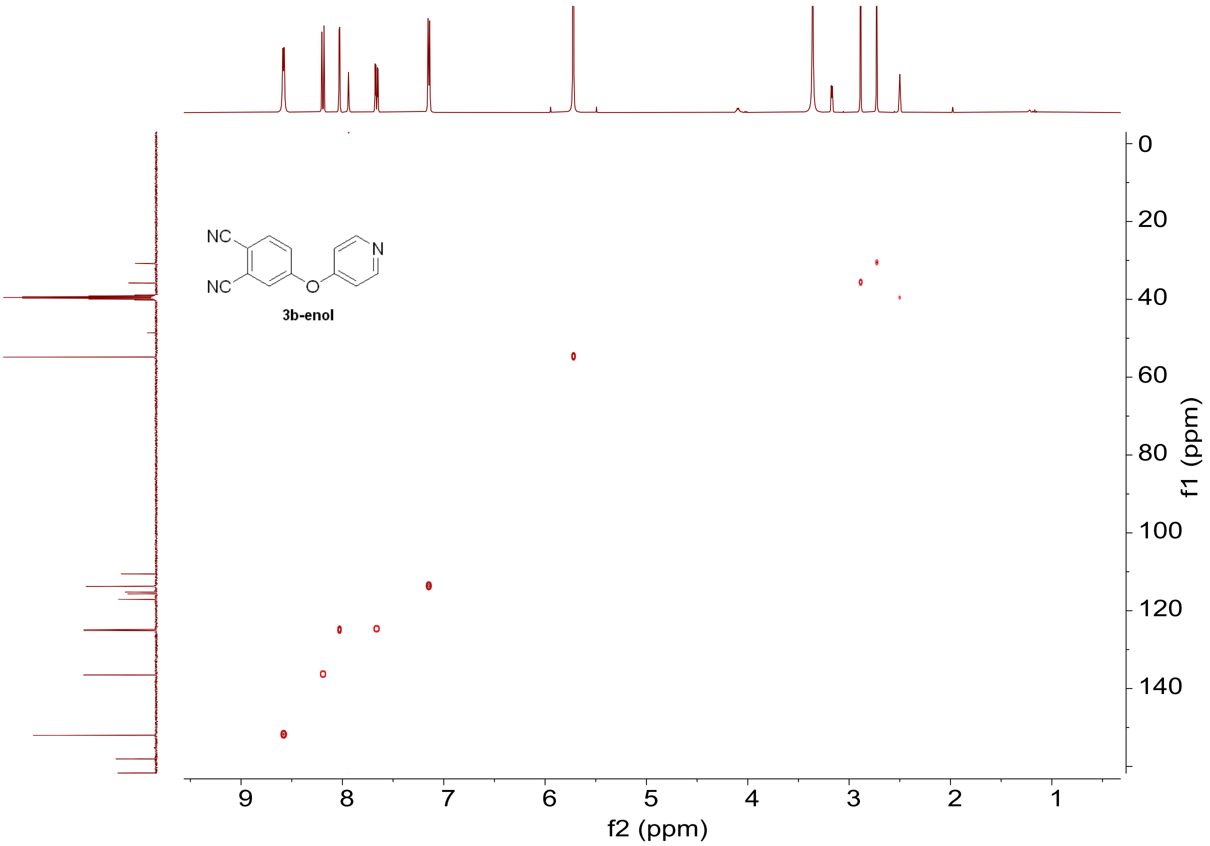

Figure S26: HSQC spectrum of **3b-*enol***.

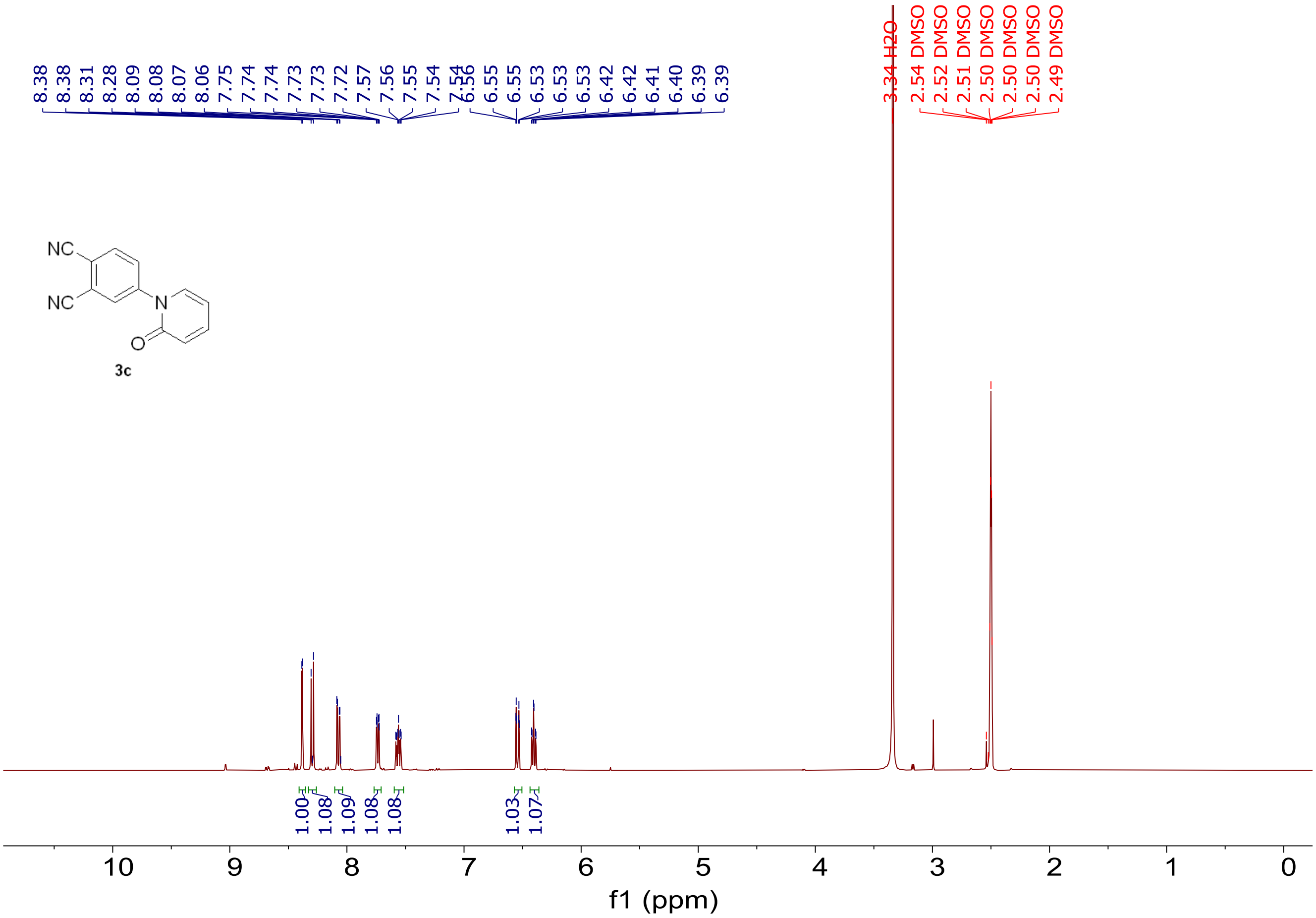

Figure S27: ^{1}H spectrum of **3c**.

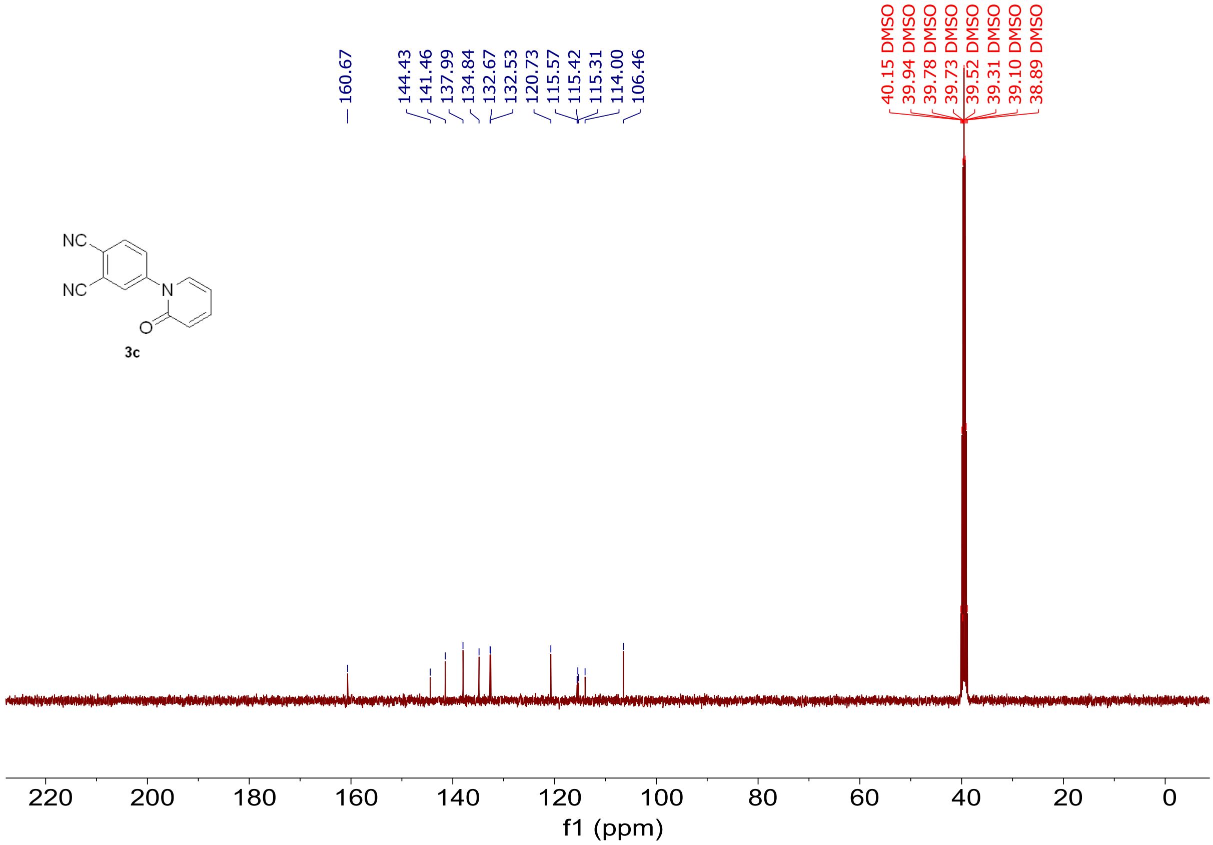

Figure S28: ^{13}C spectrum of **3c**.

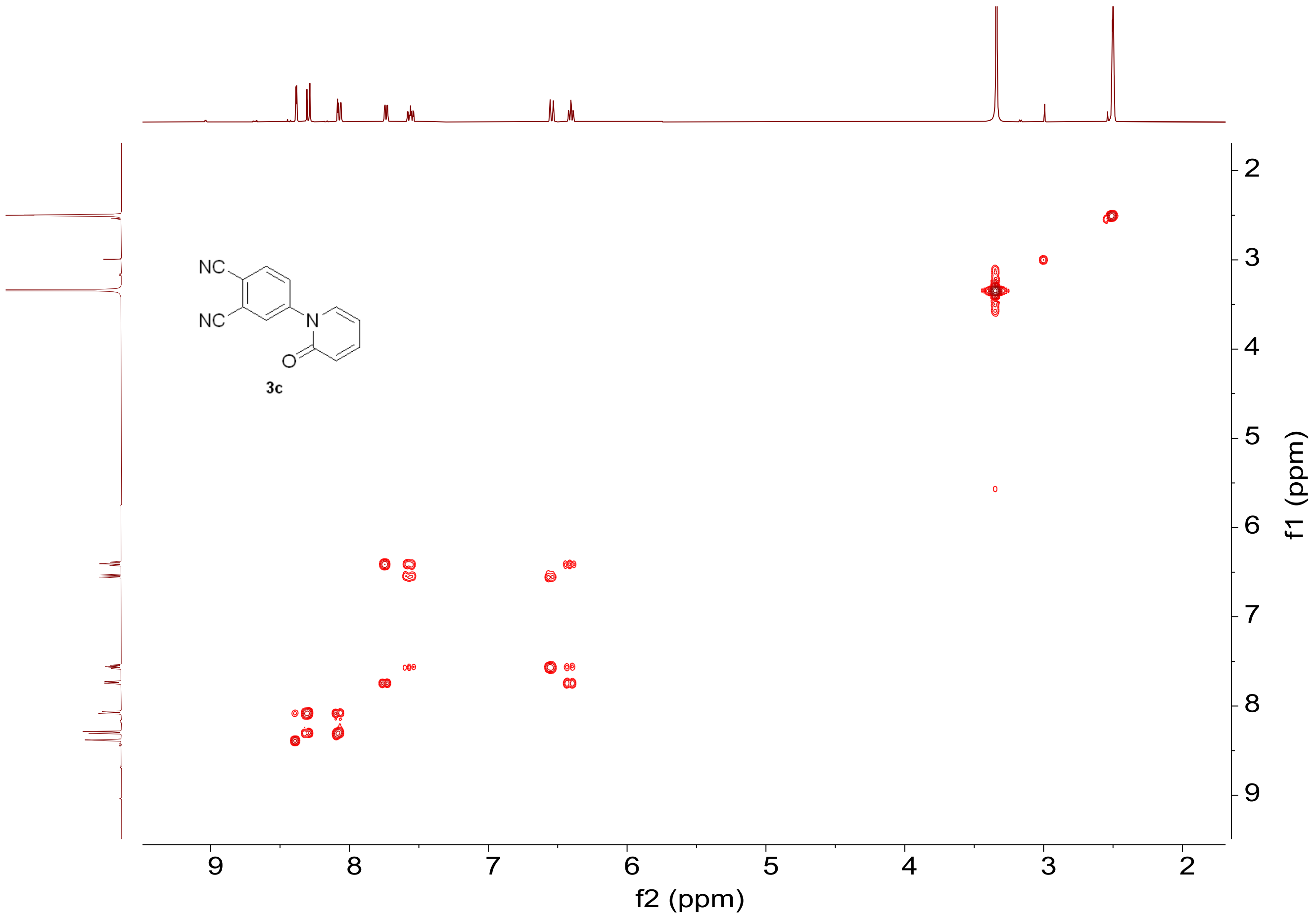

Figure S29: COSY spectrum of **3c**.

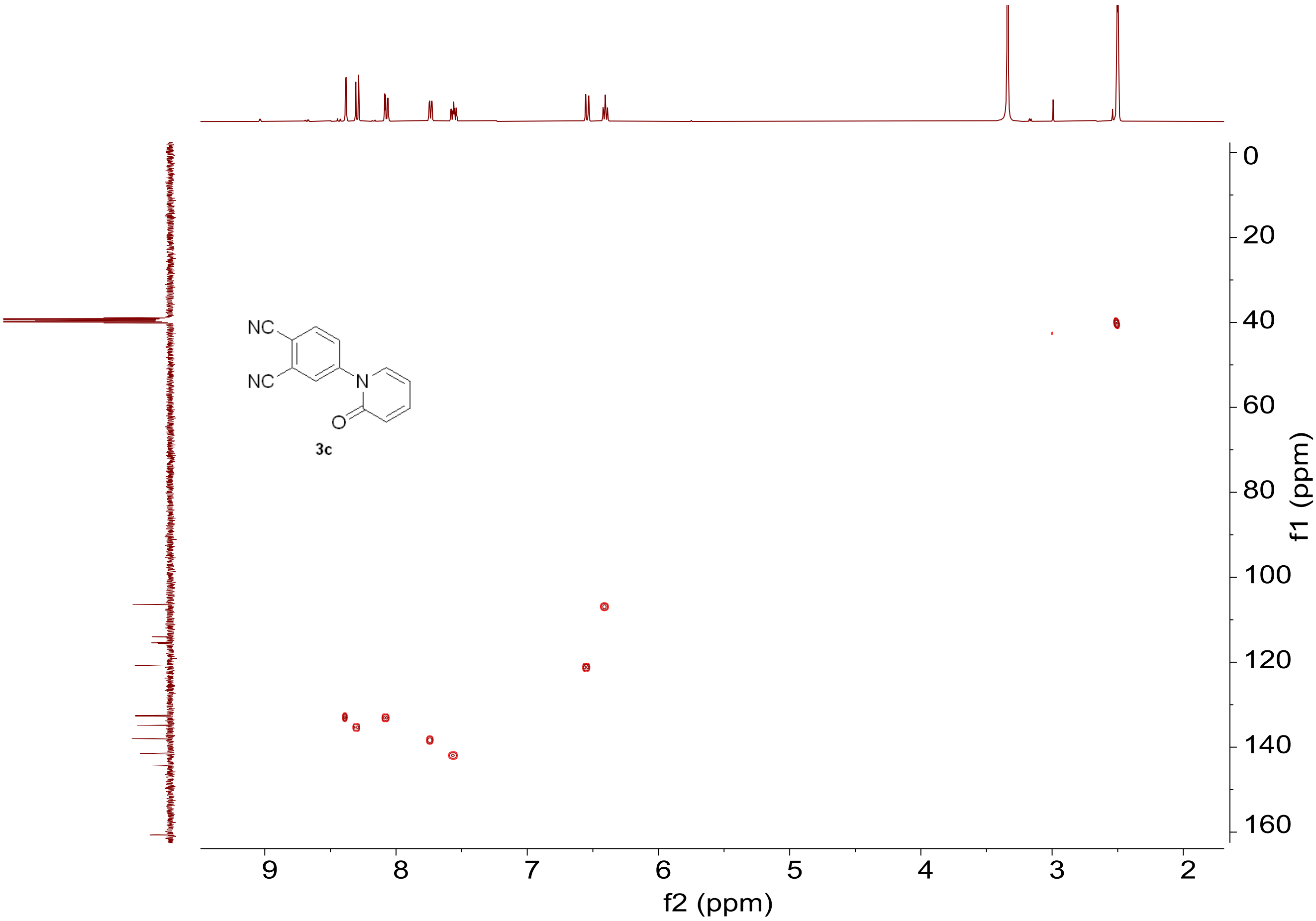

Figure S30: HSQC spectrum of **3c**.

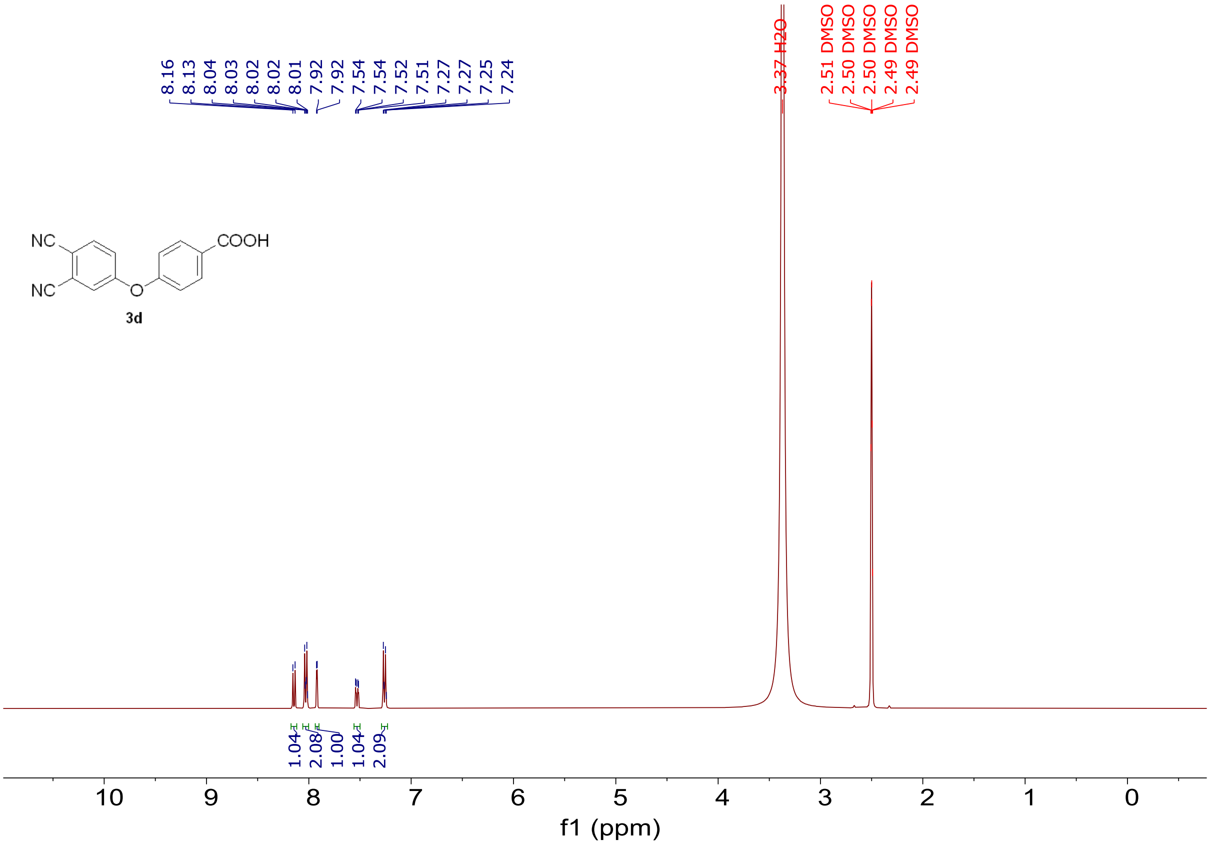

Figure S31: ^{1}H spectrum of **3d**.

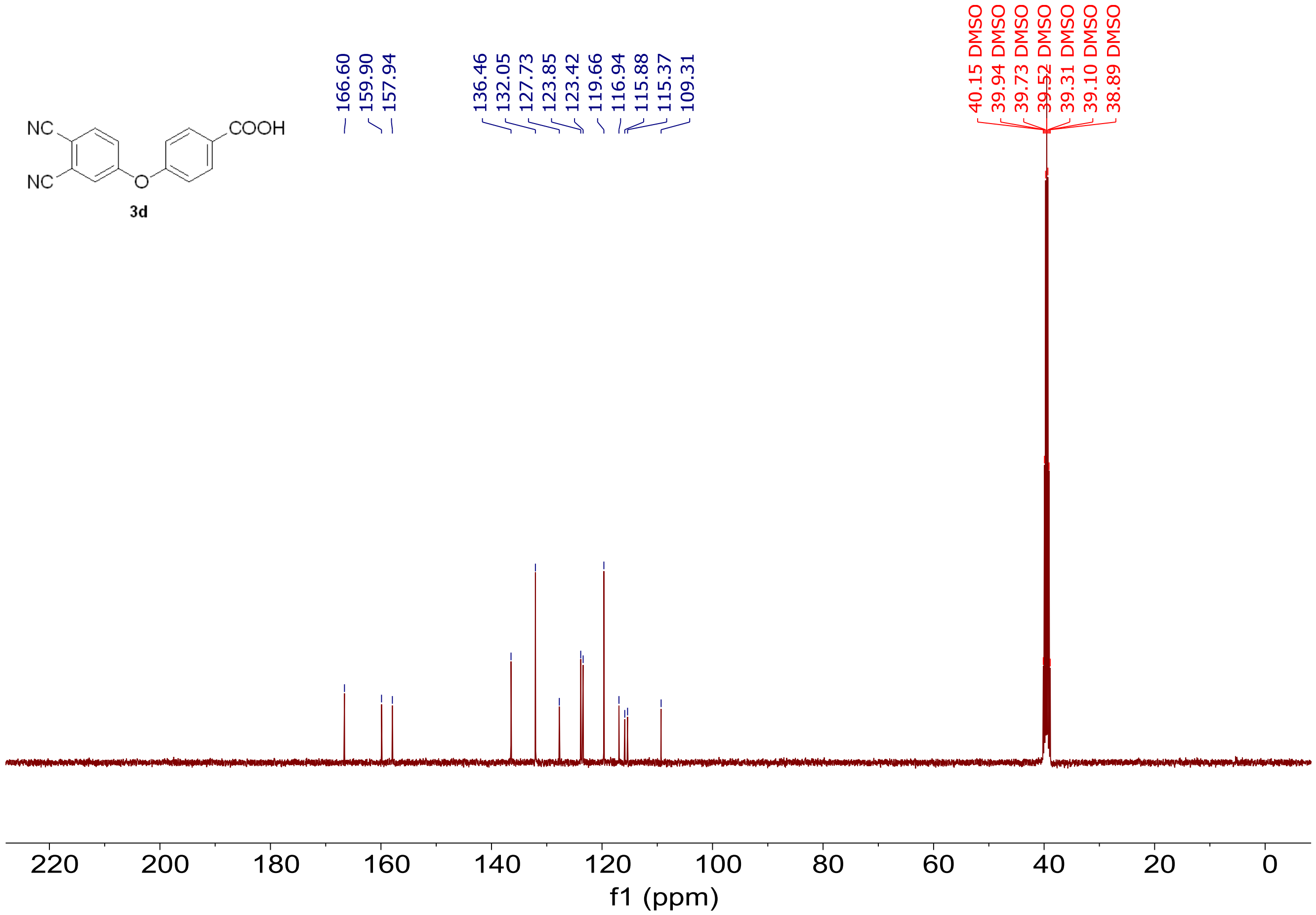

Figure S32: ^{13}C spectrum of **3d**.

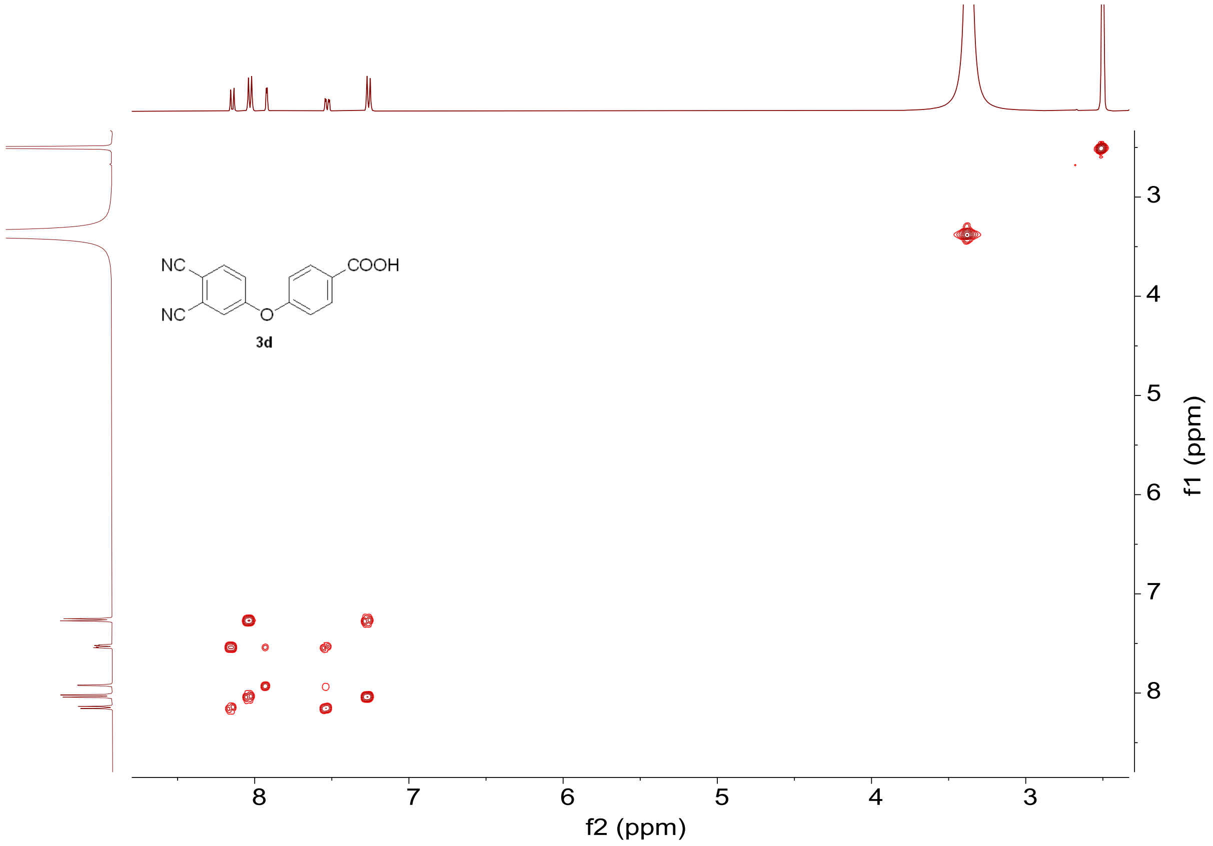

Figure S33: COSY spectrum of **3d**.

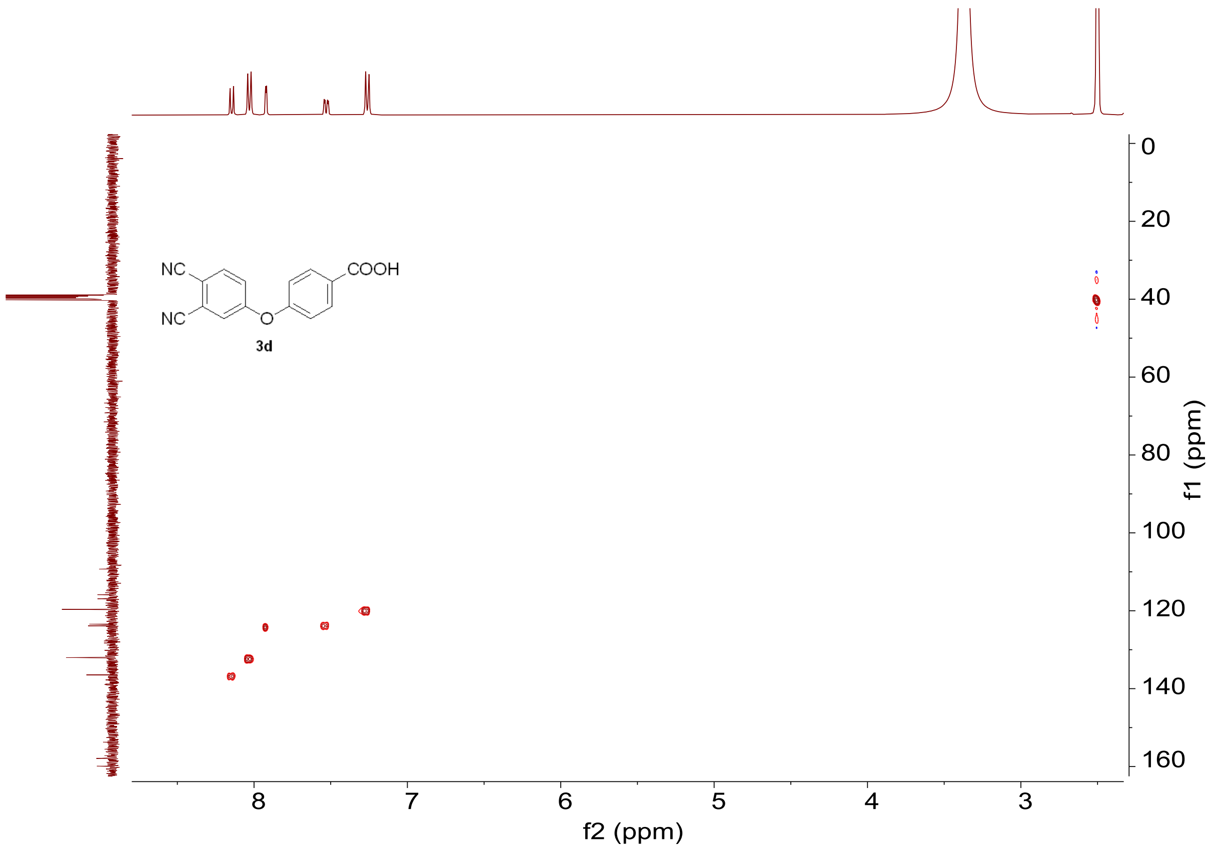

Figure S34: HSQC spectrum of **3d**.

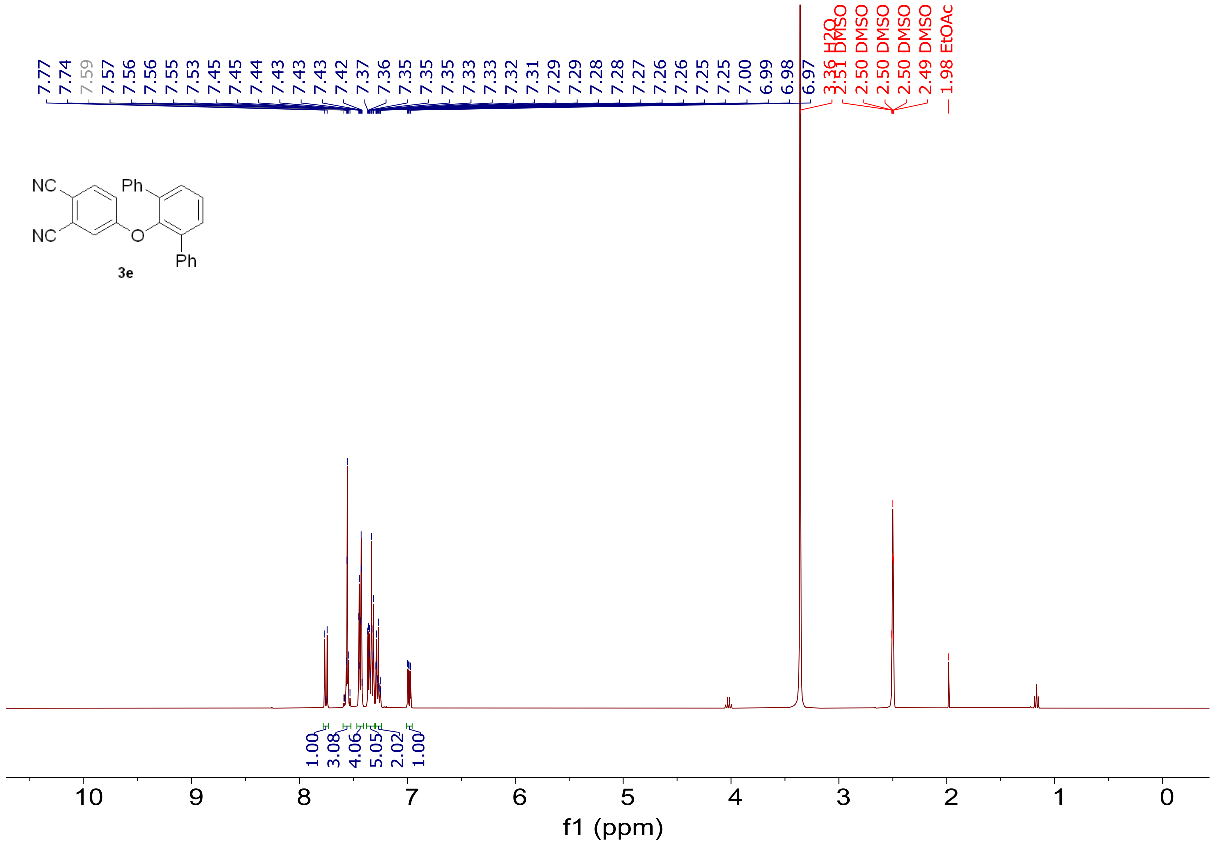

Figure S35: ^{1}H spectrum of **3e**.

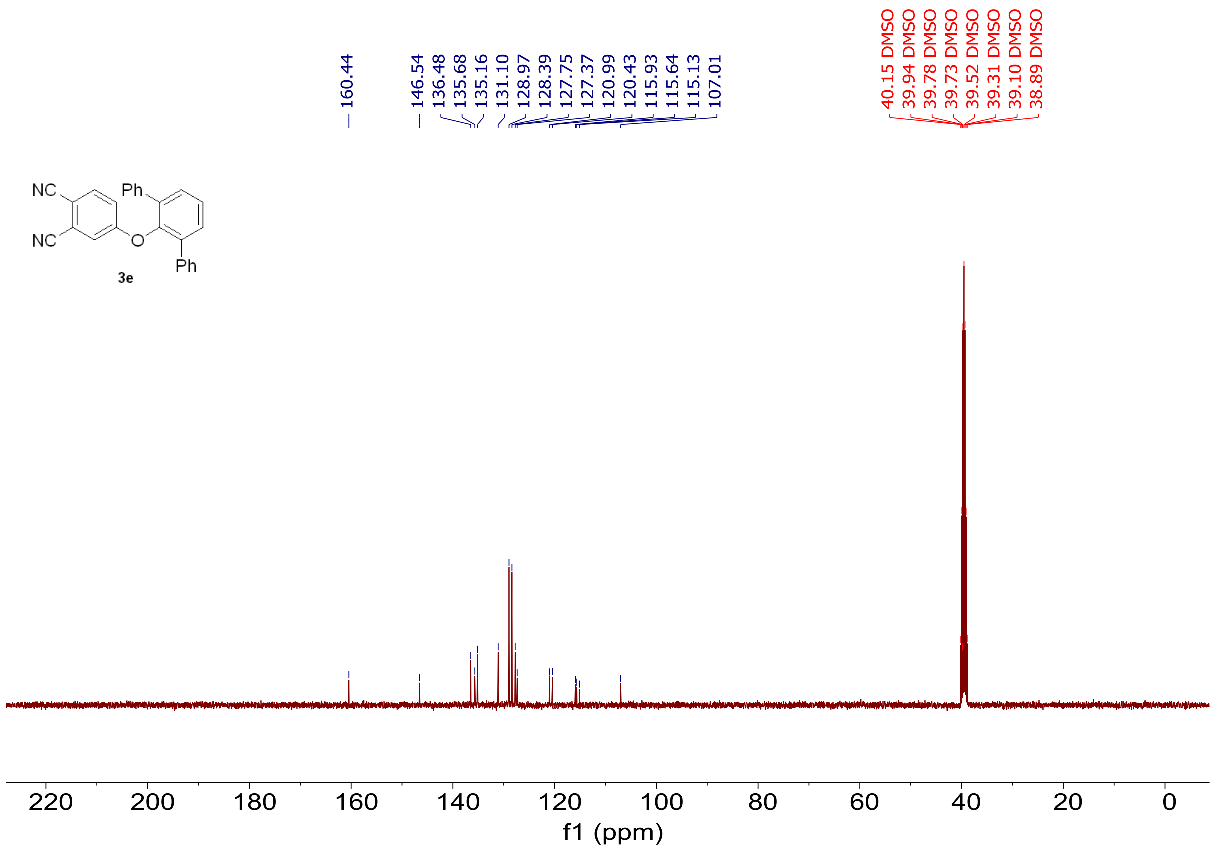

Figure S36: ^{13}C spectrum of **3e**.

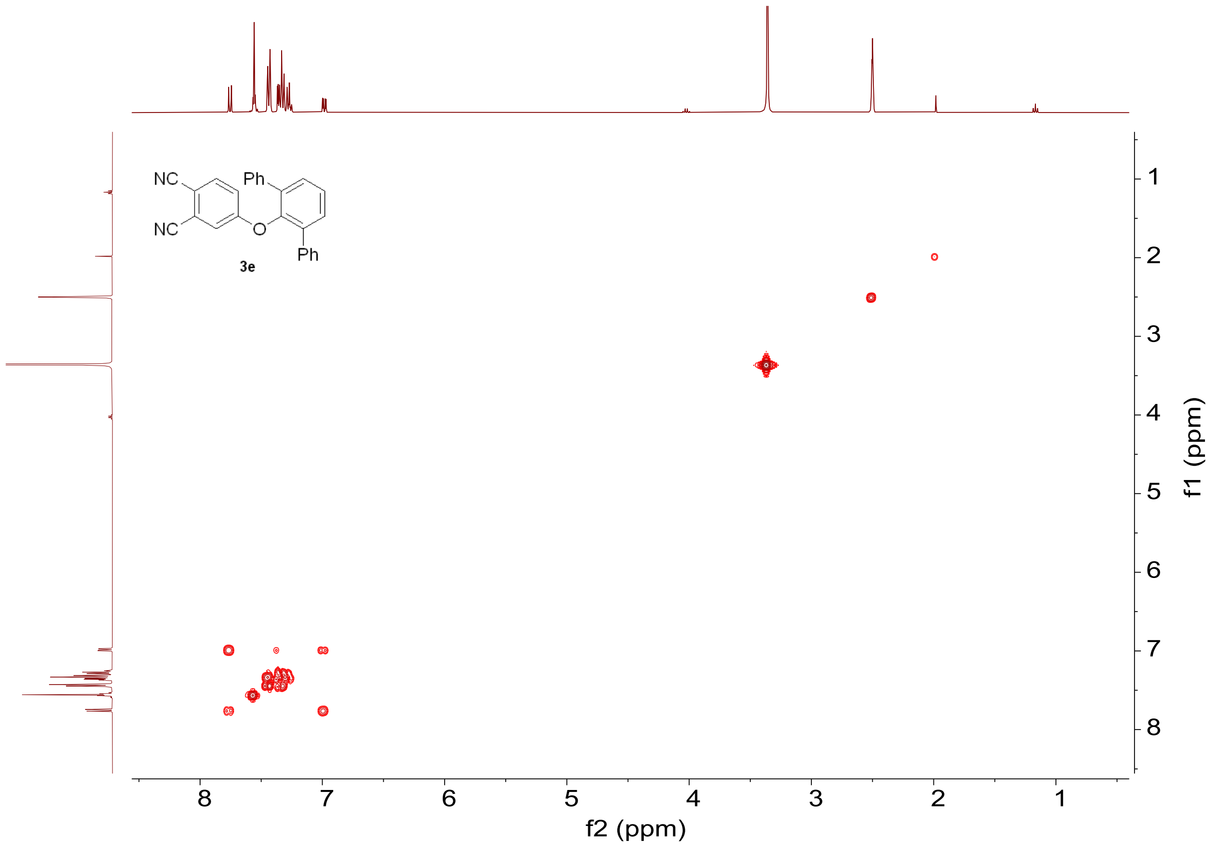

Figure S37: COSY spectrum of **3e**.

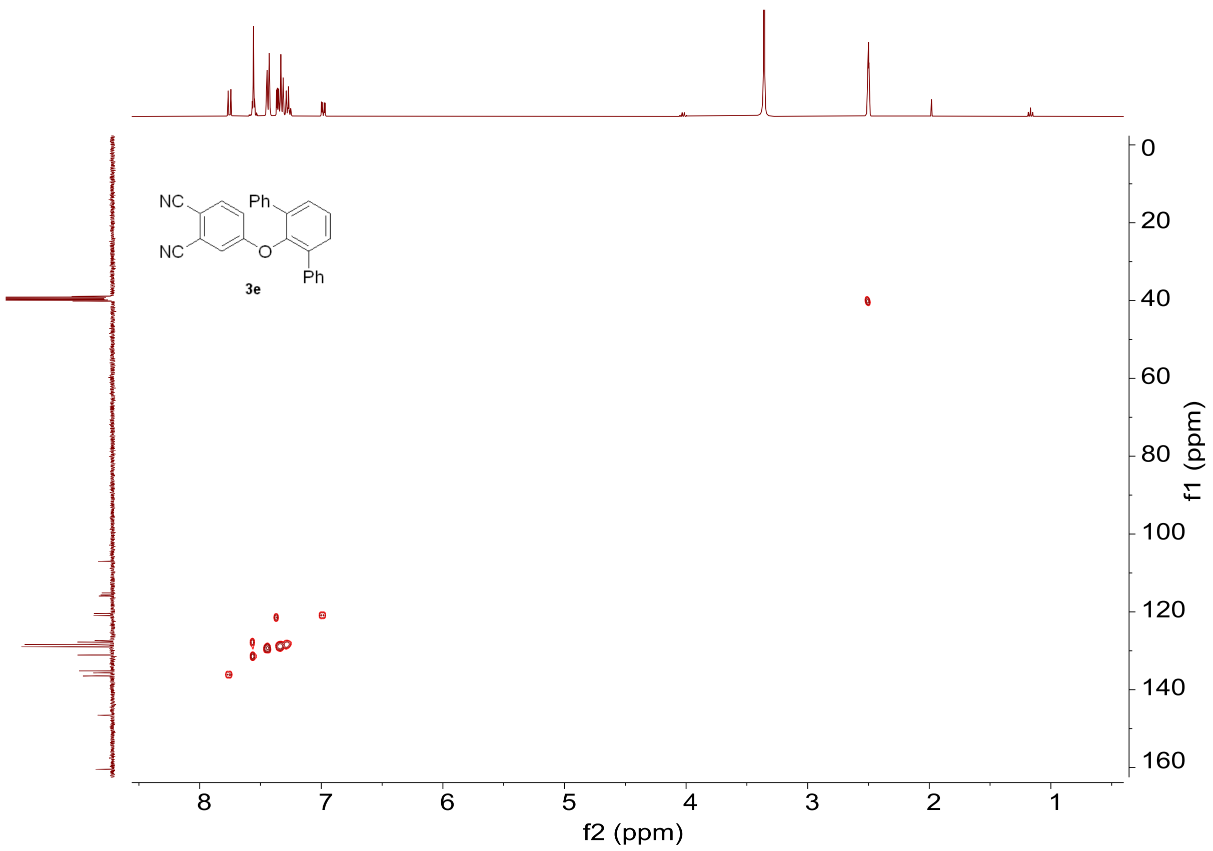

Figure S38: HSQC spectrum of **3e**.

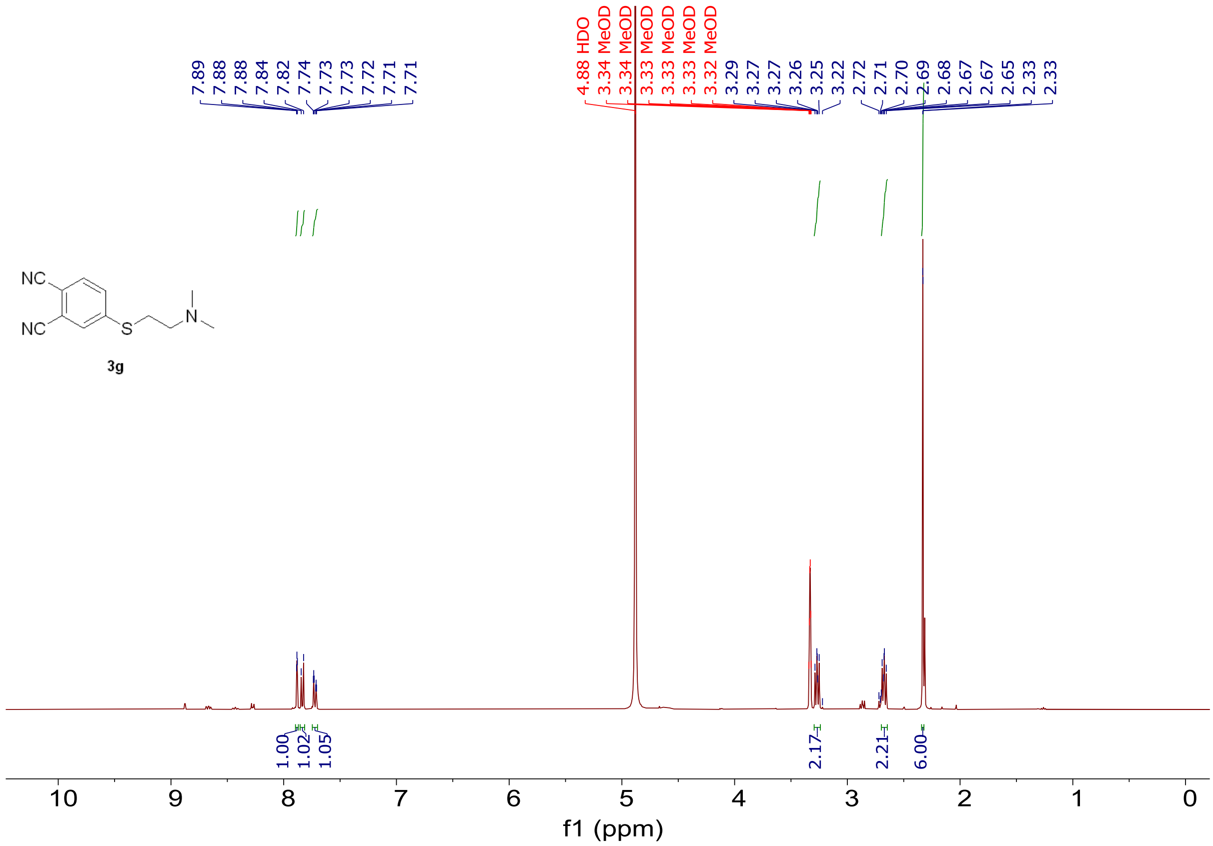

Figure S39: ^{1}H spectrum of **3g**.

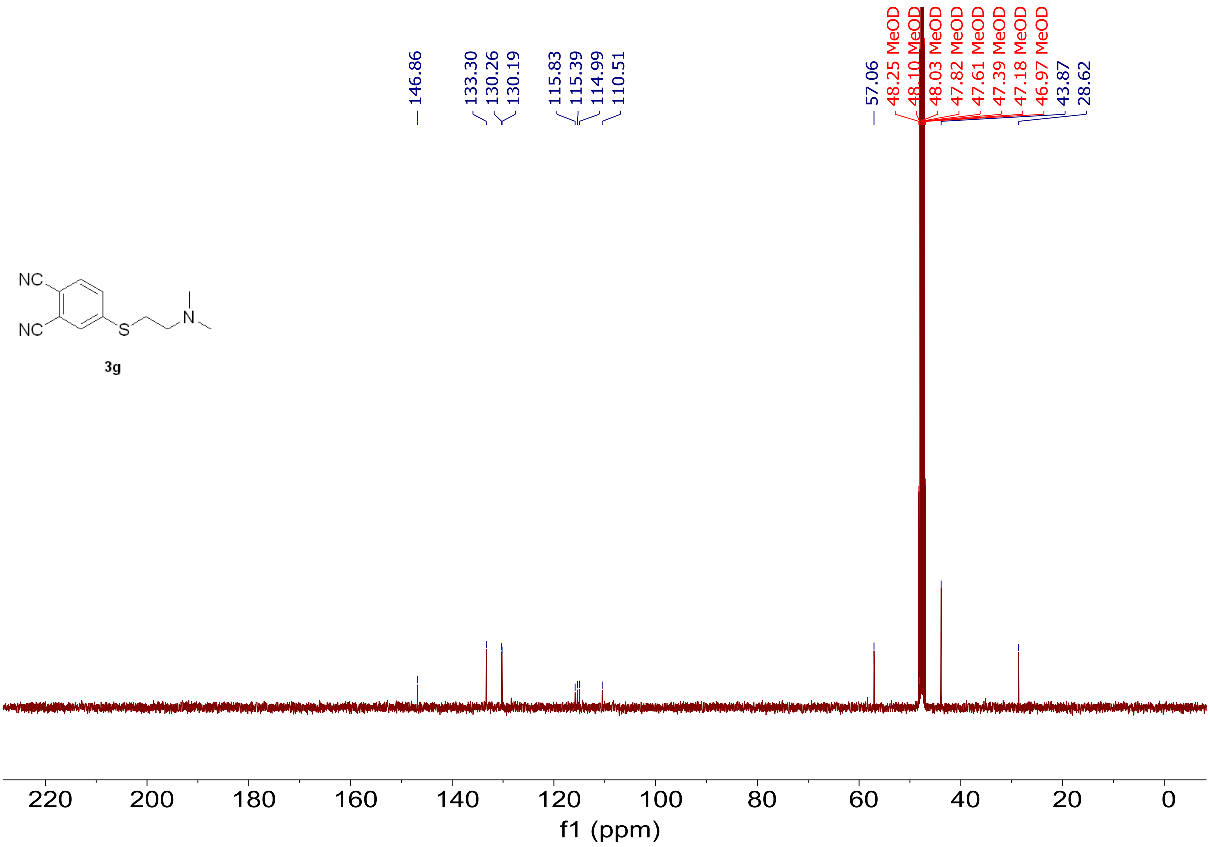

Figure S40: ^{13}C spectrum of **3g**.

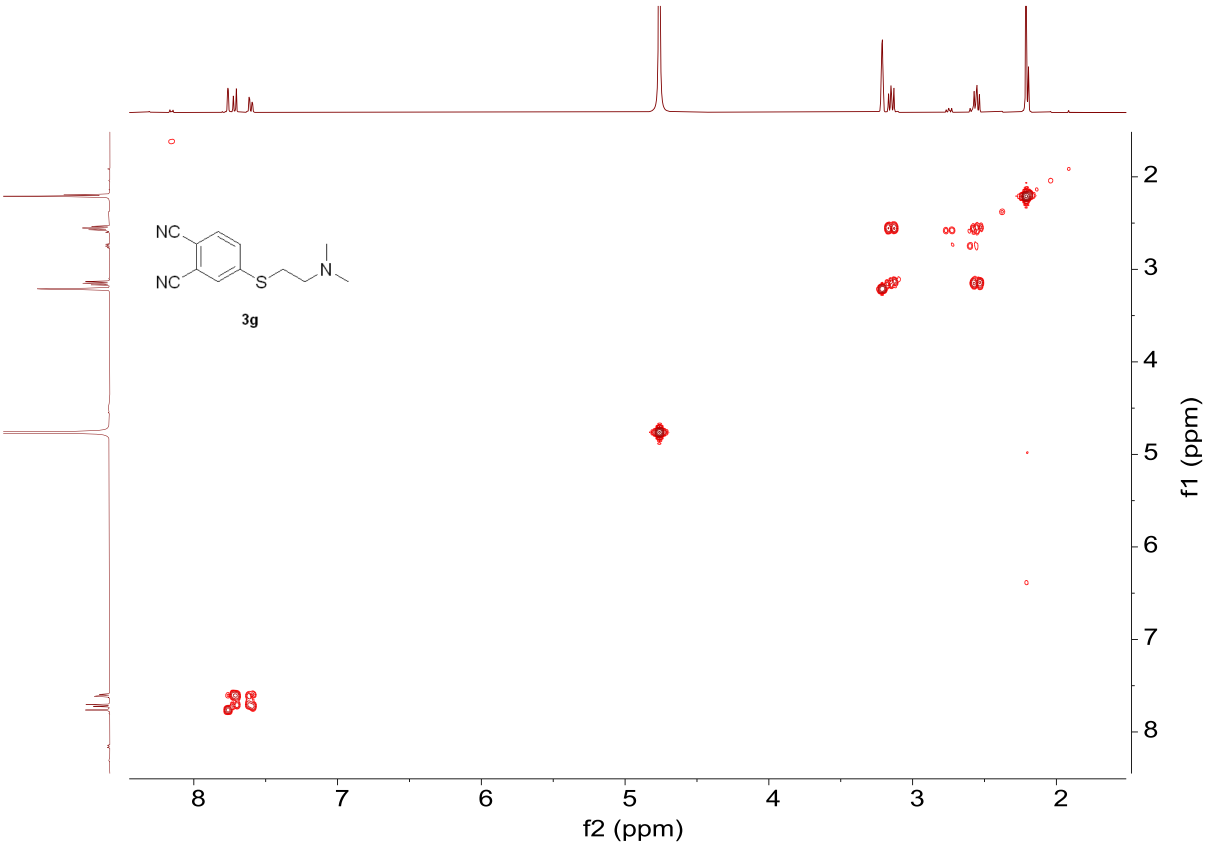

Figure S41: COSY spectrum of **3g**.

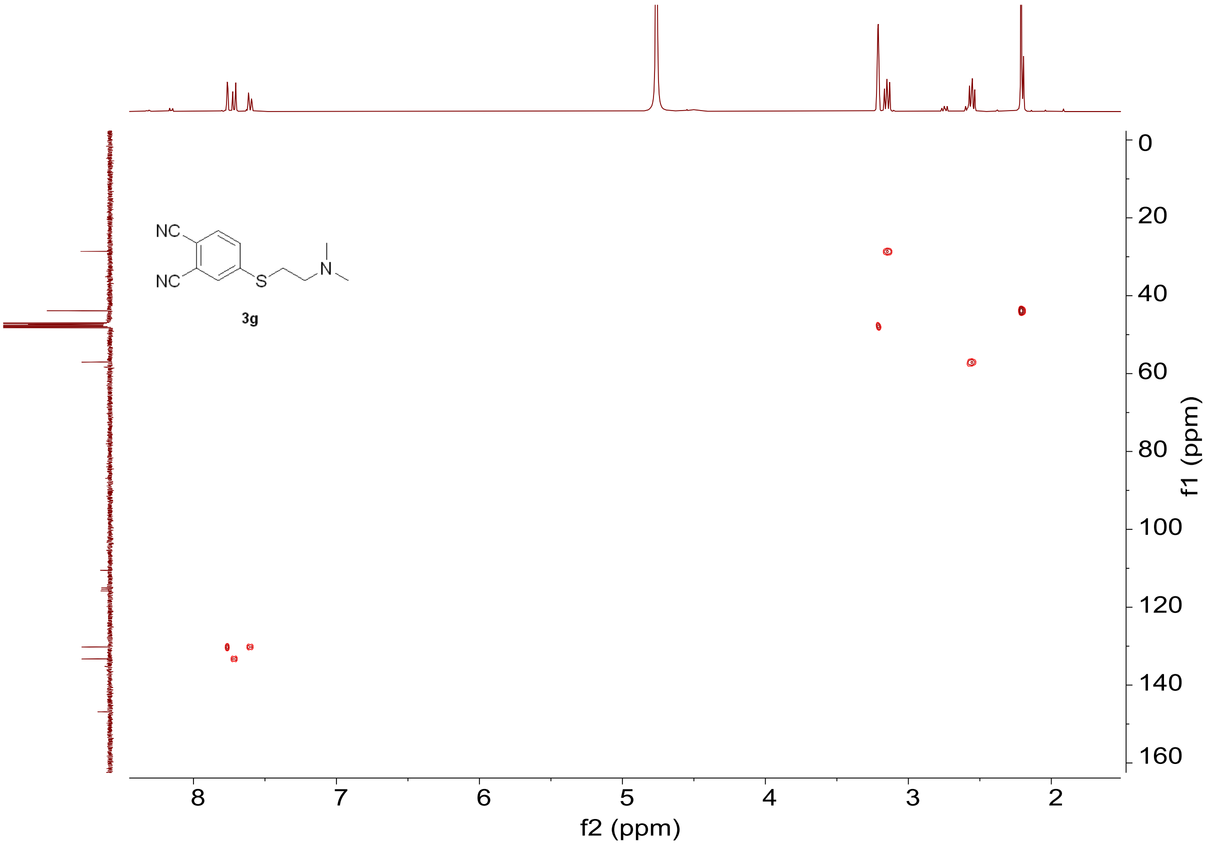

Figure S42: HSQC spectrum of **3g**.

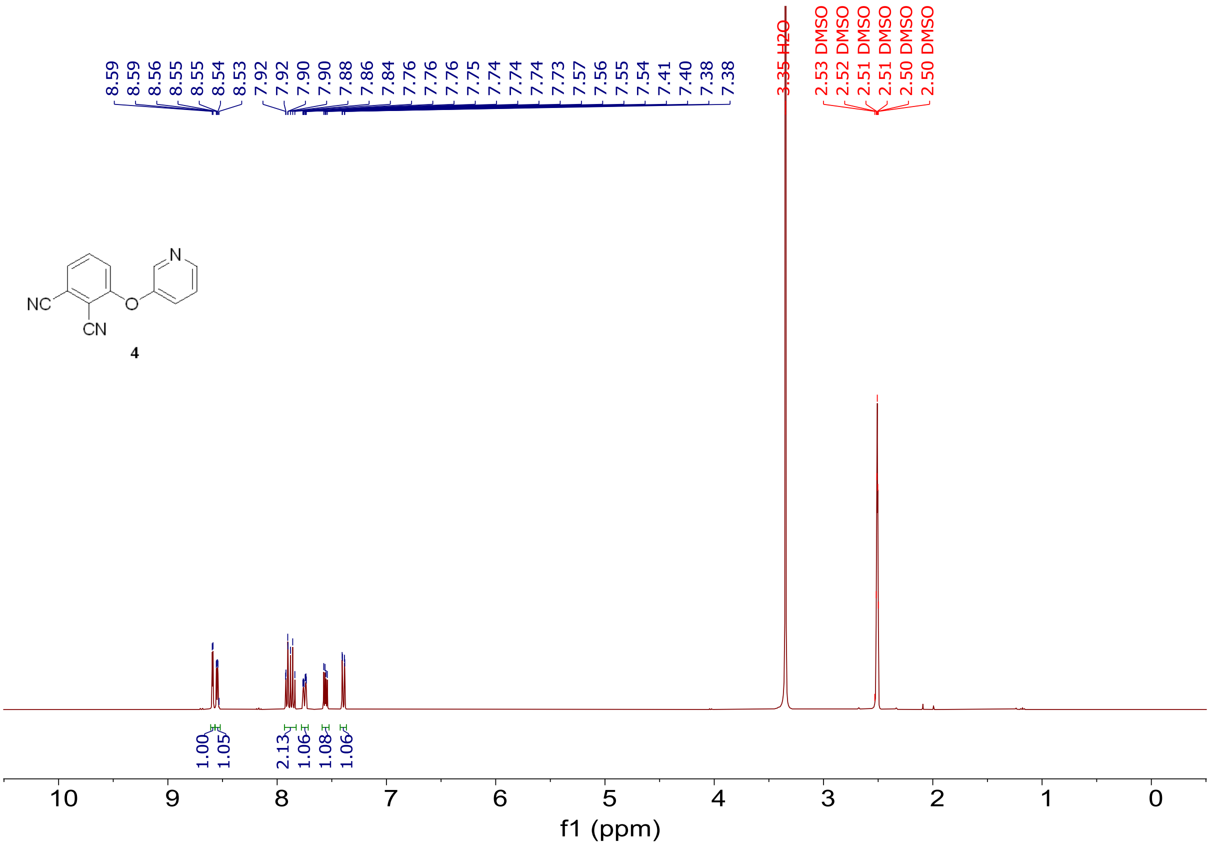

Figure S43: ^{1}H spectrum of **4**.

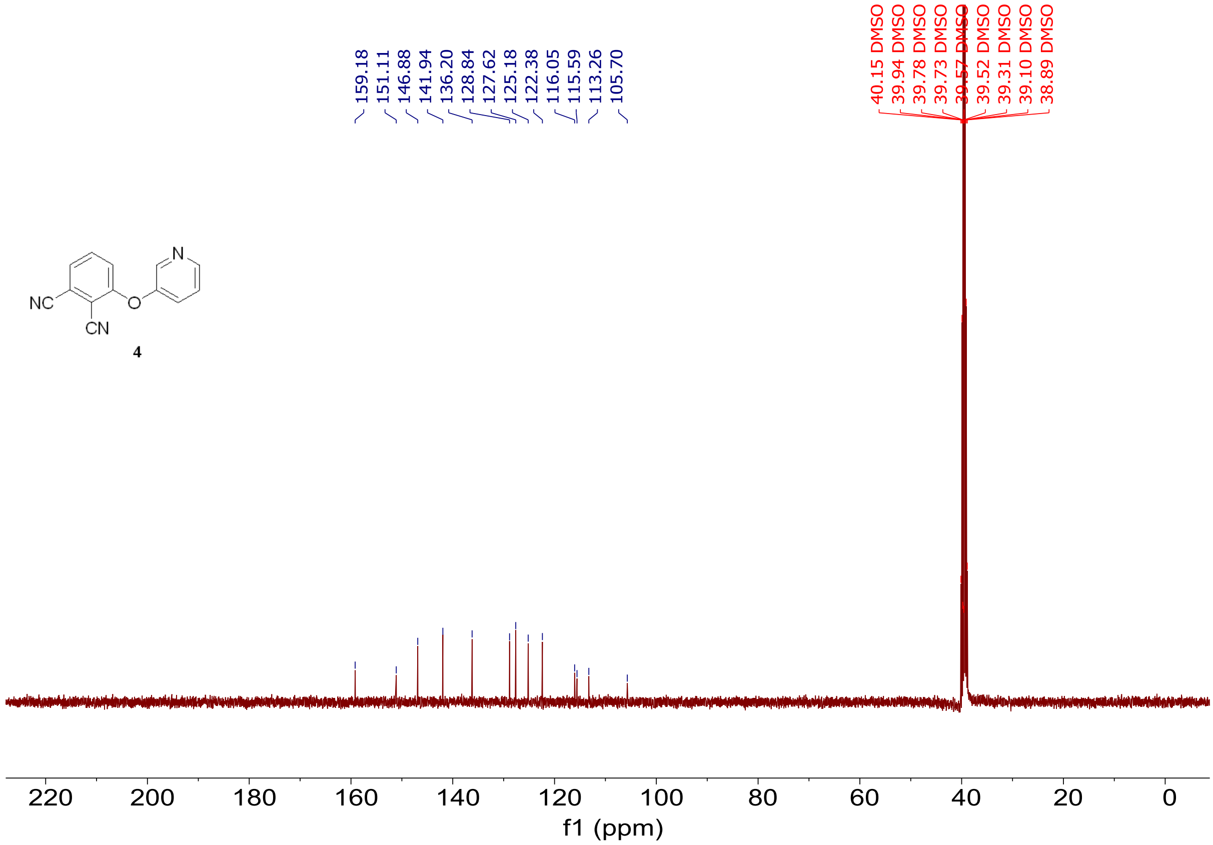

Figure S44: ^{13}C spectrum of **4**.

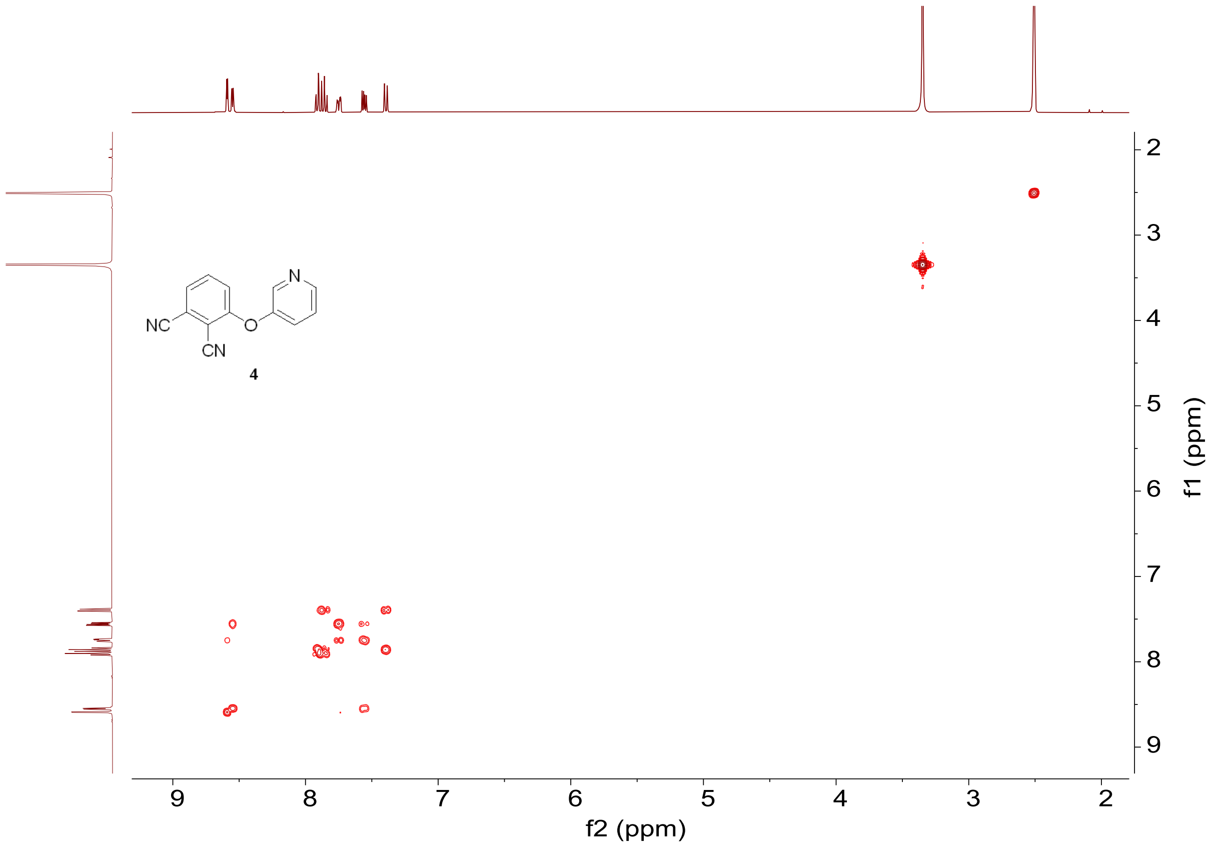

Figure S45: COSY spectrum of **4**.

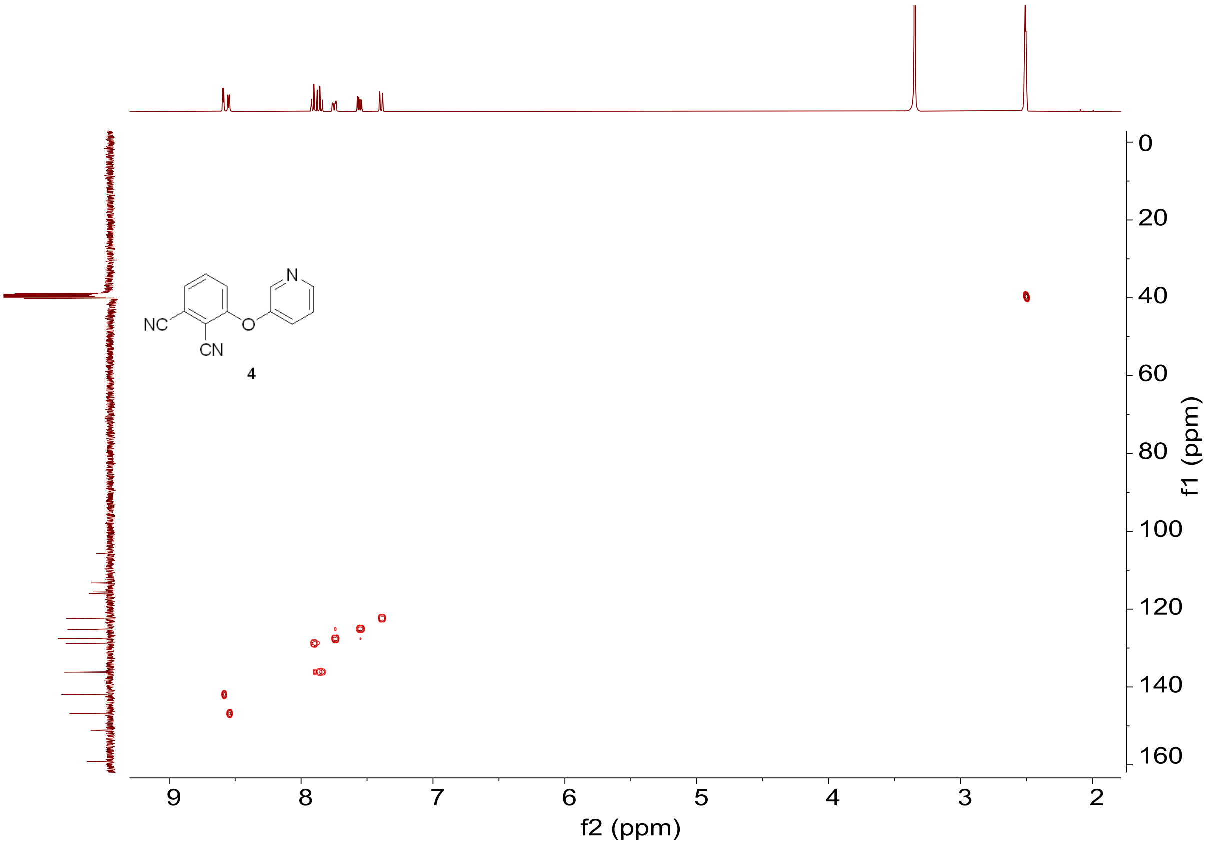

Figure S46: HSQC spectrum of **4**.

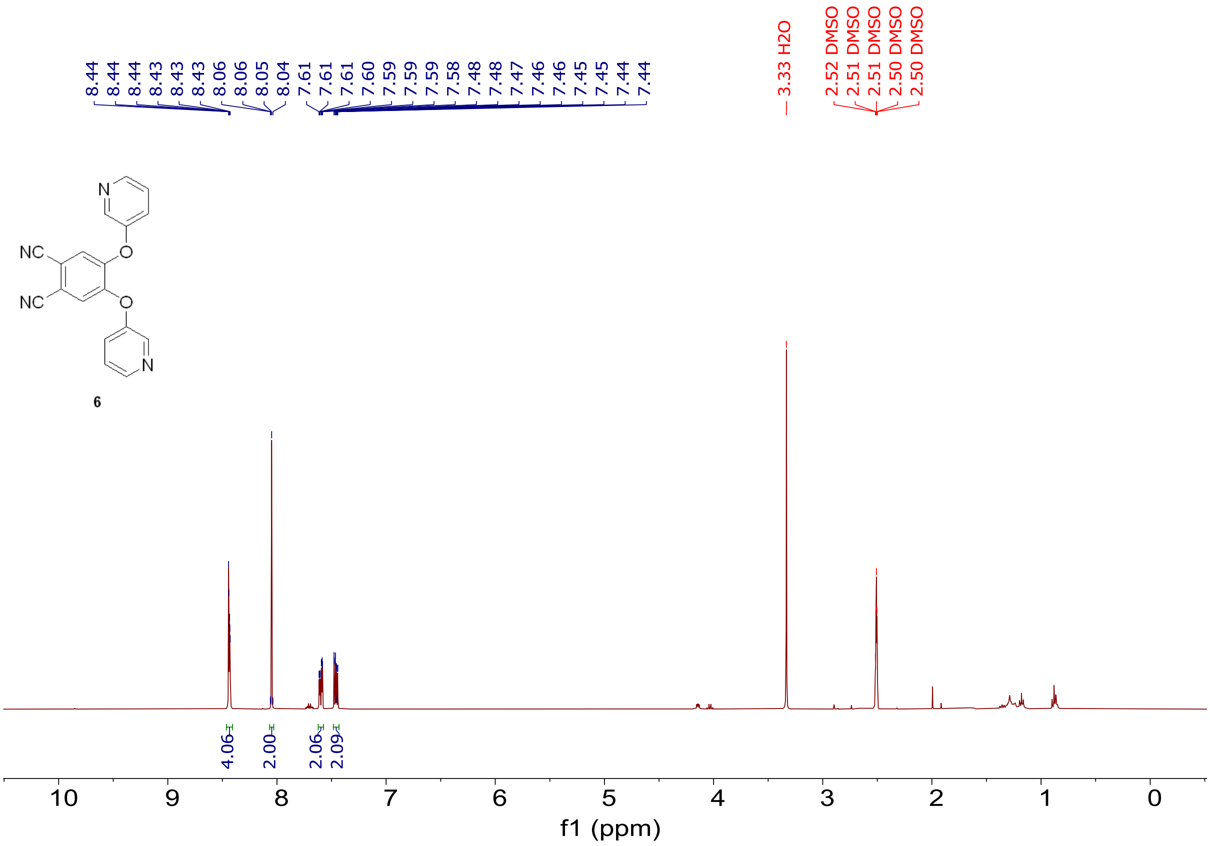

Figure S47: ^{1}H spectrum of **6**.

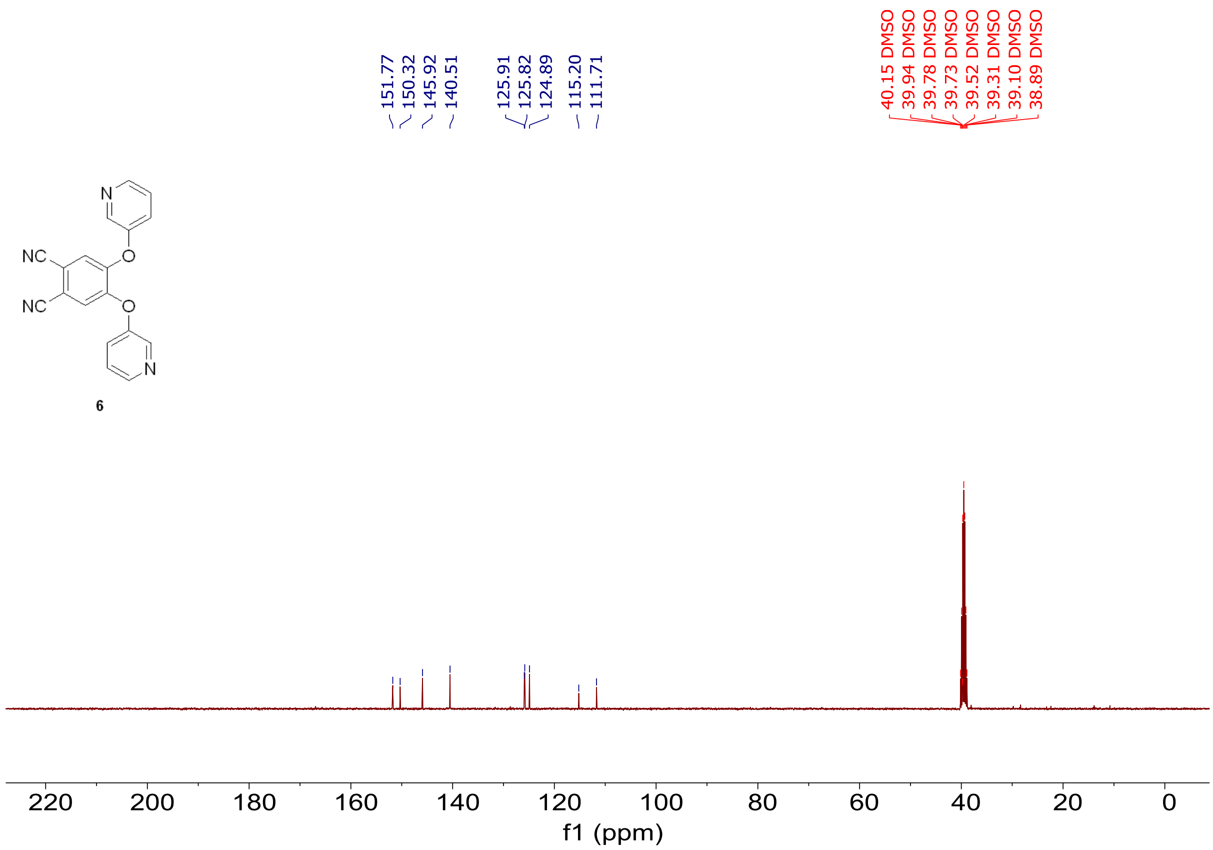

Figure S48: ^{13}C spectrum of **6**.

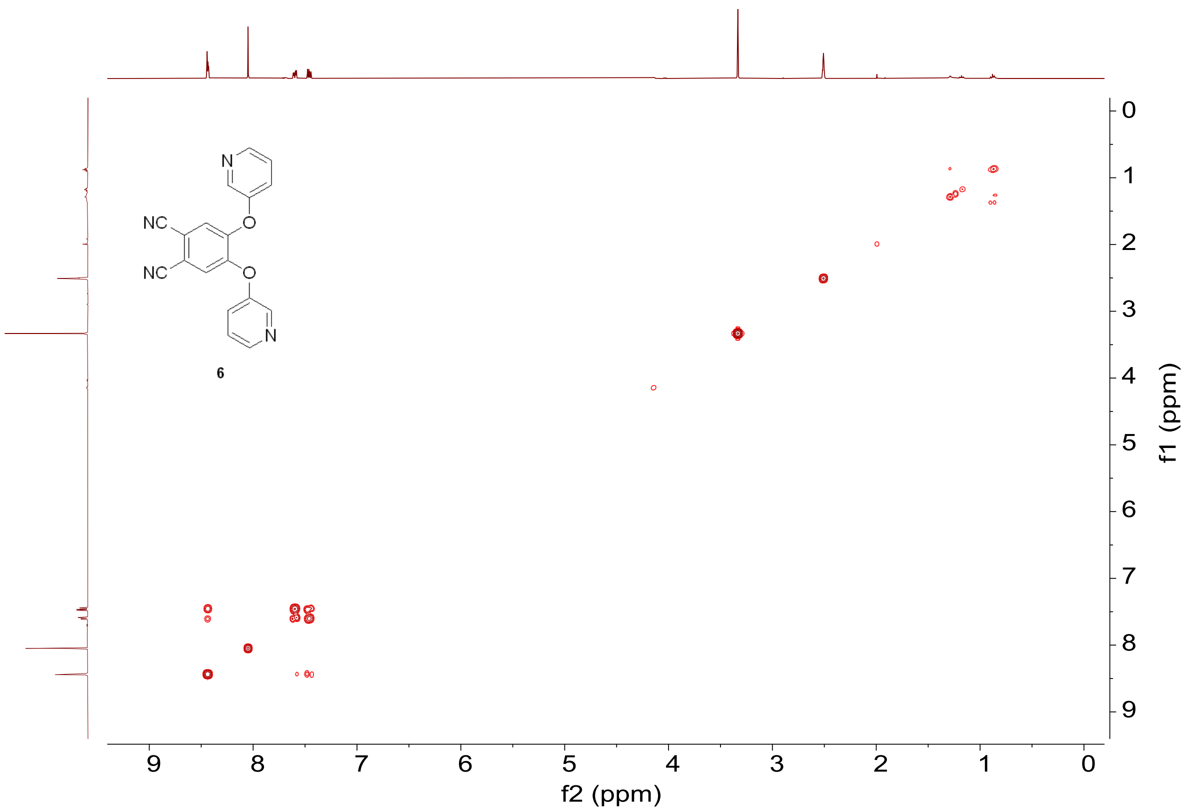

Figure S49: COSY spectrum of **6**.

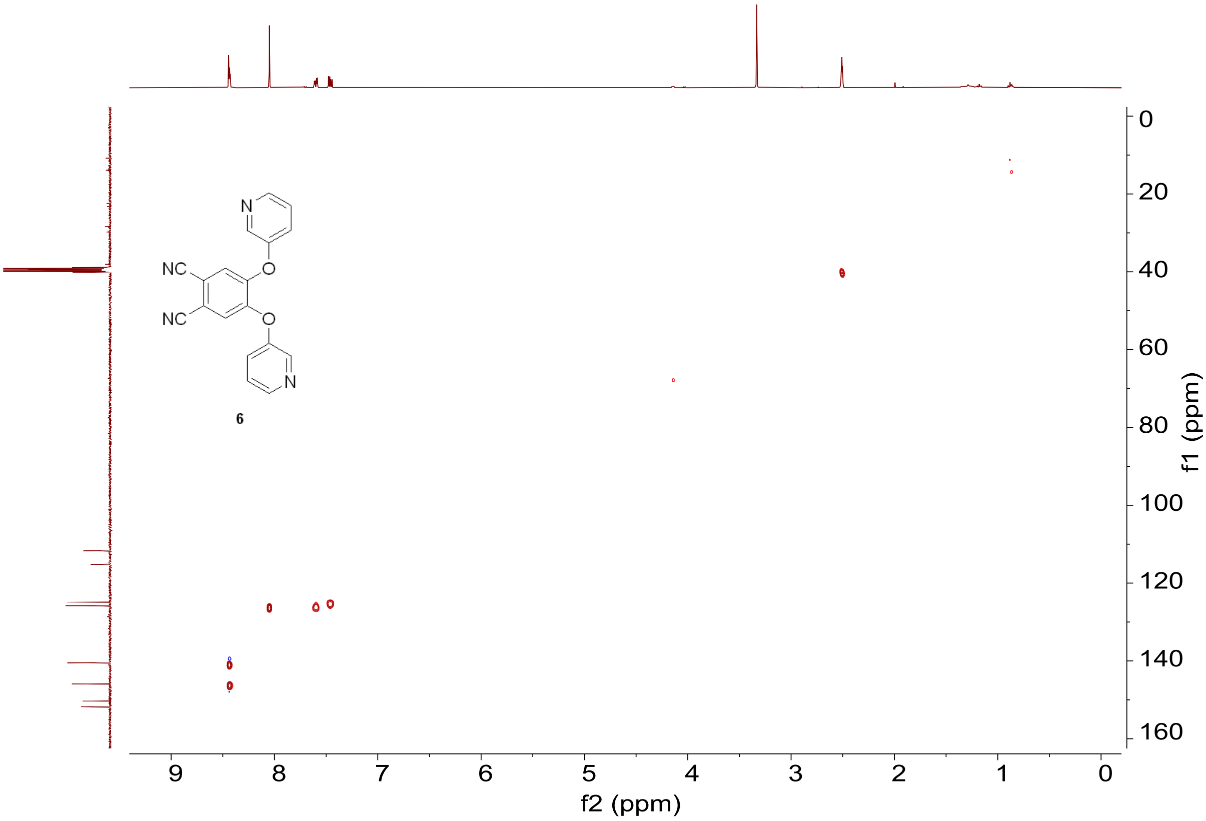

Figure S50: HSQC spectrum of **6**.

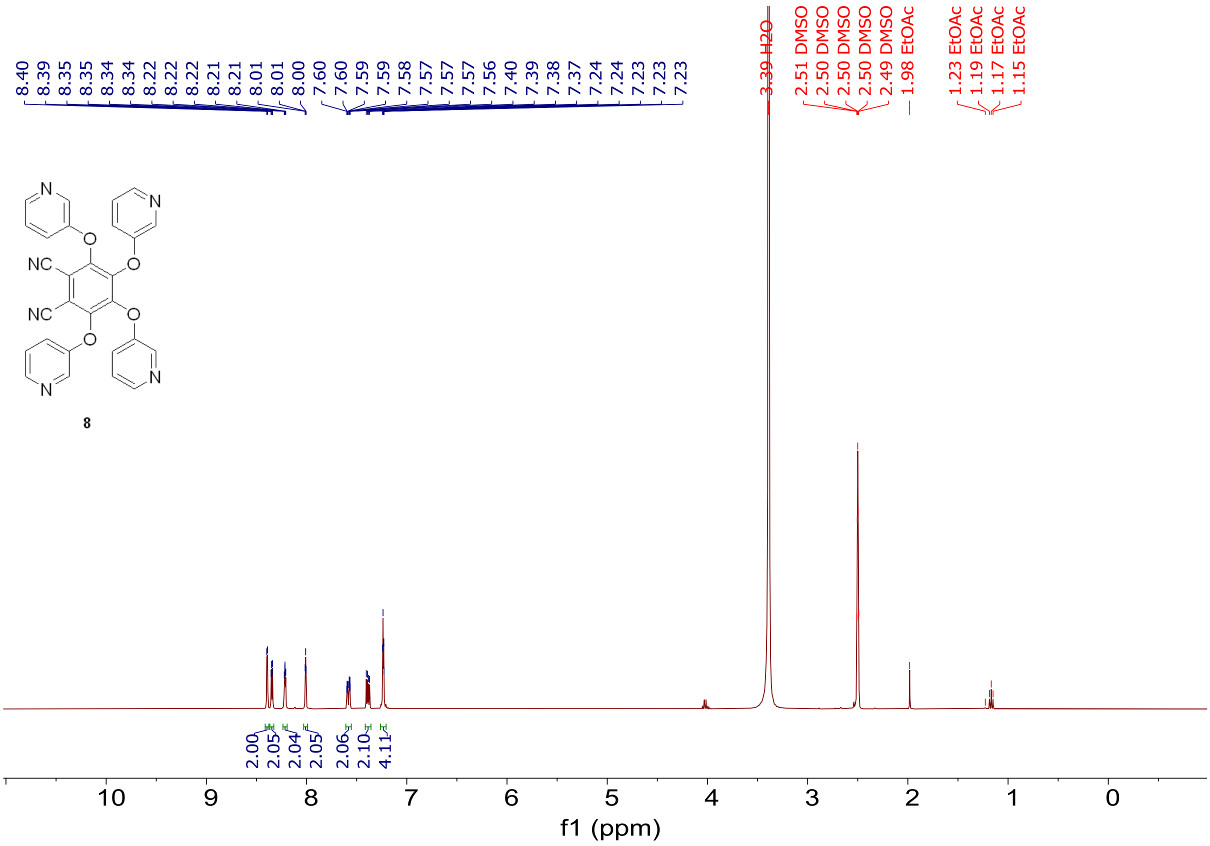

Figure S51: ^{1}H spectrum of **8**.

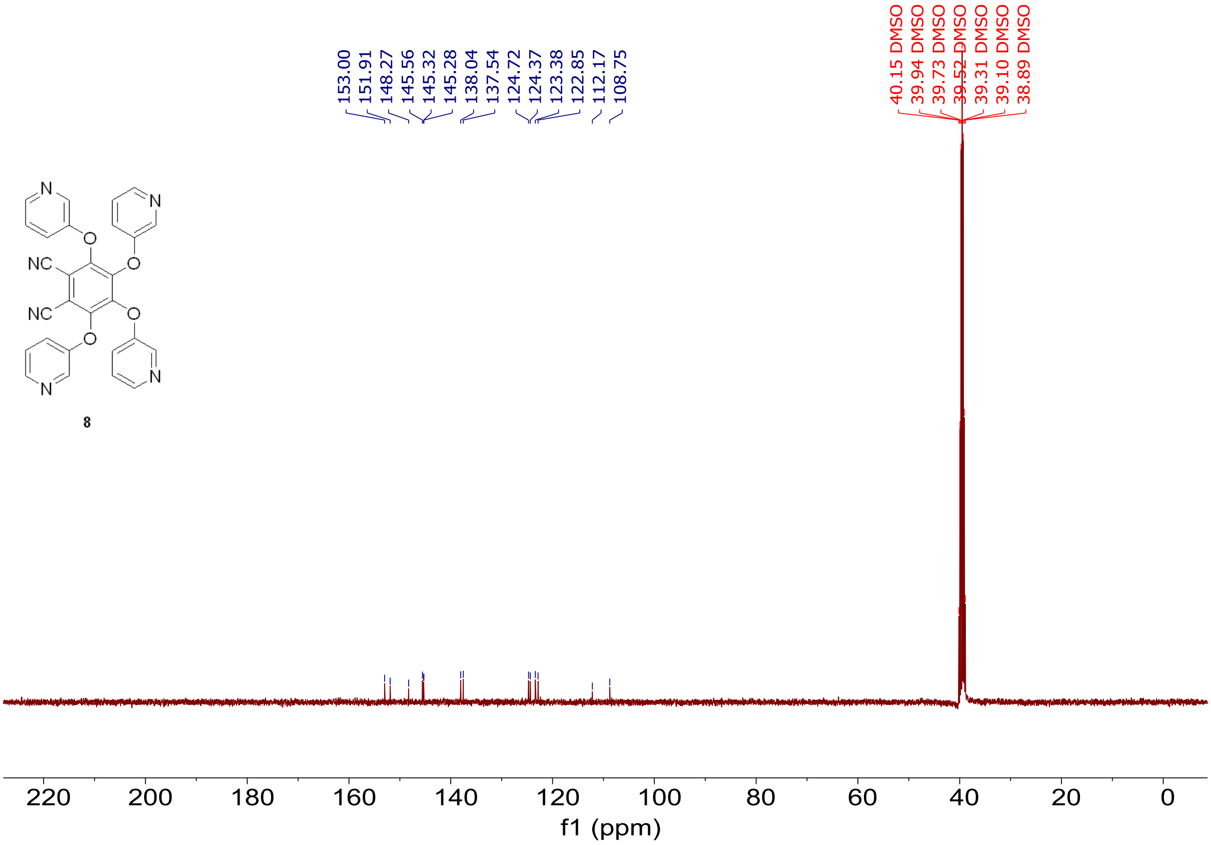

Figure S52: ^{13}C spectrum of **8**.

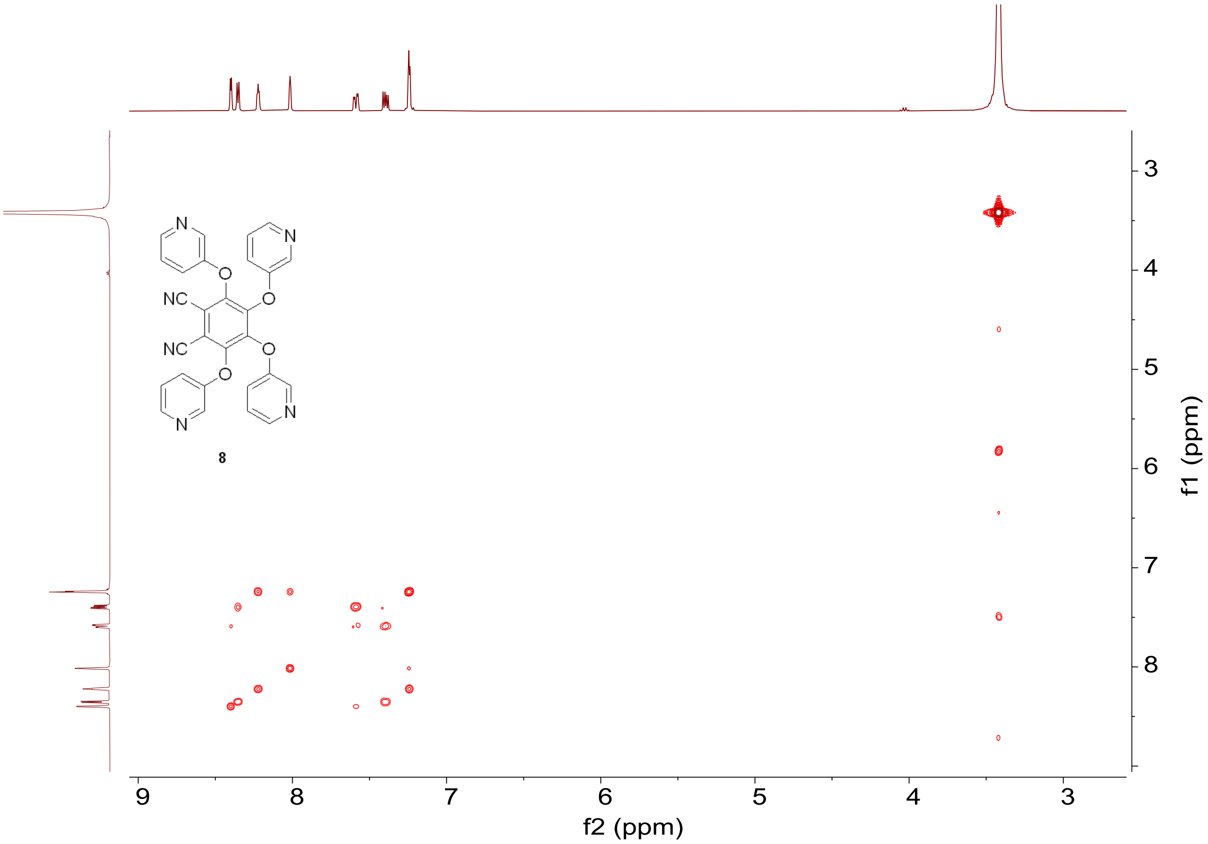

Figure S53: COSY spectrum of **8**.

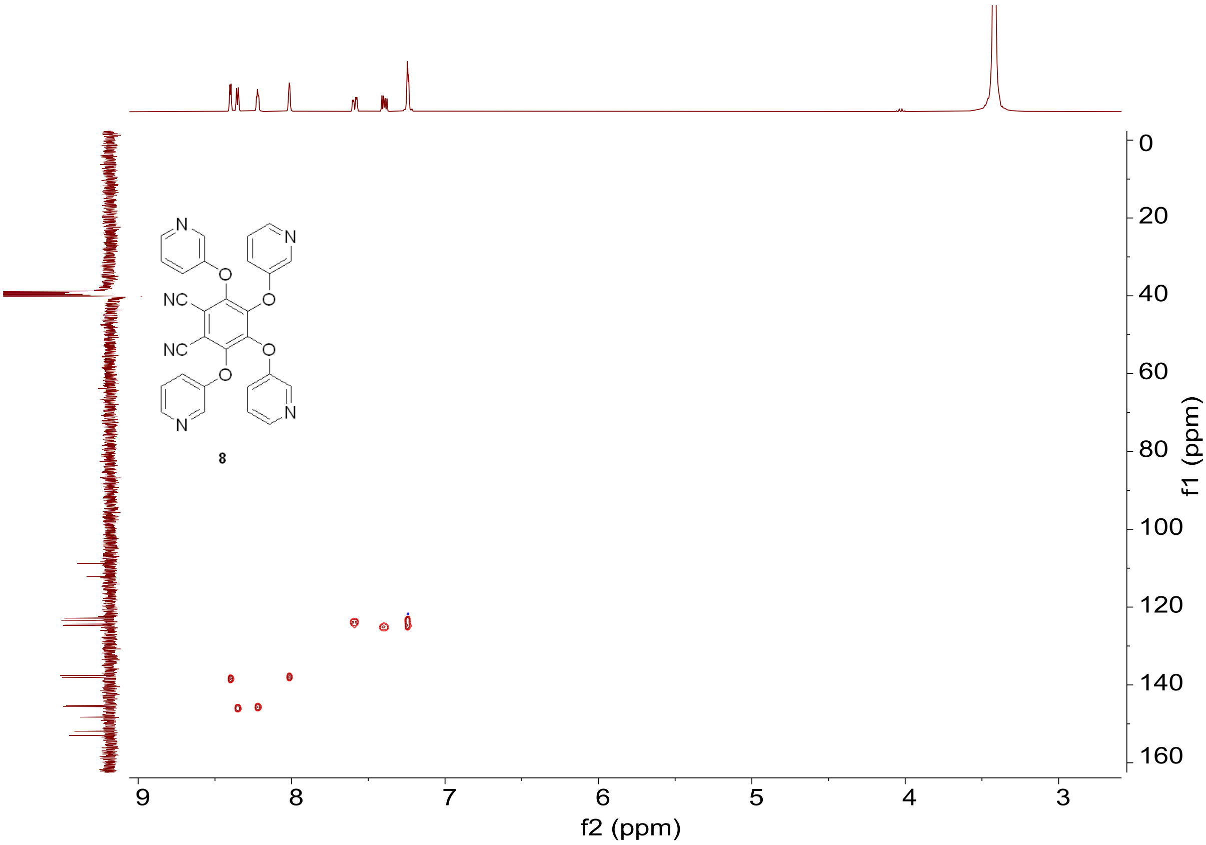

Figure S54: HSQC spectrum of **8**.

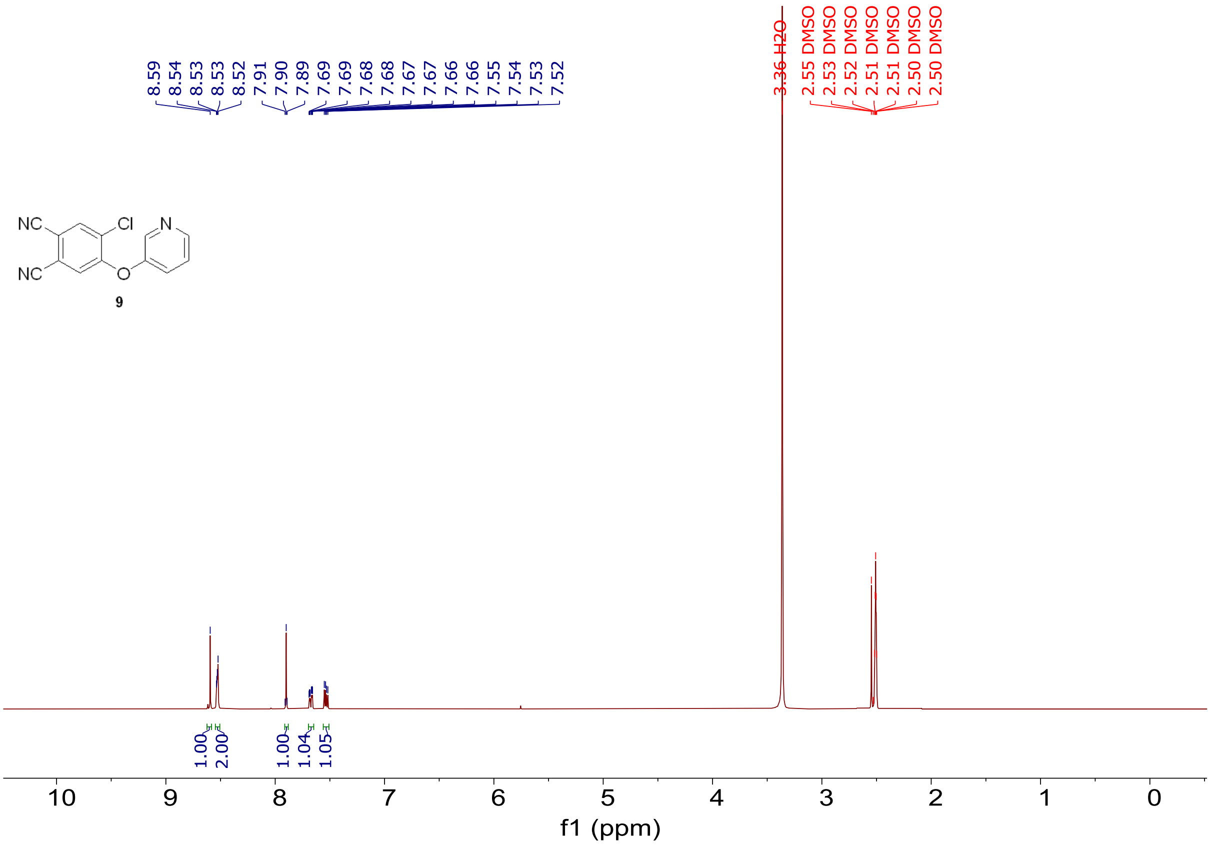

Figure S55: ^{1}H spectrum of **9**.

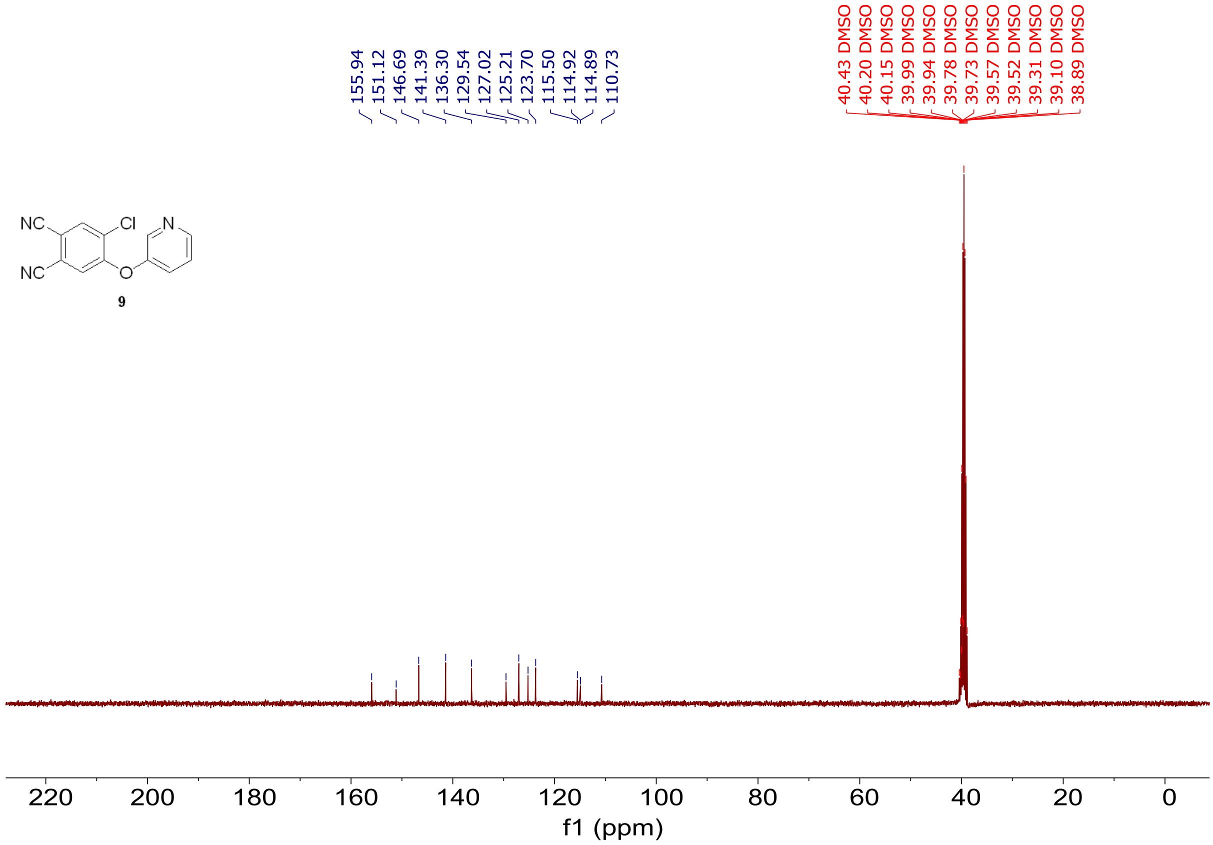

Figure S56: ^{13}C spectrum of **9**.

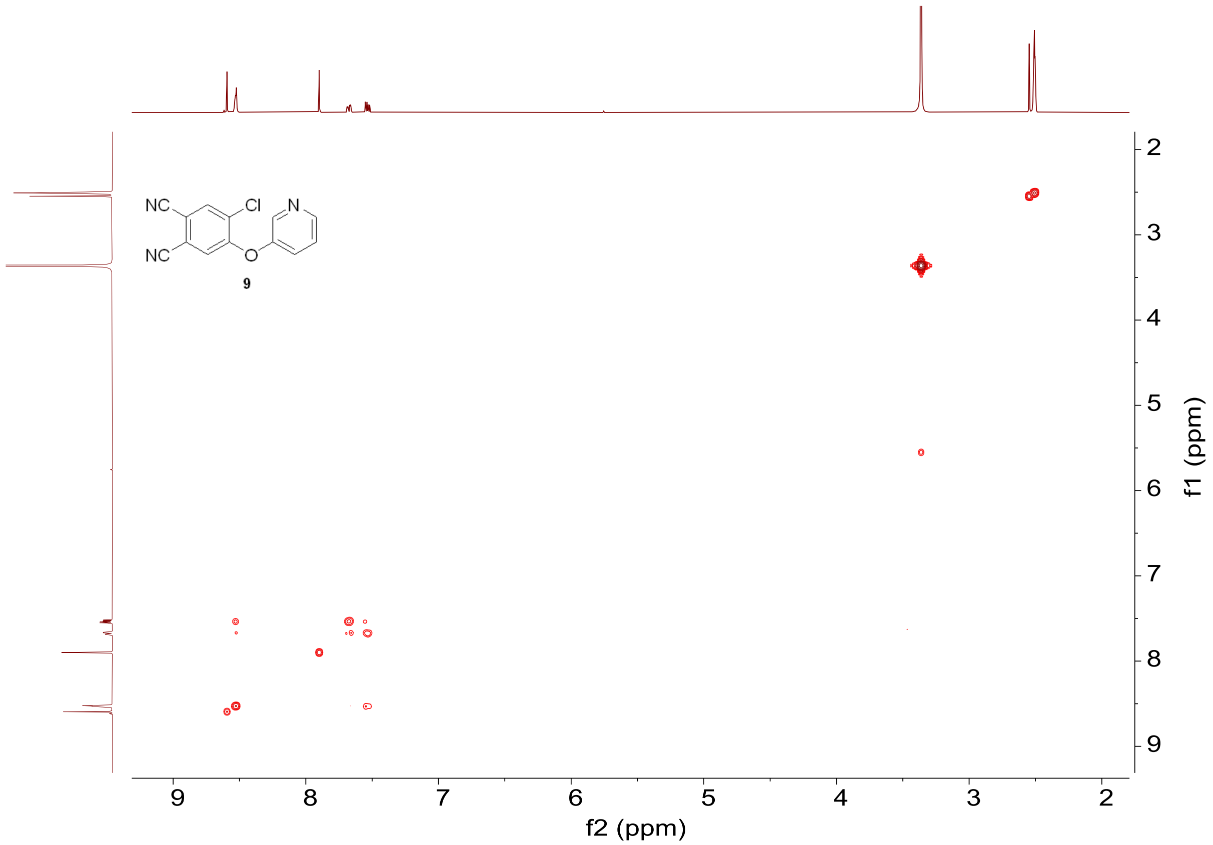

Figure S57: COSY spectrum of **9**.

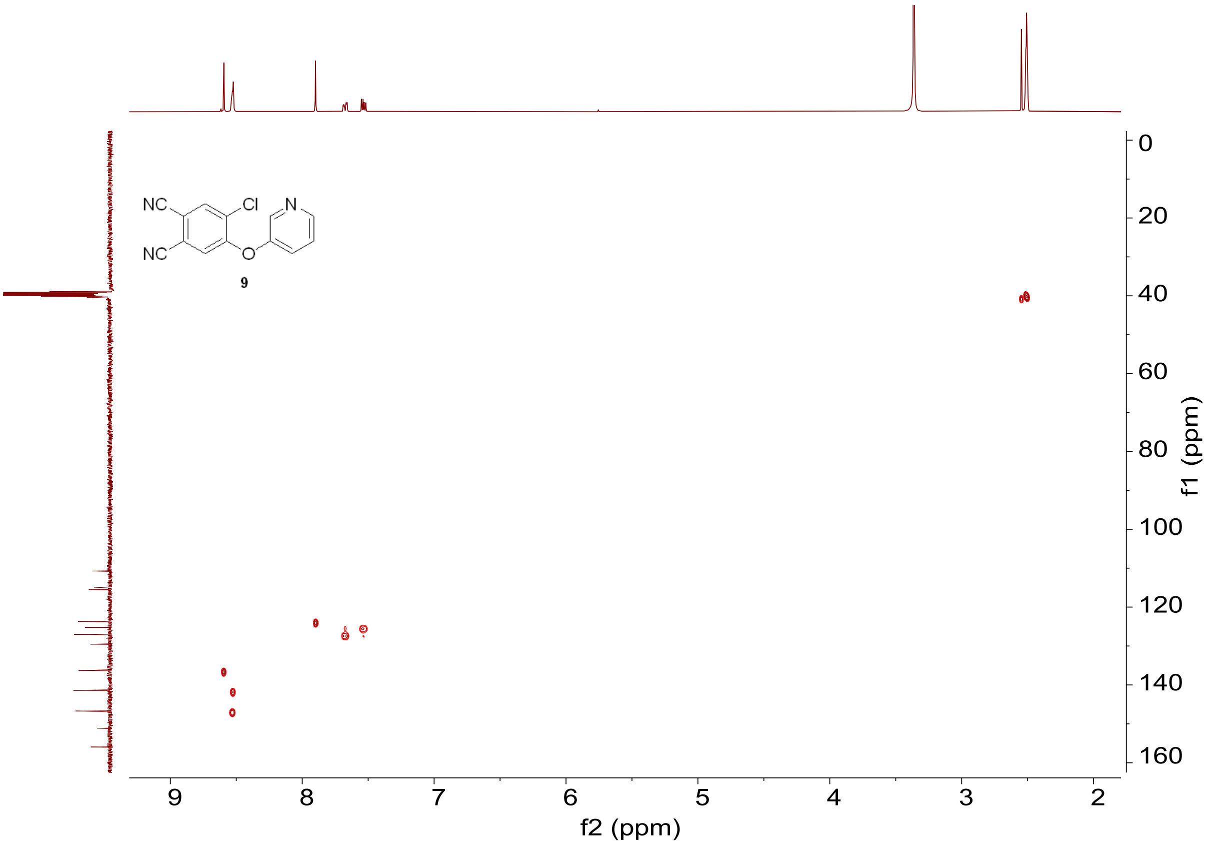

Figure S58: HSQC spectrum of **9**.

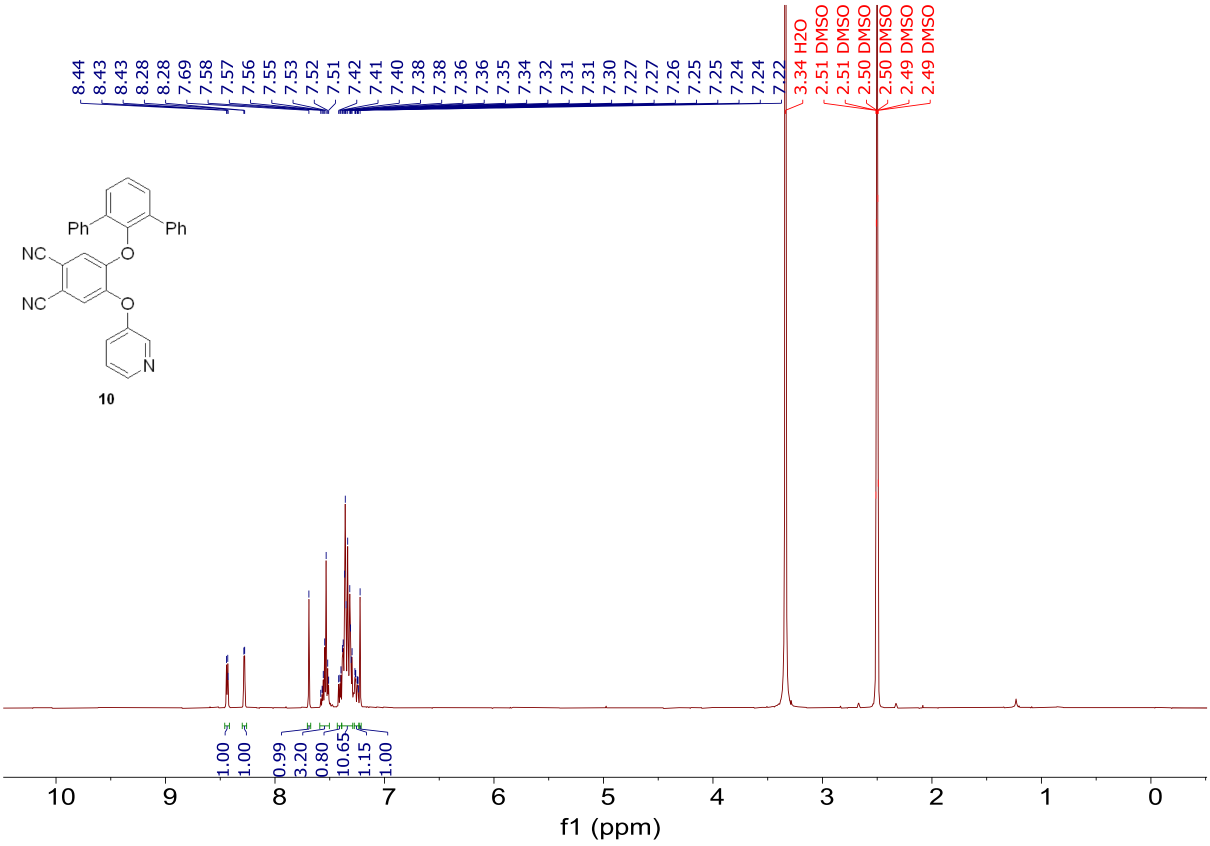

Figure S59: ^{1}H spectrum of **10**.

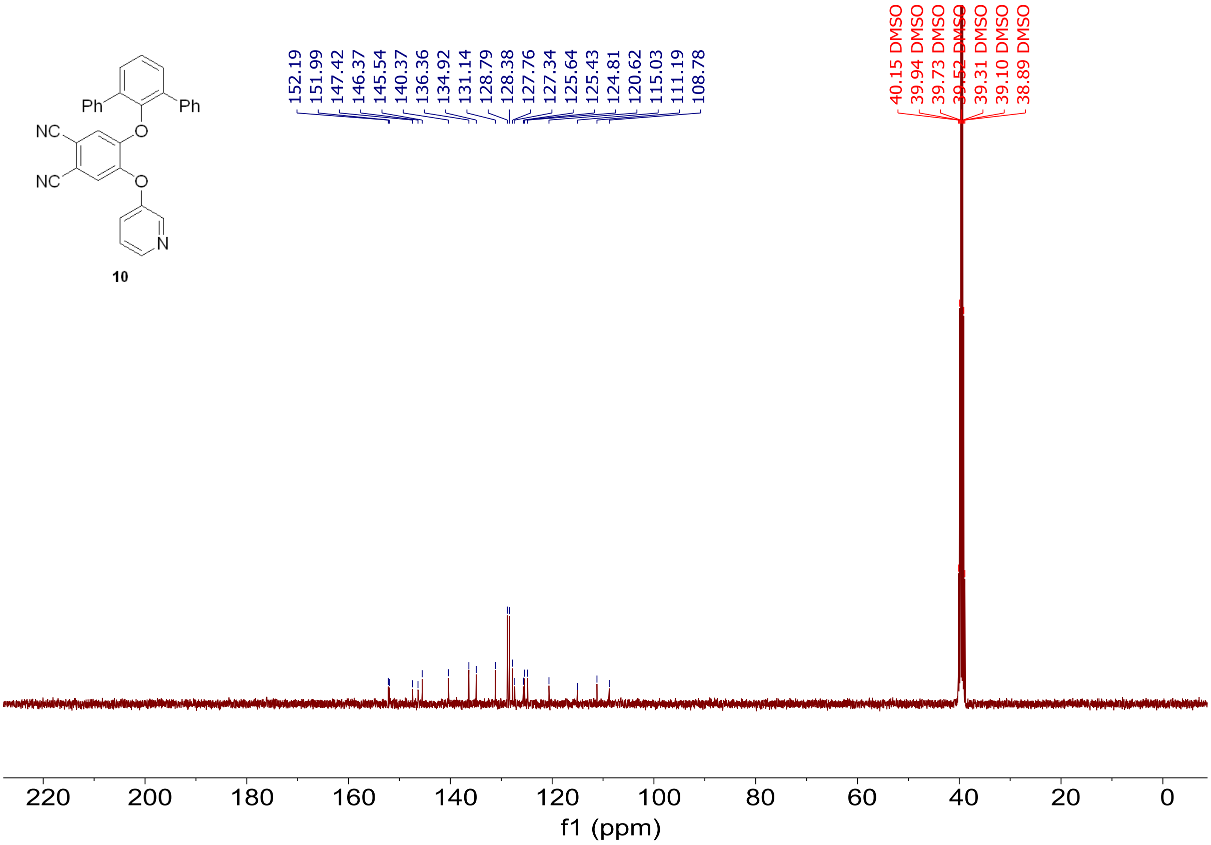

Figure S60: ^{13}C spectrum of **10**.

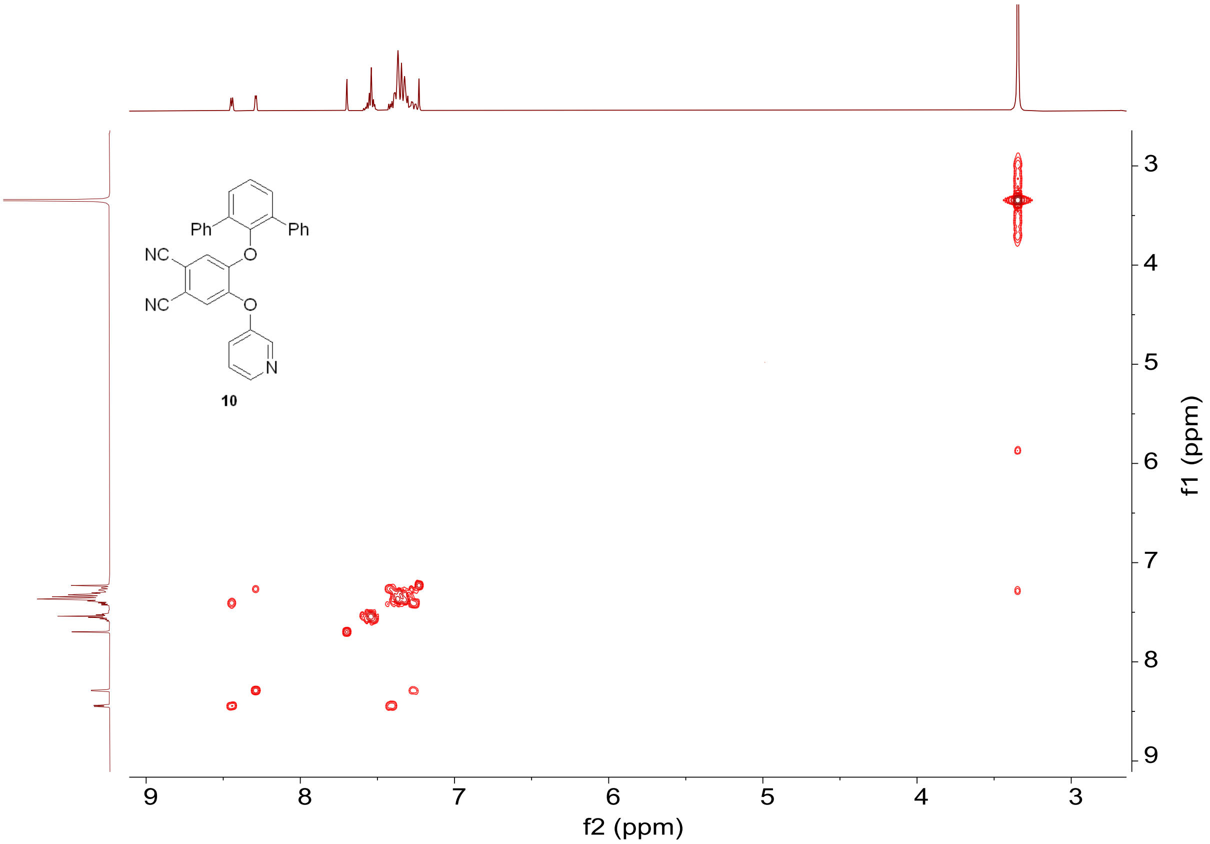

Figure S61: COSY spectrum of **10**.

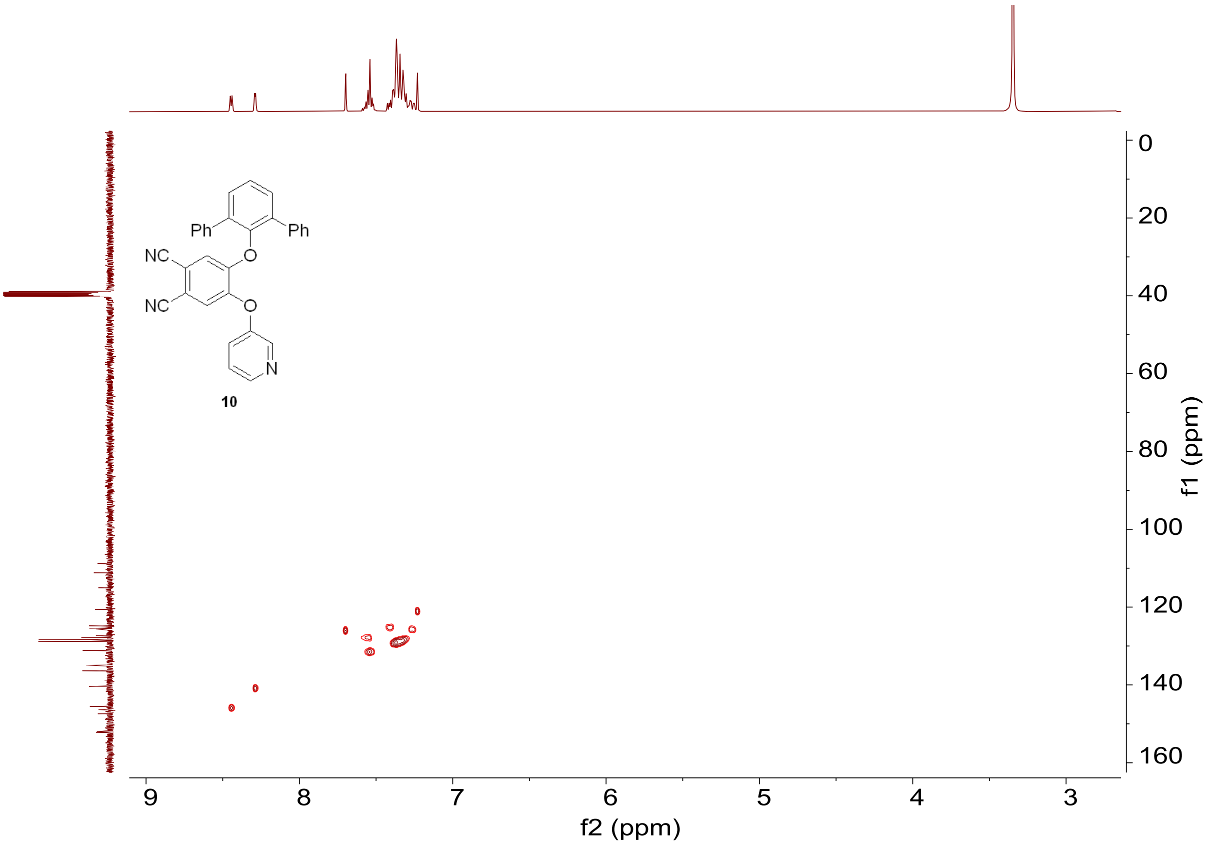

Figure S62: HSQC spectrum of **10**.

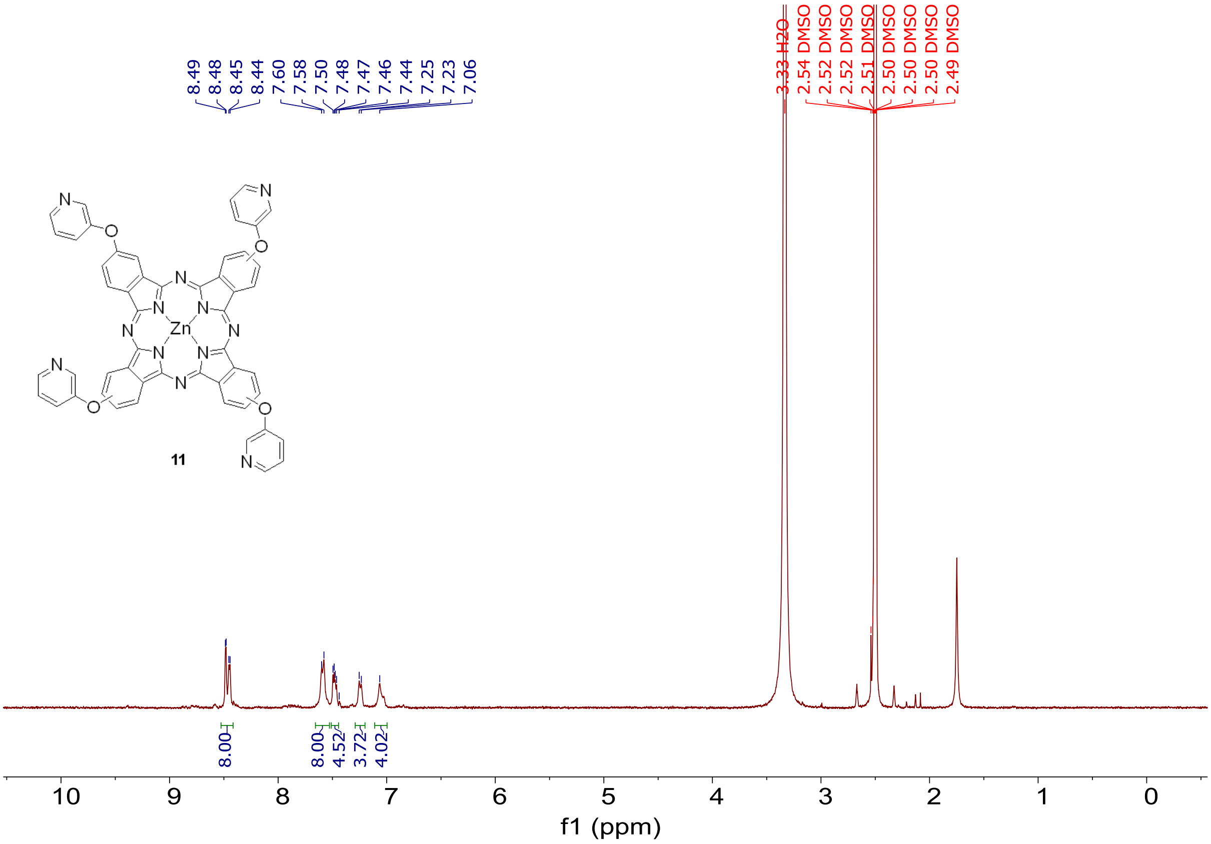

Figure S63: ^{1}H spectrum of **11** from one-pot reaction.

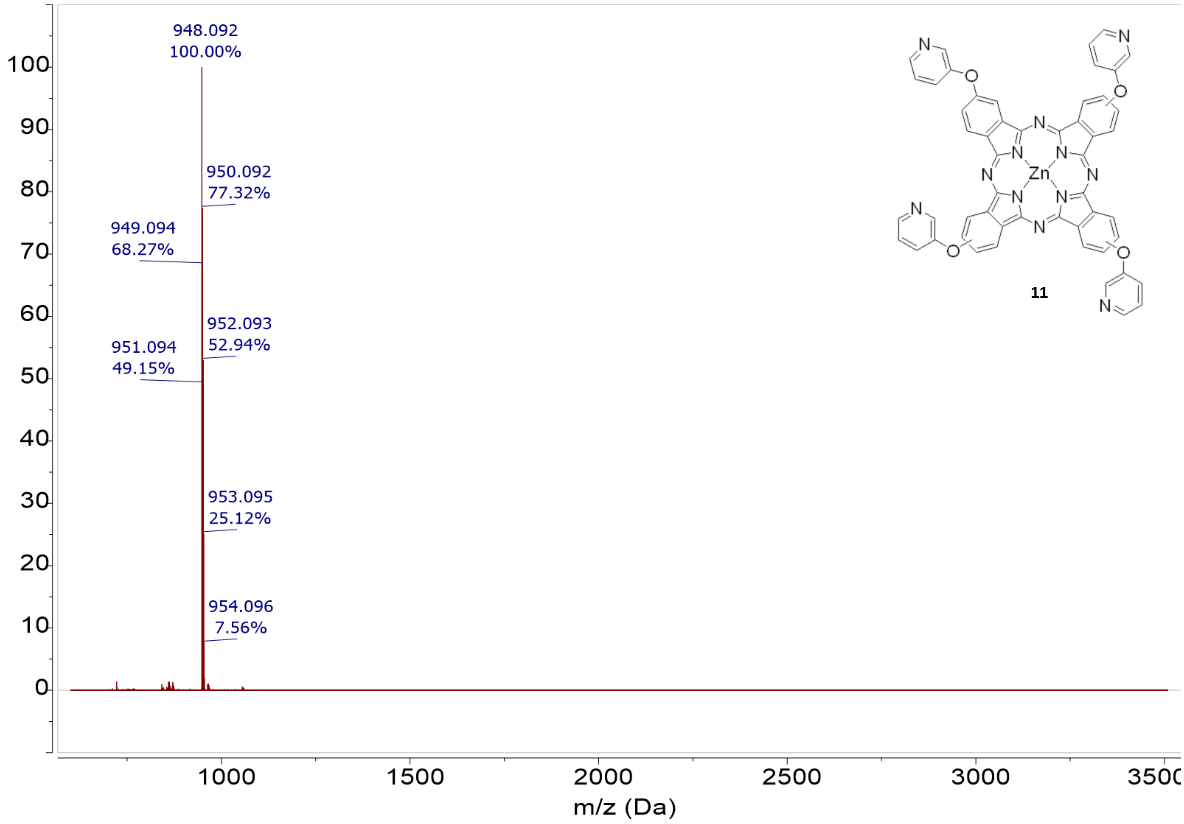

Figure S64: MALDI spectrum of **11**.

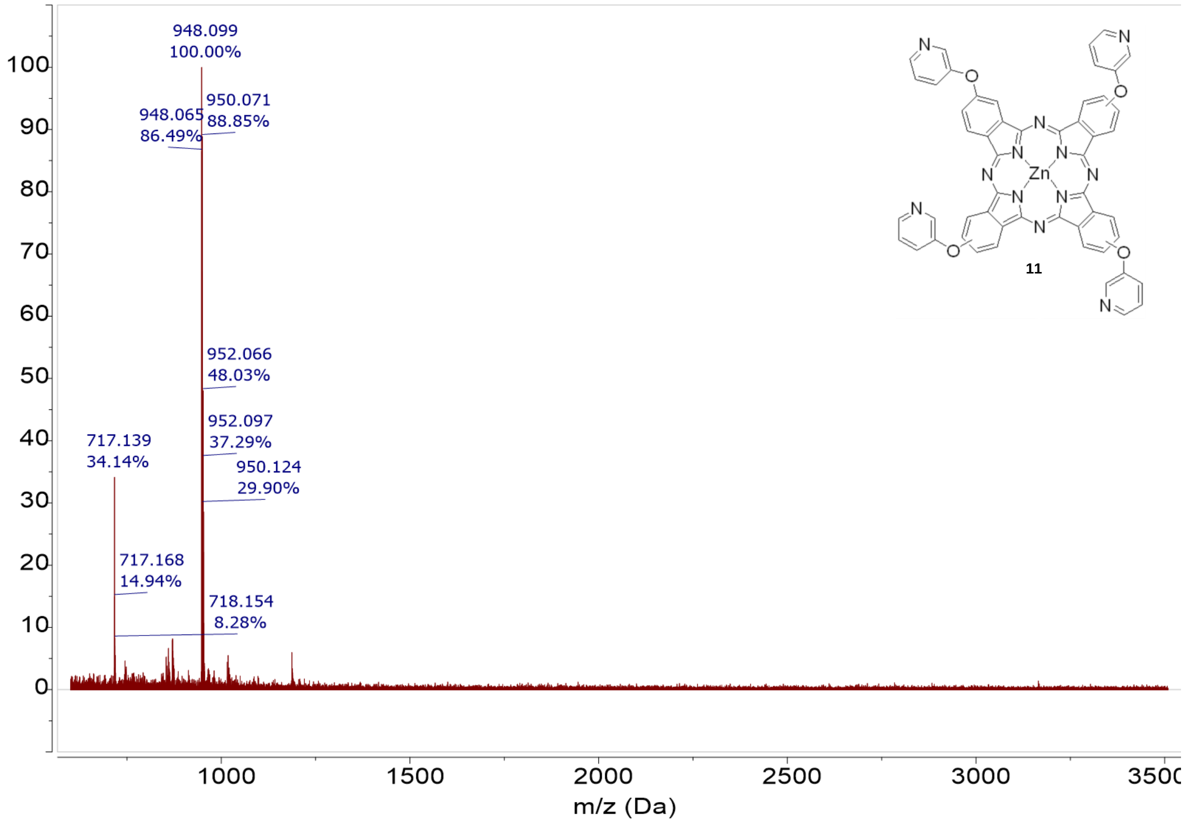

Figure S65: MALDI spectrum of **11** for one-pot reaction.

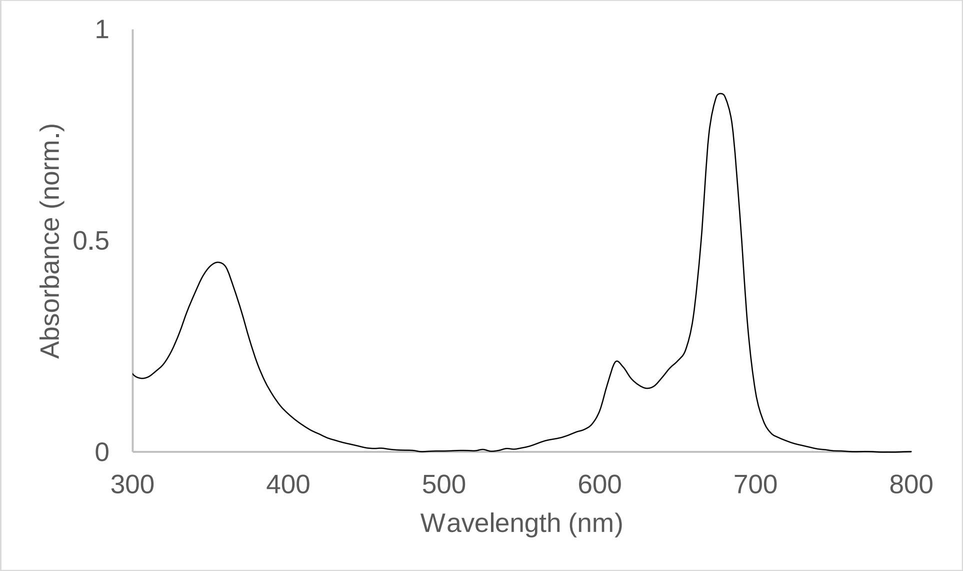

Figure S66: UV-Vis absorption spectrum of **11** between 300-800 nm.

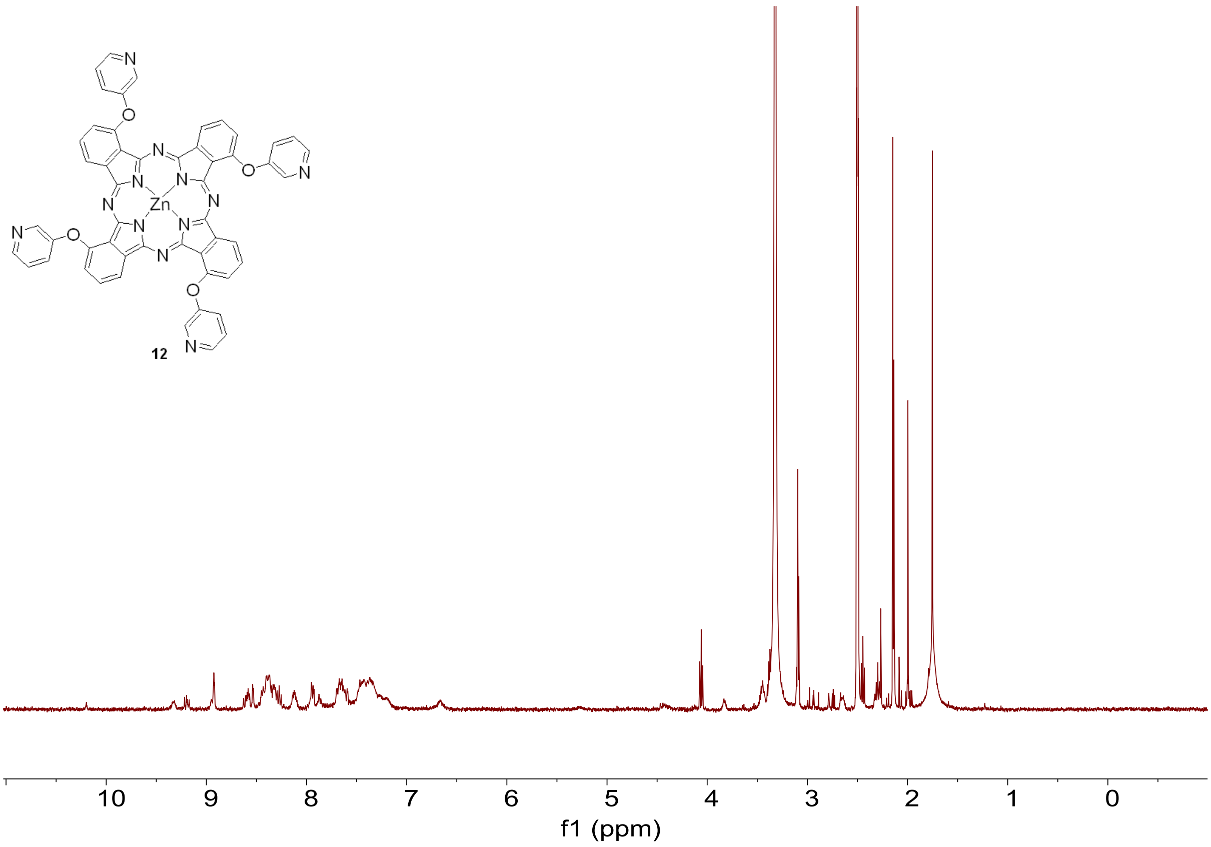

Figure S67: ^{1}H spectrum of **12**.

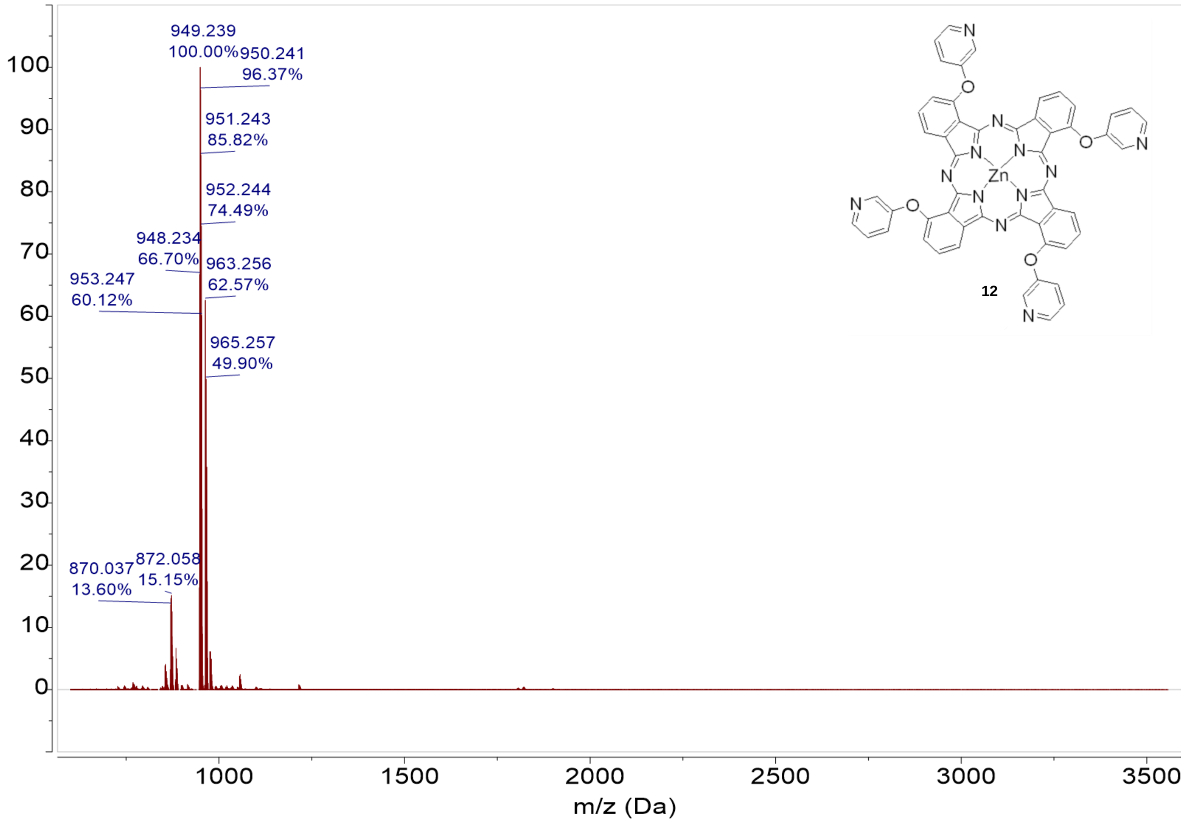

Figure S68: MALDI spectrum of **12.**

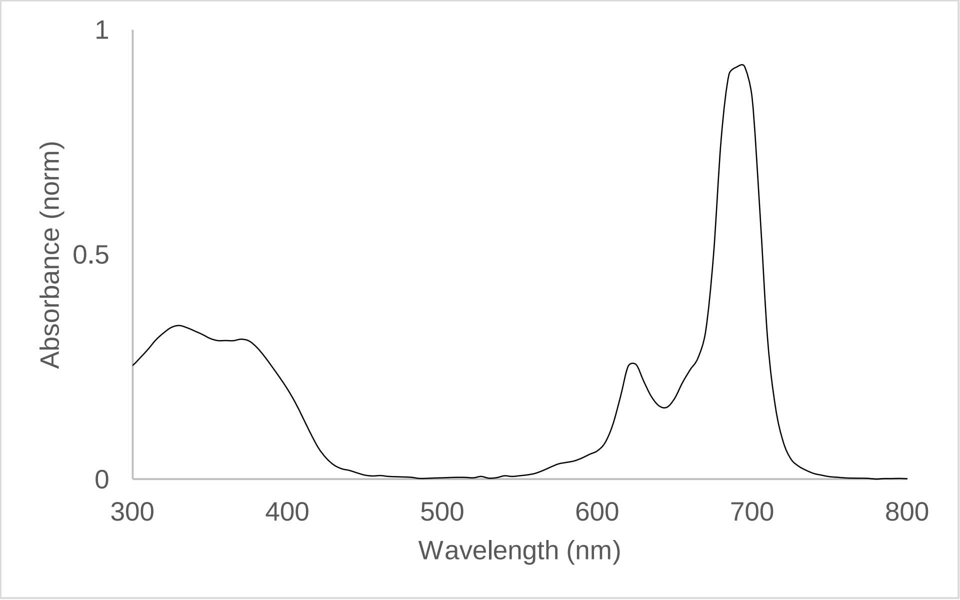

Figure S69: UV-Vis absorption spectrum of **12** between 300-800 nm.

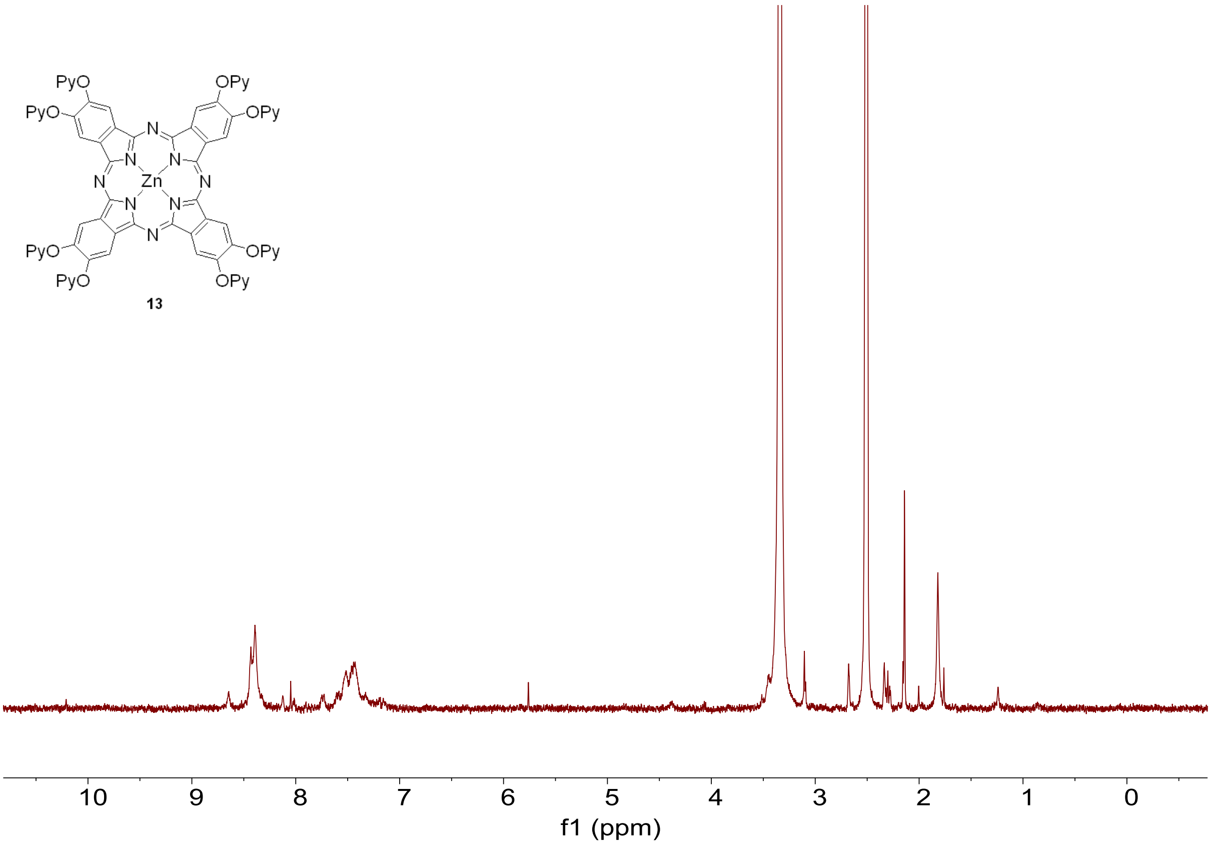

Figure S70: ^{1}H spectrum of **13**.

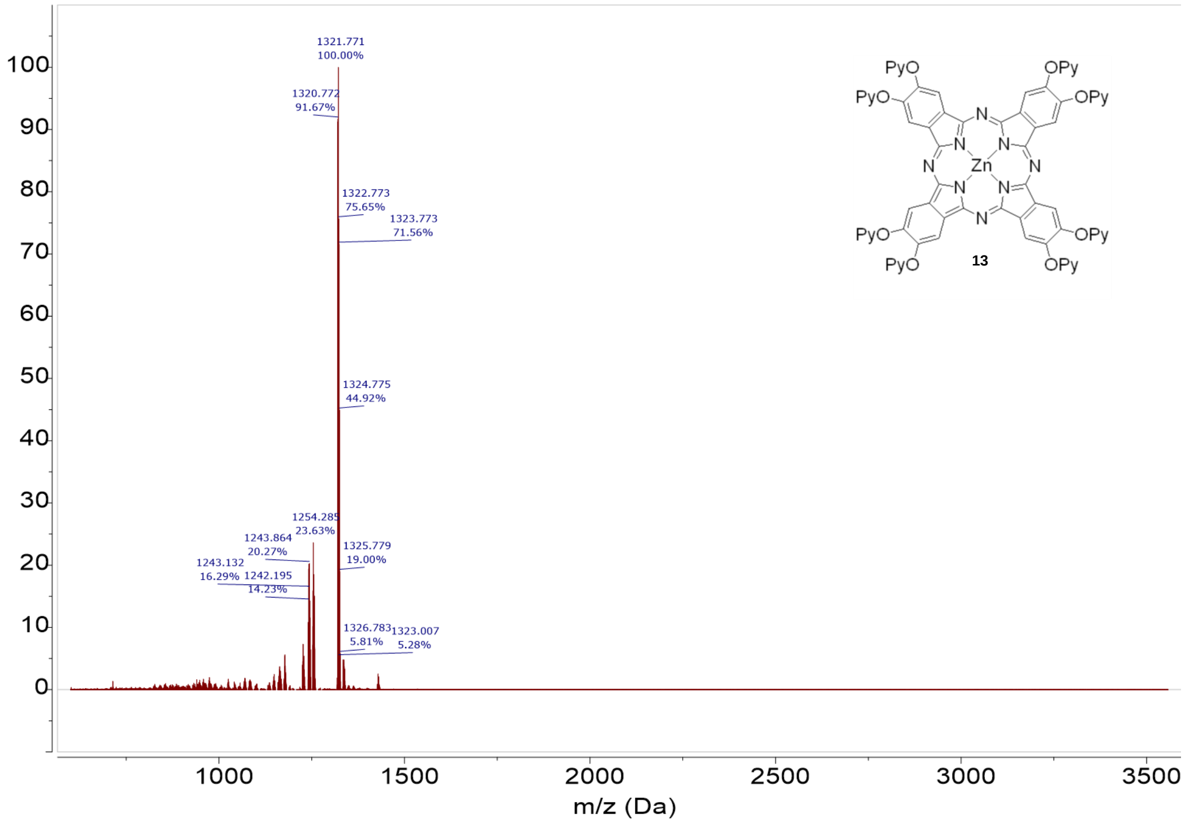

Figure S71: MALDI spectrum of **13.**

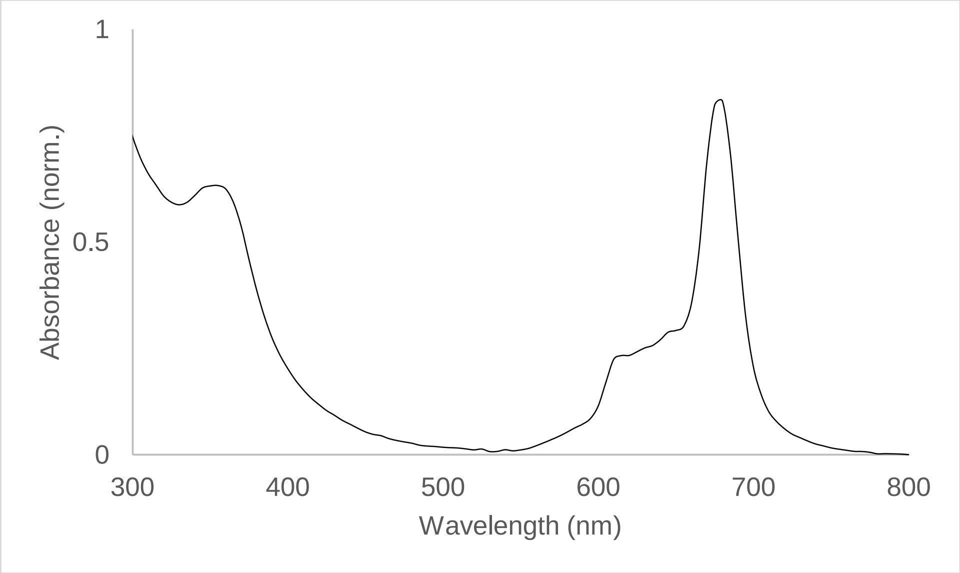

Figure S72: UV-Vis absorption spectrum of **13** between 300-800 nm.

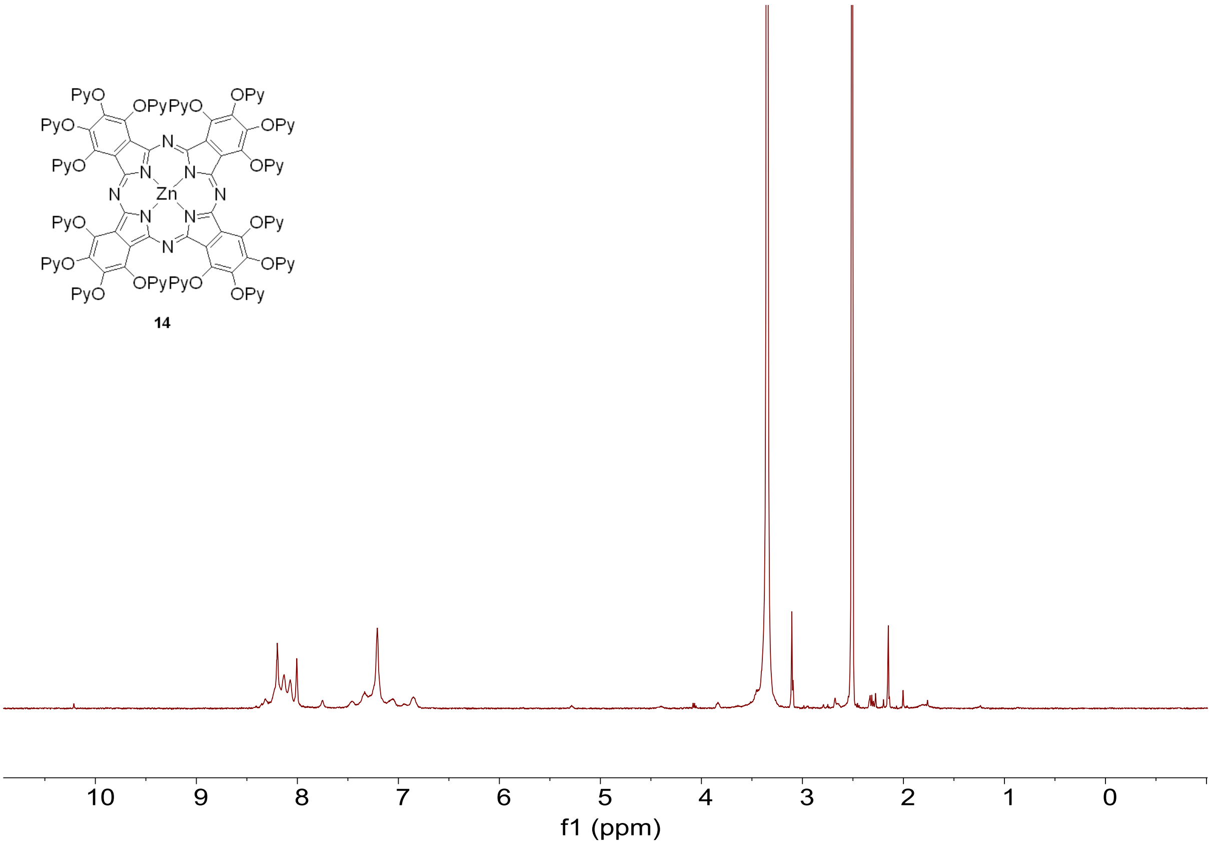

Figure S73: ^{1}H spectrum of **14**.

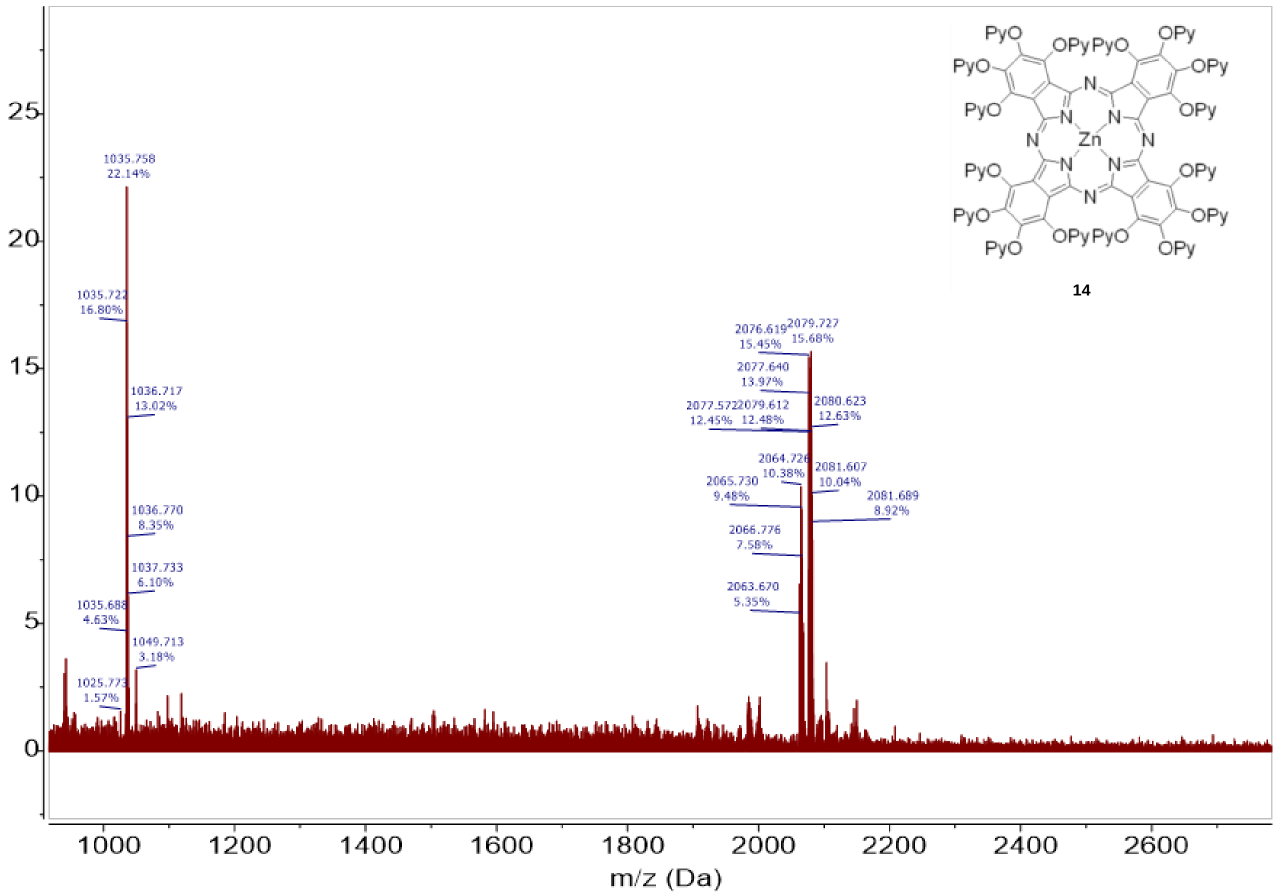

Figure S74: MALDI spectrum of **14.**

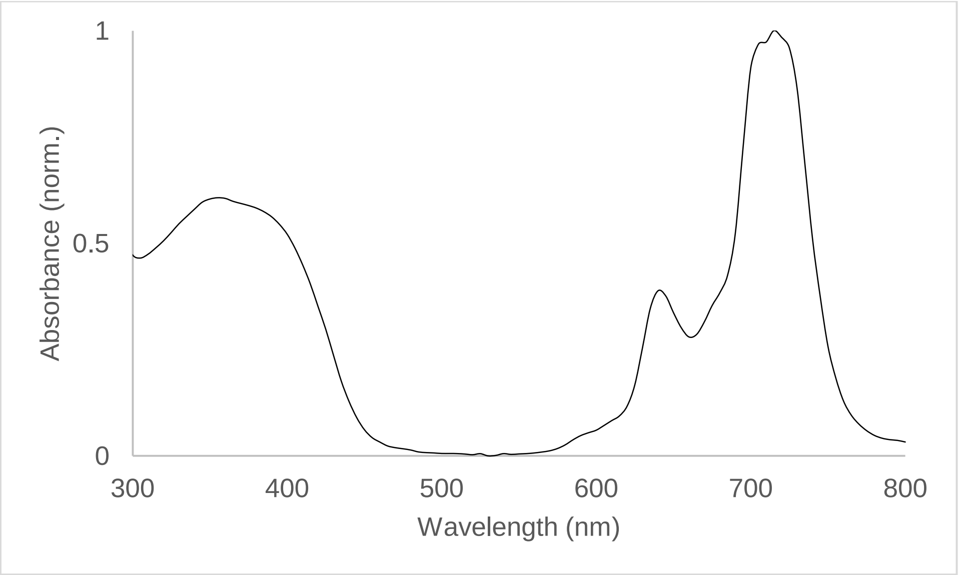

Figure S75: UV-Vis absorption spectrum of **14** between 300-800 nm.

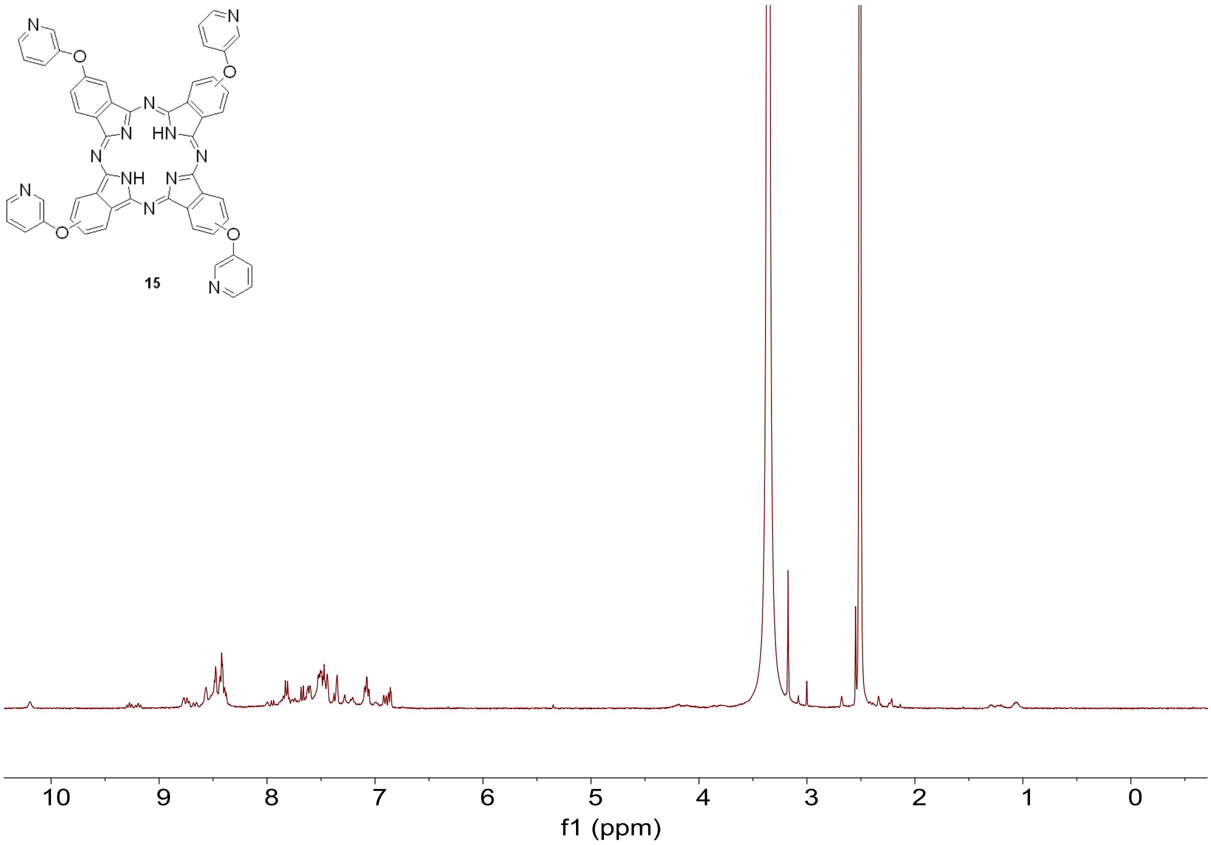

Figure S76: ^{1}H spectrum of **15**.

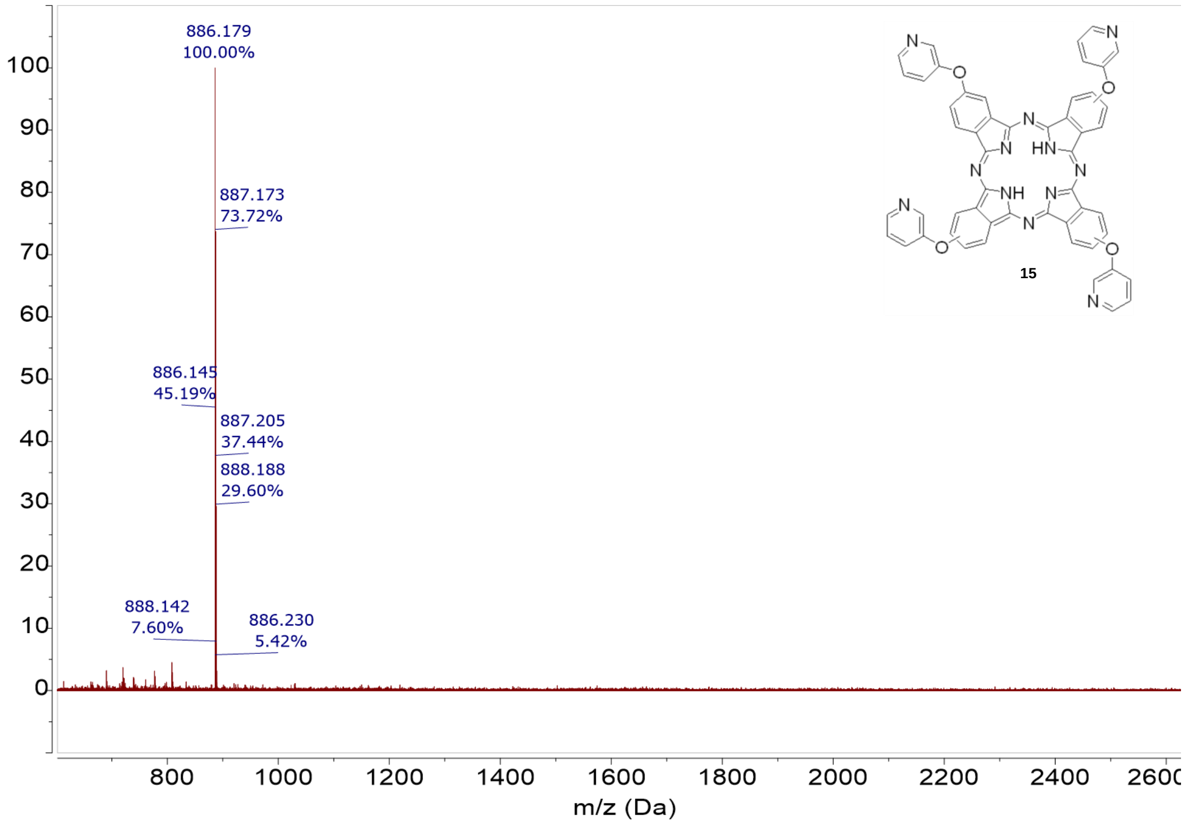

Figure S77: MALDI spectrum of **15.**

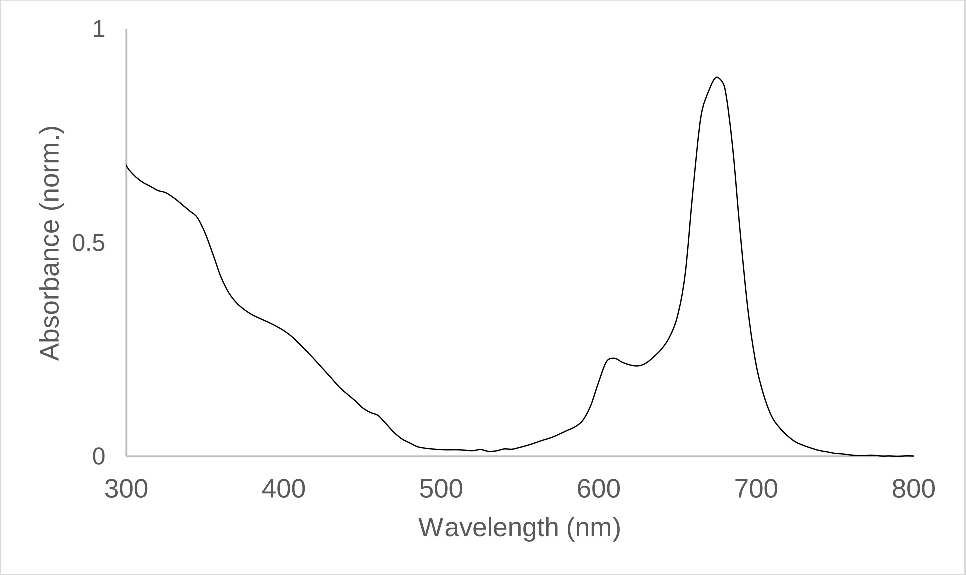

Figure S78: UV-Vis absorption spectrum of **15** between 300-800 nm.

12. References

[1] R.A. Sheldon, "The E factor at 30: a passion for pollution prevention" *Green Chem.,* **2023**, 25, 1704-1728
